# Cumulative incidence of chronic health conditions recorded in hospital inpatient admissions from birth to age 16 in England

**DOI:** 10.1093/ije/dyae138

**Published:** 2024-10-10

**Authors:** Matthew A Jay, Lauren Herlitz, Jessica Deighton, Ruth Gilbert, Ruth Blackburn

**Affiliations:** University College London Great Ormond Street Institute of Child Health, London, UK; University College London Great Ormond Street Institute of Child Health, London, UK; Evidence Based Practice Unit, UCL and Anna Freud Centre for Children and Families, London, UK; University College London Great Ormond Street Institute of Child Health, London, UK; University College London Great Ormond Street Institute of Child Health, London, UK

**Keywords:** Chronic health conditions, administrative data, data linkage, population spine, population denominator, cumulative incidence

## Abstract

**Background:**

Monitoring the incidence of chronic health conditions (CHCs) in childhood in England, using administrative data to derive numerators and denominators, is challenged by unmeasured migration. We used open and closed birth cohort designs to estimate the cumulative incidence of CHCs to age 16 years.

**Methods:**

In closed cohorts, we identified all births in Hospital Episode Statistics (HES) from 2002/3 to 2011/12, followed to 2018/19 (maximum age 8 to 16 years), censoring on death, first non-England residence record or 16th birthday. Children must have linked to later HES records and/or the National Pupil Database, which provides information on all state school enrolments, to address unmeasured emigration. The cumulative incidence of CHCs was estimated to age 16 using diagnostic codes in HES inpatient records. We also explored temporal variation. Sensitivity analyses varied eligibility criteria. In open cohorts, we used HES data on all children from 2002/3 to 2018/19 and national statistics population denominators.

**Results:**

In open and closed approaches, the cumulative incidence of ever having a CHC recorded before age 16 among children born in 2003/4 was 25% (21% to 32% in closed cohort sensitivity analyses). There was little temporal variation. At least 28% of children with any CHC had more than one body system affected by age 16. Multimorbidity rates rose with later cohorts.

**Conclusions:**

Approximately one-quarter of children are affected by CHCs, but estimates vary depending on how the denominator is defined. More accurate estimation of the incidence of CHCs requires a dynamic population estimate.

Key Messages

- We aimed to estimate the cumulative incidence of chronic health conditions (CHCs) recorded in administrative hospital admissions data to age 16 in England.
- Of all children born in England in 2003/4, 25%had a CHC recorded in their hospital admission records before they turned 16, varying from 21% to 32% in sensitivity analyses.
- These are lower-bound estimates of the cumulative incidence of CHCs in the community and represent a very high burden on children and families, schools, health and social care services across the child life course.

## Introduction

Chronic health conditions (CHCs) in childhood and adolescence affect quality of life and need for services.^1–4^ The UK government’s Major Conditions Strategy sets out the need to monitor the growing proportion of people with CHCs, including multimorbidity.^5^ Monitoring requires consistent measures so that changes in CHC burden due to policy, services and upstream determinants, (e.g. environmental hazards, economic stresses and access to healthy food) can be monitored, and people most at risk can be targeted by prevention, support or treatment strategies.

The most effective time to intervene to prevent or mitigate CHCs is in childhood, when many conditions emerge.^6^ Early intervention, by universal health care and education services that see children repeatedly across their development, requires evidence on the total proportion of children ever affected by CHCs and the age when first recorded by services (i.e. the cumulative incidence), over time and for different types of CHCs. Services need this information to resource the management of these conditions and their effects on health and participation in education.^7^

We used administrative hospital data (the Hospital Episode Statistics; HES) to measure the cumulative incidence of CHCs throughout childhood.^8^ HES records all NHS hospital contacts in England, primarily for the purposes of financial reimbursement, and captures CHCs recorded at discharge from a day case or from longer admission to an NHS hospital. We focus on hospital administrative data, rather than primary care data or consented studies or surveys, for the reasons given in Table 1. Because of a lack of a dynamic denominator population in HES (and absence of migration data), we developed denominators drawn from birth records in HES and from education data for all children in England, using the Education and Child Health Insights from Linked Data (ECHILD) database.^9^ We required linkage of birth records to later HES or National Pupil Database (NPD)^10^ records and varied these requirements in sensitivity analyses to assess variation in the estimated cumulative incidence of CHCs up to age 16. We compare results with an open cohort approach using HES numerator data and Office for National Statistics (ONS) mid-year population denominator estimates. We also report the cumulative incidence of CHCs affecting different body systems, and the proportion of children with more than one body system affected.

**Table 1.** Possible data sources and their principal advantages and disadvantages

Data source	Advantages	Disadvantages
Consented studies or surveys	Case ascertainment not dependent on health care usage.	Subject to selection and attrition bias; cannot be repeated easily for routine monitoring
Primary care administrative data	CHCs can be phenotyped in rich diagnostic data; more representative of CHCs in the community than secondary care data; easily updatable for monitoring	Does not currently cover whole population; subject to loss to follow-up when patients change GP practices^30^^,^^31^
Secondary care administrative data (Hospital Episode Statistics)	CHCs can be phenotyped in rich diagnostic data; covers all NHS secondary care services (including 98% of births); easily updatable for monitoring	Lack of a suitable denominator population: in Wales and Scotland, the NHS spine, which defines anyone with an NHS number who is registered with a GP or attends hospital or community services, is used to define the denominator population of people who could use hospital services, but this is not available in England. Estimates based on extrapolations from decennial census for England^32^ have been shown to vary significantly overall and by demographic factors from estimates based on GP registrations^33^^,^^34^

CHCs, chronic health conditions; GP, general practitioner; NHS, National Health Service.

## Methods

### Method 1: data source and denominator population

We used closed birth cohorts based on HES inpatient records^8^ to define the denominator population with follow-up to the 16th birthday. Birth episodes were identified using age and other variables indicating birth.^11^ Children live-born in England in academic years (September to August) 2002/3 to 2011/12 were eligible. Follow-up ceased on 31 August 2019 to avoid confounding by the COVID-19 pandemic in the school year 2019/20 (maximum per-cohort age 8 to 16 years at final follow-up).

We included all children with a live birth in England recorded in HES and who linked to any later HES activity before age 16 and/or who linked to NPD records. Linkage to later HES and/or NPD activity addresses loss to follow-up due to linkage error between birth and later records, and increases the likelihood that we include children who remain in the country following birth.^12^ For the purposes of identifying eligible children, HES activity included admission records (available from 2002/3), outpatients data (from 2004/5) and Accident and Emergency records (from 2007/08). Activity in NPD, which captures all children in English state schools (>97%), included enrolments (recorded up to three times per year) and/or participation in mandatory public examinations at ages 4/5, 6/7, 10/11 or 15/16.^9^^,^^10^ We used the highest quality link for each child to derive a 1:1 linkage spine between HES and NPD in the ECHILD database.^13^

### Defining the numerator: chronic health conditions

In HES, diagnostic codes are recorded by trained clinical coders on patient discharge according to national standards. We used a validated list of International Classification of Diseases 10th Revision (ICD-10) codes to identify CHCs recorded in any inpatient episode in the primary or any secondary position (i.e. including up to 20 ICD-10 codes per episode).^14^ Hardelid *et al*.^14^ developed their code list in order to detect CHCs defined as ‘any health problem likely to require follow-up for more than 1 year, where follow-up could be repeated hospital admission, specialist follow-up through outpatient department visits, medication or use of support services’.^14^ Codes from previously validated lists were used to develop the Hardelid *et al*.^14^ code list, as well as additional codes based on hospital discharge data and searches of the ICD-10 dictionary, and validity was assessed in consultation with five clinicians. The first recorded instance of a CHC code in an HES episode per child, including the birth episode, was counted as an incident case. CHCs were grouped according to Hardelid *et al.*’s^14^ subtypes by reference to body systems affected. A machine-readable *.CSV file containing all codes by each group is available in Supplementary Table S1 (available as Supplementary data at *IJE* online) and can also be accessed via the ECHILD Phenotype Code List Repository at [https://code.echild.ac.uk/chc_hardelid_v1]. We did not use HES outpatients or Accident and Emergency records to capture CHCs, due to a lack of clinical coding in these data. HES does not contain primary care or prescribing data.

### Statistical analysis

The outcome was the cumulative incidence of being admitted to hospital with a record indicating a CHC before age 16, estimated as one minus the survival function, censoring at whichever came first of: death (ONS death registrations); first HES contact where domicile was recorded as outwith England; or 16th birthday. The cumulative incidence was estimated in each birth cohort from 2002/3 to 2011/12.

We then calculated the cumulative incidence of the body system subtypes in each cohort. Due to small cell counts, we grouped birth cohorts into pairs: 2002/3 and 2003/4 (followed to age 16); 2004/5 and 2005/6 (to age 14); 2006/7 and 2007/8 (to age 12); 2008/9 and 2009/10 (to age 10); 2010/11 and 2011/12 (to age 8). We do not report data for chronic infections due to very low numbers of children affected.

To examine multimorbidity, we calculated the proportions of children with two or more CHC subtypes ever recorded by age 16.

We re-estimated the cumulative incidence of any CHC treating death as a competing risk, rather than a censoring event. Results were identical and are not presented.

### Sensitivity analyses

To investigate the extent to which varying eligibility criteria (for example, by not requiring later HES or NPD activity) may influence cumulative incidence estimates, we varied eligibility requirements (Table 2) and re-estimated the cumulative incidence of any CHC and each subtype. Eligibility criteria were specified on the assumption that allowing for more activity in HES or NPD would mean children in the country and eligible for services are more likely to be included. However, this is balanced against the fact that more health care activity would be associated with children who are sicker, thereby upwardly biasing CHC incidence estimates.

**Table 2.** Requirements for inclusion in the main and sensitivity analyses

Shorthand	Live birth in England in HES APC	**Subsequent HES record before age 5** ^a^	**Subsequent HES record age 5 to before age 16** ^a^	**Subsequent HES record before age 16** ^a^	**Links to NPD** ^b^
Main analysis^c^					
Birth record and HES <16 or birth record and NPD	✓			✓ (or NPD)	✓ (or HES)
**Sensitivity analyses**					
1. HES birth record only	✓				
2. Birth and HES <5	✓	✓			
3. Birth and HES <16	✓			✓	
4. Birth and NPD	✓				✓
5. Birth and HES <5 and NPD	✓	✓			✓
6. Birth and HES <5 and HES 5 to <16	✓	✓	✓		
7. Birth and HES <5 and HES 5 to <16 and NPD	✓	✓	✓		✓

APC, Admitted Patient Care; HES, Hospital Episode Statistics; NPD, National Pupil Database.

a Subsequent HES records include APC, outpatients and Accident and Emergency.

b NPD records included all enrolments and public examinations.

c <5, relevant activity observed at any point before age 5 years; <16, relevant activity observed at any point before age 16 years; 5 to <16, relevant activity observed from age 5 years to before age 16 years.

### Method 2: Office for National Statistics denominators

Method 1 (closed birth cohorts) attempts to account for unmeasured migration by linking to later education records, but this does not account for inward migration; as the cohort ages, the difference between the birth cohort and the resident child population grows wider. We therefore estimated the cumulative incidence of any CHC and each CHC subtype (ascertained as in Method 1) in open cohorts of children born in financial years 2002/3 to 2018/19, regardless of place of birth, followed to 2019/20. We used ONS mid-year population estimates as estimates of the population of each cohort at each year of age, discounted by the cumulative number of children with a CHC. The cumulative incidence by each year of age was calculated by as 1-exp⁡(-IH), where IH = the integrated (cumulative) hazard of a CHC to the relevant age and the hazard is calculated as the risk of an incident CHC code at each age.

We used R 4.0.2 and 4.3.0 and the packages listed in Supplementary Table S2 (available as Supplementary data at *IJE* online).

## Results

### Method 1 (closed birth cohorts): linkage rates and demographics

From 2003/4, 423 456 children were included (74.9% of all births and 79.4% of all HES birth episodes), rising to 618 054 by 2011/12 (89.9% of births and 97.5% of all HES birth episodes; Table 3). Linkage rates thus improved over time, despite having less follow-up. Supplementary Figures S1 and S2 (available as Supplementary data at *IJE* online) show earlier detection in later cohorts, particularly following the availability of Accident and Emergency data from 2007/8. There were minimal differences in demographics or clinical details among birth cohorts (Supplementary Table S3, available as Supplementary data at *IJE* online) or when varying eligibility criteria in sensitivity analyses (Supplementary Table S4, available as Supplementary data at *IJE* online).

**Table 3.** Numbers of live births in England, births recorded in Hospital Episode Statistics and children included in the study (main analyses) in each birth cohort

Academic year of birth	**ONS live births** ^a^ ***n***	Births recorded in HES APC *n* (% live births)	**Included in the study** ^b^		
			*n*	(% live births)	(% births in HES)
2002/3	565 709	533 406 (94.3%)	423 456	(74.9%)	(79.4%)
2003/4	589 851	547 636 (92.8%)	485 301	(82.3%)	(88.6%)
2004/5	607 184	557 927 (91.9%)	508 945	(83.8%)	(91.2%)
2005/6	613 028	578 446 (94.4%)	533 446	(87.0%)	(92.2%)
2006/7	635 748	600 595 (94.5%)	557 667	(87.7%)	(92.9%)
2007/8	655 357	624 933 (95.4%)	562 596	(85.8%)	(90.0%)
2008/9	672 809	633 574 (94.2%)	594 510	(88.4%)	(93.8%)
2009/10	671 058	648 371 (96.6%)	625 122	(93.2%)	(96.4%)
2010/11	687 077	649 636 (94.6%)	628 452	(91.5%)	(96.7%)
2011/12	688 120	633 670 (92.1%)	618 054	(89.8%)	(97.5%)

HES APC, Hospital Episode Statistics Admitted Patient Care; ONS, Office for National Statistics.

a ONS vital statistics.^35^

b Children were included if they had a live birth in HES (column 3) and linked to later HES records before age 16 years and/or National Pupil Database records. Later HES records included all available APC, outpatients and Accident and Emergency Data. National Pupil Database records included all enrolments and public examinations.

### Cumulative incidence

**Figure 1.**
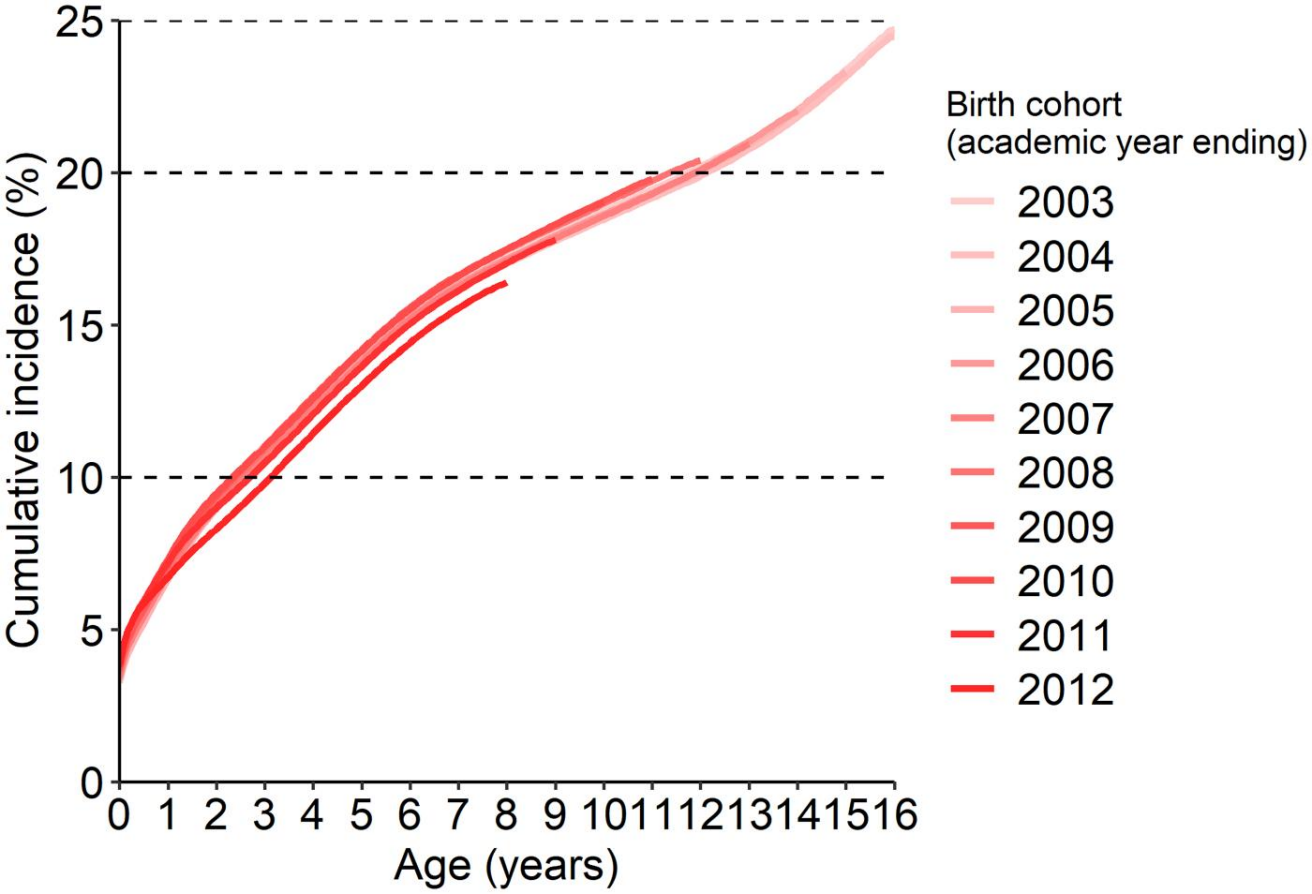
Cumulative incidence of being admitted to hospital and having any record indicating a chronic health condition before age 16 (closed birth cohorts). Horizontal guide lines are at 10%, 20% and 25%. Academic year = 1 September to 31 August. Underlying percentages are available in Supplementary Table S5 (available as Supplementary data at *IJE* online)

The cumulative incidence of being admitted with any CHC by age 16 in the 2003/4 birth cohort was 24.5% (Figure 1;Supplementary Table S5, available as Supplementary data at *IJE* online). There was little between-cohort variation except for the 2011/12 cohort, whose cumulative incidence by age 8 was slightly lower.

**Figure 2.**
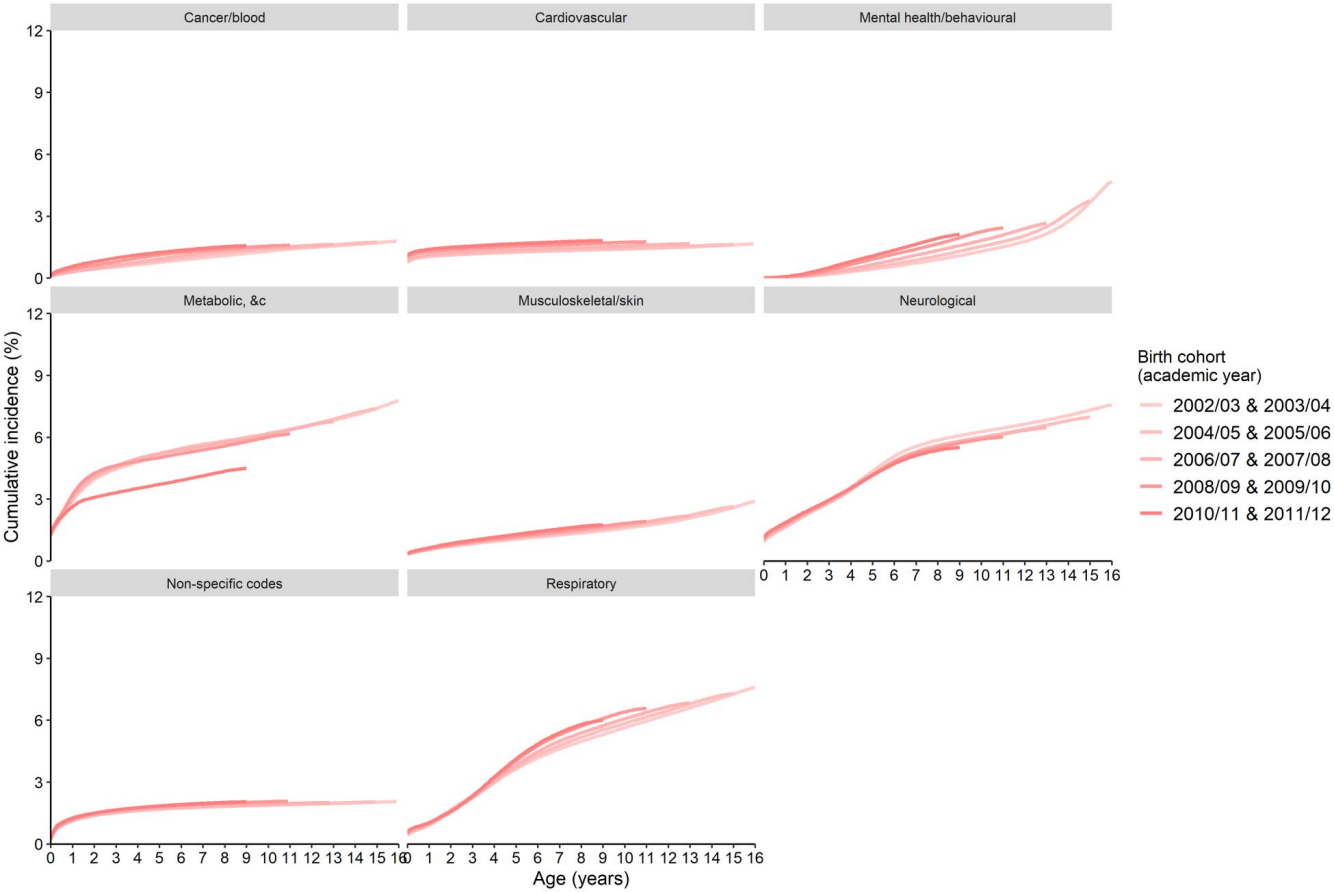
Cumulative incidence of being admitted to hospital and having any record indicating each subtype of chronic health condition before age 16 (closed birth cohorts). The metabolic and other group includes metabolic, endocrine, digestive, renal and genitourinary conditions. Academic year = 1 September to 31 August. See Supplementary Table S1 (available as Supplementary data at *IJE* online) for a full list of codes by each group. Underlying percentages are available in Supplementary Table S5 (available as Supplementary data at *IJE* online)

The subtypes with the highest cumulative incidence by age 16 were metabolic and others (including metabolic, endocrine, digestive, renal and genitourinary conditions), neurological and respiratory conditions (Figure 2;Supplementary Table S5, available as Supplementary data at *IJE* online). There was little temporal variation for cancer/blood, cardiovascular, musculoskeletal/skin conditions and non-specific codes. The cumulative incidence of mental health/behavioural codes increased over time and the metabolic and other conditions decreased in the 2010/11 and 2011/12 birth cohorts. The most common codes in each subtype in the 2002/3 and 2003/4 cohorts are presented in Supplementary Table S6 (available as Supplementary data at *IJE* online).

### Sensitivity analyses

The cumulative incidence of any CHC in the 2003/4 cohort ranged from 22.0% under the first sensitivity analysis (requiring only a birth record in HES) to 31.7% in the sixth and seventh (requiring a birth record in HES as well as HES activity to age 5, additional HES activity ages 5 to 16 and linkage to NPD): Supplementary Figure S3, Supplementary Table S7 (available as Supplementary data at *IJE* online) which also contains data for other cohorts. The cumulative incidences of subtypes varied similarly (Supplementary Figure S4 and Supplementary Table S7, available as Supplementary data at *IJE* online).

### Multimorbidity

Substantial proportions of children had more than one body system affected (Supplementary Figure S5, Supplementary Table S8, available as Supplementary data at *IJE* online). For example, in the 2003/4 cohort, of children with at least one subtype of CHC ever recorded, 21% had codes indicating more than one subtype by age 5, 24% by age 11 and 28% by age 16. Multimorbidity rose in later cohorts: in the 2011/12 cohort, 25% of children with any CHC had more than one subtype recorded by age 5.

### Cumulative incidence: Method 2 (open cohorts)

**Figure 3.**
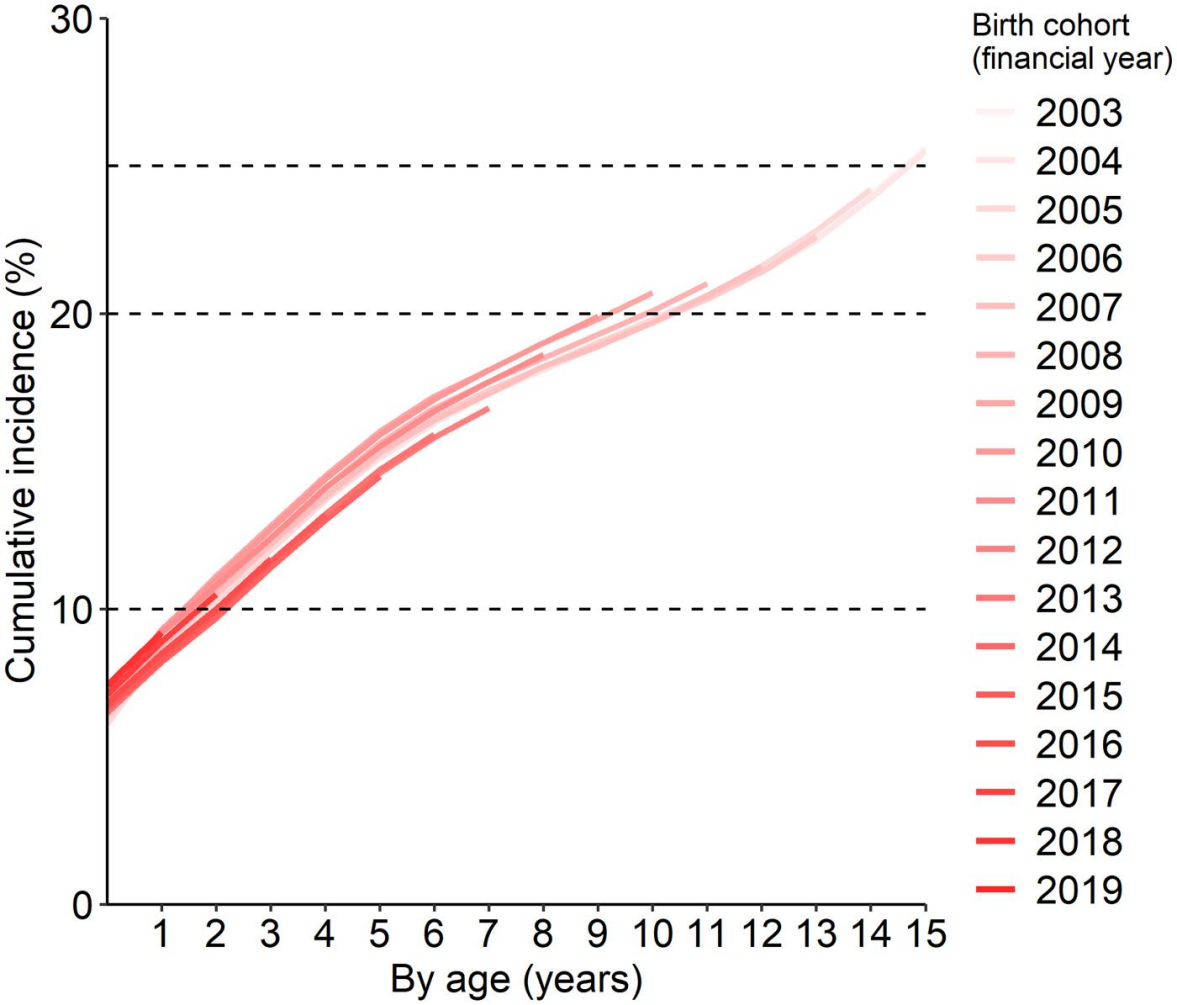
Cumulative incidence of being admitted to hospital and having any record indicating a chronic health condition before age 16 (open birth cohorts). Horizontal guidelines are at 10%, 20% and 25%. Financial year = 1 April to 31 March. Underlying percentages are available in Supplementary Table S9 (available as Supplementary data at *IJE* online)

Figure 3 (Supplementary Table S9, available as Supplementary data at *IJE* online) shows the cumulative incidence of a hospital admission with any CHC code, using the open cohort approach, was 26% of children in the 2003 and 2004 cohorts, similar to the closed cohort approach. Results were also similar between the two methods when examining CHC subtypes (Supplementary Figure S6, Supplementary Table S8, available as Supplementary data at *IJE* online).

## Discussion

Using open and closed cohorts based on HES, we estimated that a quarter of children born in England in 2002/3 had a hospital-recorded CHC at least once before turning 16. In closed birth cohorts, this estimate ranged from 22% to 32% in sensitivity analyses, with different eligibility criteria based on greater or lesser requirements of HES and/or NPD activity following birth. Findings were similar for later cohorts. Given we used admissions, where thresholds for intervention are higher than in outpatients or primary care, ours are lower-bound estimates for the cumulative incidence of all CHCs in the community. We also observed substantial multimorbidity. Where children in the 2003/4 cohort had a CHC code, 28% of them had more than one body system affected from birth to age 16. Our method of analysing multimorbidity (counting body systems affected) underestimates the true extent of multimorbidity, as it is possible that children may have multiple conditions within each subtype.

Our findings, therefore, describe a population significantly affected by CHCs across childhood in terms of incidence and complexity. This highlights the scale of the challenges that health care, education, social care and other services face in supporting young people.

The decrease in the cumulative incidence of admissions with a CHC diagnosis to age 8 in later cohorts was explicable based on decreases in coding of the metabolic and other conditions, in particular in code K52.9 (non-infective gastroenteritis and colitis; results not shown). This unexplained observation may reflect changes in coding practice, particularly regarding rotavirus and infective gastroenteritis/colitis.^15^

We observed increases in mental health/behavioural conditions with more recent cohorts. This may reflect better detection and diagnosis of mental health conditions, better recording in data and/or changes in the underlying incidence. An increase in admissions with mental health CHCs is consistent with documented increasing rates of already highly prevalent mental health problems among children between 1999 and 2023 in surveys.^16–20^ There have also been increases in psychiatric admission rates that outstrip increases in rates of mental health problems in the community, suggesting a need for greater focus on prevention and early help.^21^

Prevalence estimates of mental health problems in the UK have used varied definitions, ranging from 10% for diagnosed disorders according to the Diagnostic and Statistical Manual of Mental Disorders (DSM-IV)^18^ to 46% experiencing at least some instances of mental health problems through the period of childhood and adolescence,^19^ with others inbetween.^17^^,^^22^ These estimates are far higher than our cumulative estimate of 4.7% to age 16 among children in the 2002/3 and 2003/4 cohorts, undoubtedly because we used hospital admission data which, although they do include psychiatric admissions in acute settings, do not include primary care or mental health clinic contacts or admissions to specialist mental health units. In future, linkage to the community Mental Health Services Data Set^23^ may provide more comprehensive estimates, which could be further enhanced if whole-population primary care data were available, as in Wales.^22^

### Comparison with other cumulative estimates

Our definition of CHCs was created and validated for use with hospital data. CHCs were defined as any condition where follow-up of at least a year is likely.^14^ Alternative definitions, however, are available, including self-reported.^24^Table 4 provides an overview of previous cumulative incidence estimates, which vary from 11% to 52%. That there are few studies is perhaps a symptom of the fact that deriving a generic definition of CHCs, ascertainable with accuracy, is difficult,^24^ particularly when relying on self-report over time. In recent years, sufficient administrative data have been available, enabling the present study. Unfortunately, in-depth comparisons are difficult owing to methodological differences, including parent-report vs ascertainment in health records. However, our estimate of a quarter is of a similar order of magnitude as these others.

**Table 4.** Summary of estimates of the cumulative incidence of chronic health conditions in childhood

Study	Data source	Case ascertainment	Estimate
This study (closed birth cohorts)	Hospital Episode Statistics linked to National Pupil Database	Codes in hospital admission records: ‘any health problem likely to require follow-up for more than 1 year, where follow-up could be repeated hospital admission, specialist follow-up through outpatient department visits, medication or use of support services’.^14^	24.5% to age 16 years
This study (open cohorts)	Hospital Episode Statistics; Office for National Statistics population denominators	As above.	25.5% to age 16 years
Pless *et al.*, 1993^36^	1958 National Child Development Study (national birth cohort in Great Britain)	Parent-reported chronic disorder: ‘a physical, usually non-fatal condition lasting longer than 3 months’.	11% to age 16 years
Van Cleave *et al*., 2010^37^	US National Longitudinal Survey of Labor Market Experience, Youth Cohort (three cohorts aged 2 to 6, followed up to 8 to 14 years)	Maternal-reported physical, emotional or mental conditions (at least 12 months in duration, interfering with daily activities or requiring treatment)	Across 6 years (up to ages 8 to 14 years): Cohort 1, 1988 to 1994 = 27% Cohort 2, 1994 to 2000 = 41% Cohort 3, 2000 to 2006 = 52%

### Strengths and limitations

Strengths include both open and closed cohort approaches, extensive sensitivity analyses and our whole-country approach, which uses routinely collected data from universal, free at the point of use, services. HES and NPD data are collected according to national standards and undergo routine validation and cleaning before release to researchers.^10^^,^^25^ We used a generic definition of CHCs, widening the applicability of our results to health, social care and education services and policy makers.^26^ The consistency over years observed in this study using the Hardelid *et al.*^14^ code list suggests that it, and the nine body systems studied, may be suitable for monitoring long-term trends, particularly as the data are widely available and regularly used by government, academia and other stakeholders.

A primary limitation is that our use of hospital inpatient data will have led to under-ascertainment of some CHCs, particularly mental health conditions and those seen in primary care or community health contexts where admissions are not required. Primary care and prescribing data, as well as diagnostic coding in hospital outpatients and Accident and Emergency attendances, were not available. Where CHCs appear in HES inpatient data, they are likely to reflect more severe presentations than those managed in community settings. Our estimates are therefore lower bounds for the true cumulative incidence of all CHCs. Additionally, we were only able to examine the first recorded instance of a CHC code. It was impossible for us to determine disease severity or whether and when children recovered. This means we were unable to estimate disease burden, for example, in terms of actual functional deficits following diagnosis, treatment or recovery.

### Policy implications

Our findings that a quarter of children in England were admitted to hospital with a CHC before age 16 demonstrate the extent to which universal health, social care and education services are in contact with children who potentially have additional needs. According to our estimates, of the ∼600 000 children born each year in England, 84 000 will have had a CHC before starting primary school aged 5, 114 000 before secondary school aged 11 and 144 000 before finishing compulsory education aged 16. There is a significant degree of commonality between different conditions in terms of educational barriers and impairments that require adjustments to access learning effectively, including provision for support for special educational needs, involvement of social care and health care, particularly primary care, community disability teams and health visiting.^26^

### Research implications

Method 1 (closed birth cohorts) does not account for immigration, nor fully for emigration, so we also estimated the cumulative incidence of CHCs using open cohorts (Method 2), which resulted in similar estimates. Our findings are relevant to future studies using birth cohorts based on HES and ECHILD and suggest that biases induced by migration can be addressed using linkage to later HES and/or NPD records (whether this is true for all outcomes is an open question). To enhance the accuracy of denominators, a whole population denominator spine, updated continuously, is necessary. Recent developments in administration-based population estimates (estimates based directly on linked, administrative data) and statistical population datasets (modelled datasets) are leading to the development by ONS of dynamic population models.^27^^,^^28^ Having a near real-time population estimate, disaggregated at least by age for denominators, combined with existing health data such as HES for numerators, will provide enhanced cumulative incidence estimates in the future. This is being achieved using a combination of administrative data sources including the Personal Demographic Service (containing the NHS number and demographic information on all patients registered with general practitioners or using NHS services).^29^

### Conclusion

We estimated that at least one in four children in England has a CHC before turning 16, reflecting a high burden on children and families, schools, health and social care services. Incidence estimates were sensitive to research design including cohort specification (open or closed membership) and eligibility criteria.

## Ethics approval

Permissions to use linked, de-identified data from Hospital Episode Statistics and the National Public Database were granted by DfE (DR200604.02B) and NHS England (DARS-NIC-381972). Ethical approval for the ECHILD project was granted by the National Research Ethics Service (17/LO/1494), NHS Health Research Authority Research Ethics Committee (20/EE/0180 and 21/SW/0159) and is overseen by the UCL Great Ormond Street Institute of Child Health’s Joint Research and Development Office (20PE16).

## Supplementary Material

dyae138_Supplementary_Data

## Data Availability

Data were shared by NHS England and the Department for Education under licence. Access can be applied for through the ECHILD team via the website: [https://www.echild.ac.uk/]. R code generated for this study can be found on the UCL Child Health Informatics Group GitHub page [https://github.com/UCL-CHIG/chronic-health-conditions-incidence].
