# Supplementary material for: Cumulative incidence of chronic health conditions recorded in hospital inpatient admissions from birth to age 16 in England
Source: Int J Epidemiol. 2024 Oct 10;53(5):dyae138. doi: 10.1093/ije/dyae138 (PMC11466227; doi:10.1093/ije/dyae138)
Supplement: dyae138_Supplementary_Data [file dyae138_supplementary_data.zip › ije-2024-01-0060-File008.docx]

**Supplementary Figures**

Contents

[Figure S1. Kaplan-Meier plots showing time to first recorded activity in HES or NPD in each birth cohort 2](#_Toc171944919)

[Figure S2. Kaplan-Meier plots showing time to first recorded activity in each HES module in each birth cohort 3](#_Toc171944920)

[Figure S3. Cumulative incidence of being admitted to hospital and having any record indicating a chronic health condition before age 16 in the main analysis and sensitivity analyses (2002/3 & 2003/4 birth cohorts) 4](#_Toc171944921)

[Figure S4. Cumulative incidence of being admitted to hospital and having any record indicating each sub-type of chronic health condition before age 16 in the main analysis and sensitivity analyses (2002/3 & 2003/4 birth cohorts) 5](#_Toc171944922)

[Figure S5. Proportions of children with two or more chronic health condition sub-types in each birth cohort (restricted to children in each cohort with at least one sub-type recorded) 6](#_Toc171944923)

[Figure S6. Cumulative incidence of being admitted to hospital and having any record indicating each sub-type of chronic health condition before age 16 (open cohorts) 7](#_Toc171944924)

# Figure S1. Kaplan-Meier plots showing time to first recorded activity in Hospital Episode Statistics or National Pupil Database in each birth cohort


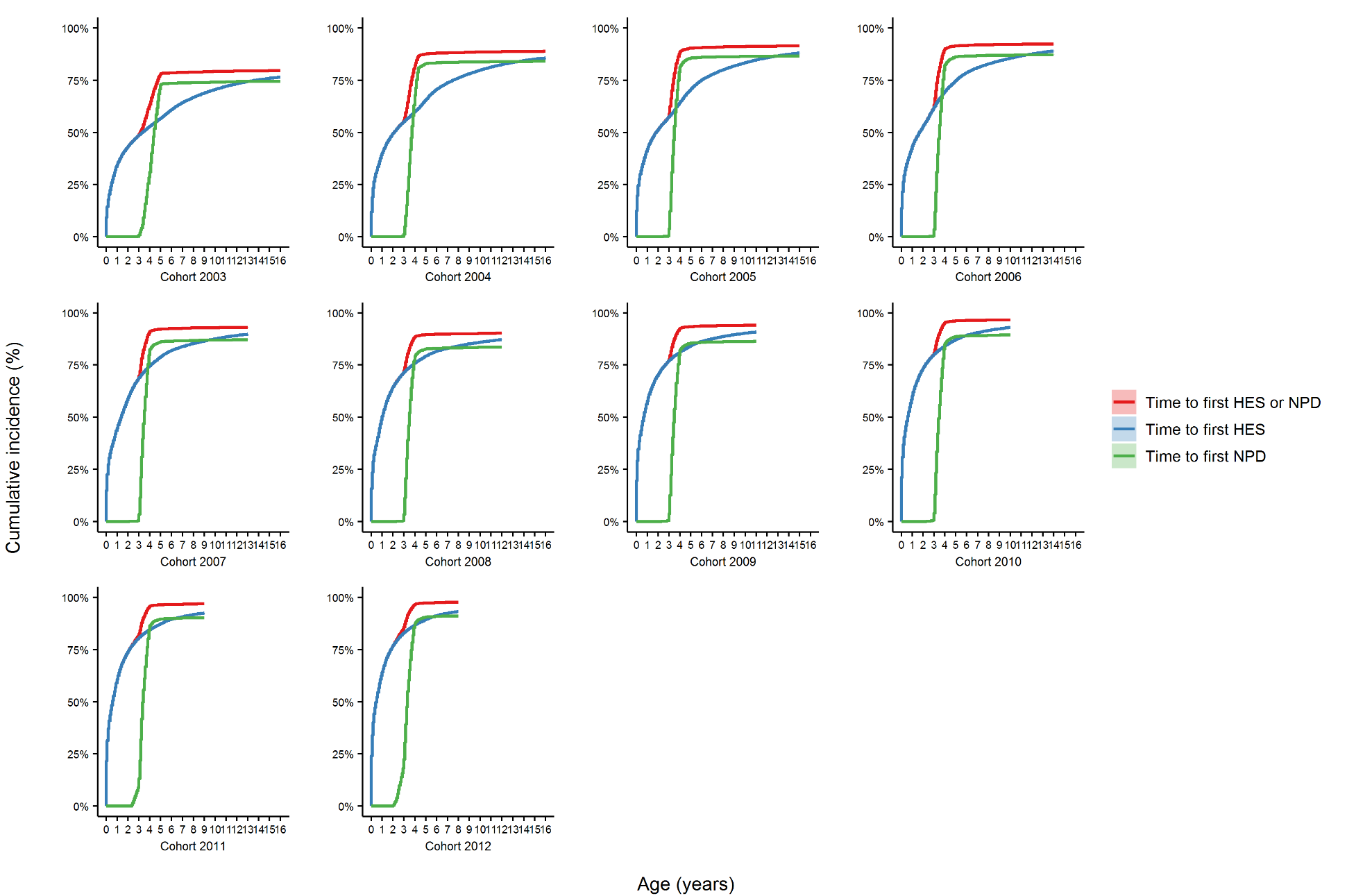


HES Hospital Episode Statistics; NPD National Pupil Database

# Figure S2. Kaplan-Meier plots showing time to first recorded activity in each Hospital Episode Statistics module in each birth cohort


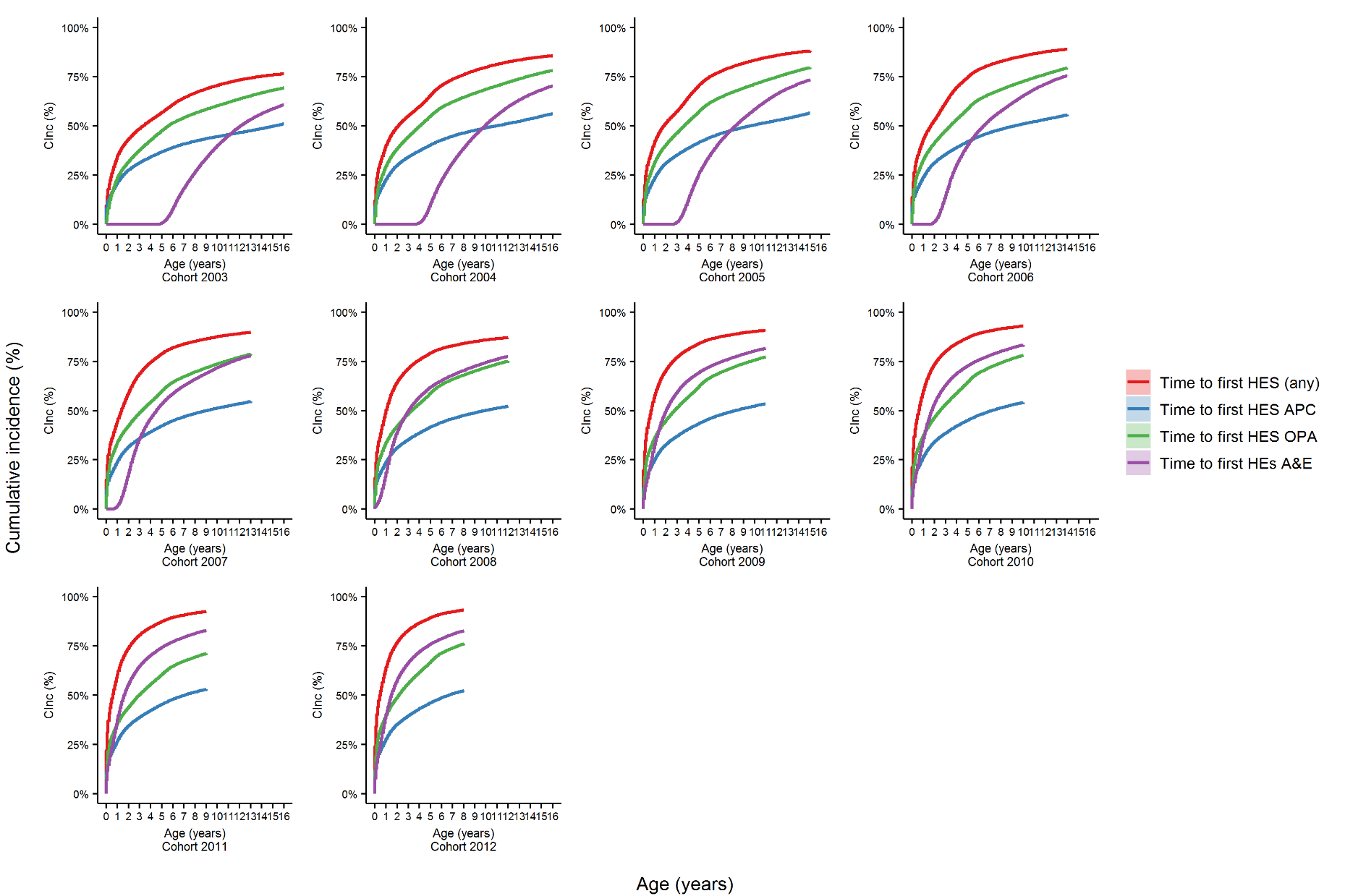


A&E Accident & Emergency; APC Admitted Patient Care; HES Hospital Episode Statistics; NPD National Pupil Database; OPA outpatients

# Figure S3. Cumulative incidence of being admitted to hospital and having any record indicating a chronic health condition before age 16 in the main analysis and sensitivity analyses (2002/3 & 2003/4 birth cohorts)


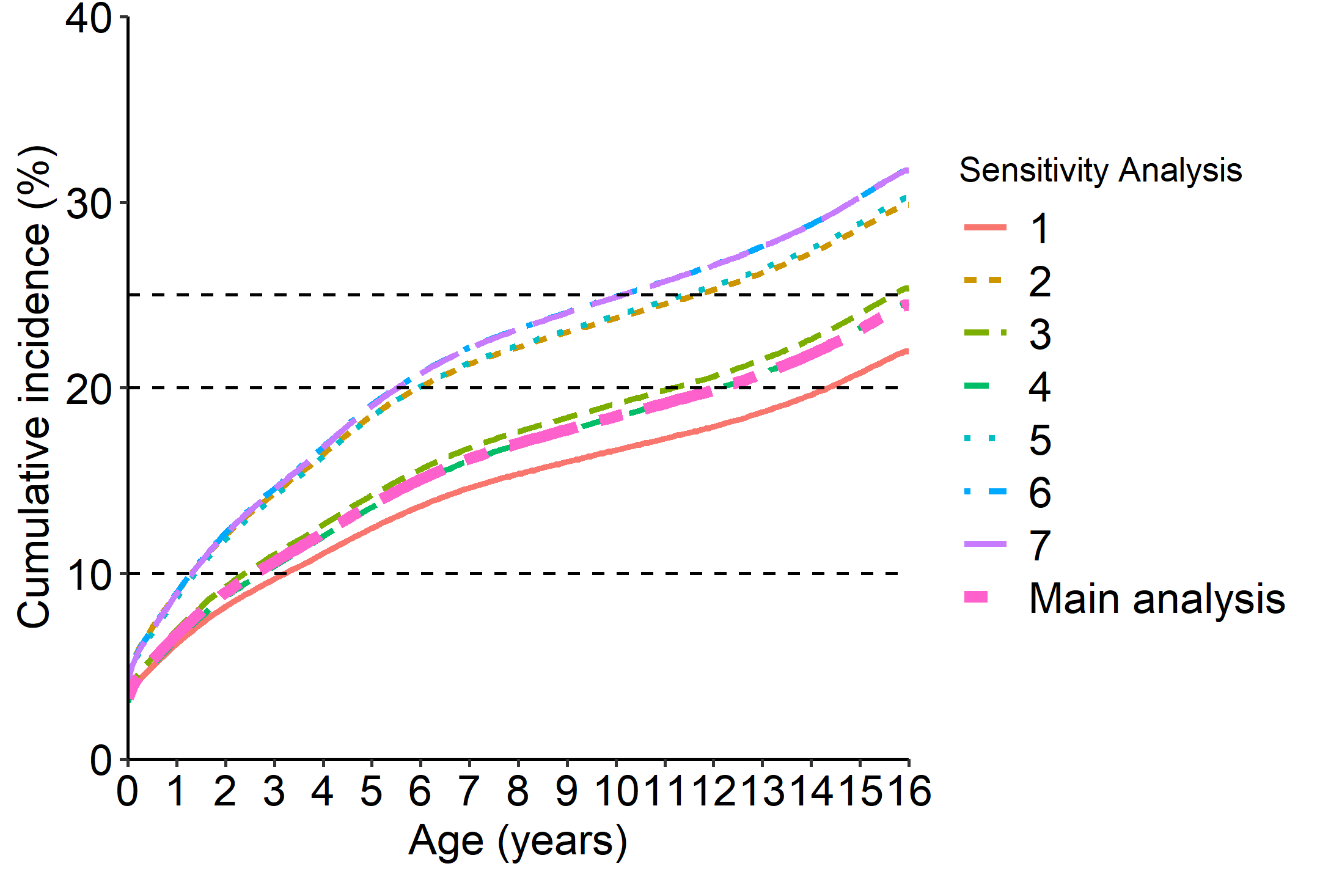


Horizontal guide lines are at 10%, 20% and 25%. The thickest pink, dashed line is the main analysis. Underlying percentages are availabine in Supplementary Tables S5 (main analysis) and S7 (sensitivity analyses, which also contains data for all other cohorts). See main text and Table 2 for details of each sensitivity analysis

# Figure S4. Cumulative incidence of being admitted to hospital and having any record indicating each sub-type of chronic health condition before age 16 in the main analysis and sensitivity analyses (2002/3 & 2003/4 birth cohorts)


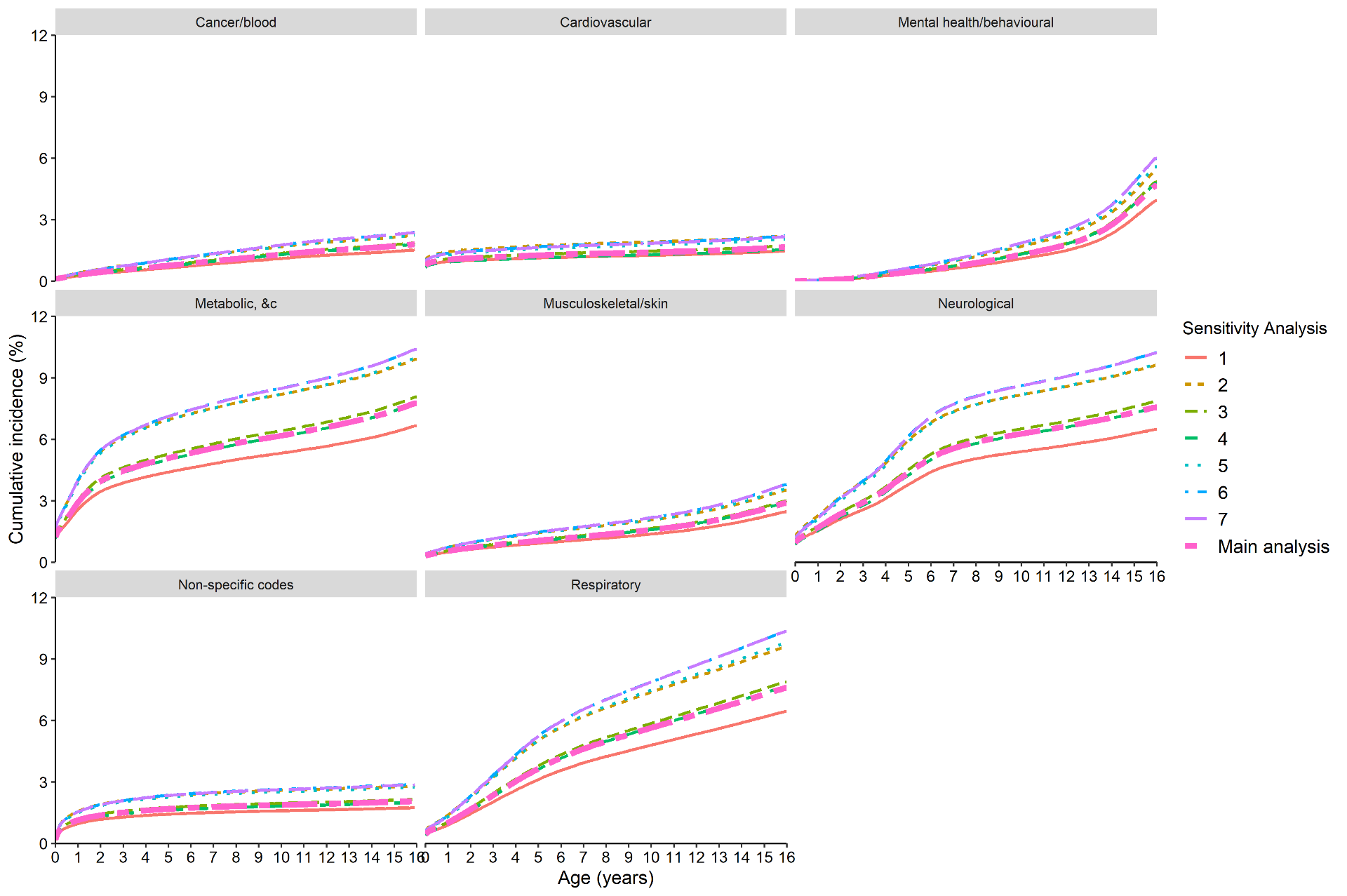


The Metabolic, &c group includes metabolic, endocrine, digestive, renal and genitourinary conditions. See Supplementary Table S1 for a full list of codes by each group. Underlying percentages are available in Supplementary Table S5 (main analysis) and S7 (sensitivity analyses), which also includes data for all other cohorts. See main text and Table 2 for details of each sensitivity analysis

# Figure S5. Proportions of children with two or more chronic health condition sub-types in each birth cohort (restricted to children in each cohort with at least one sub-type recorded)


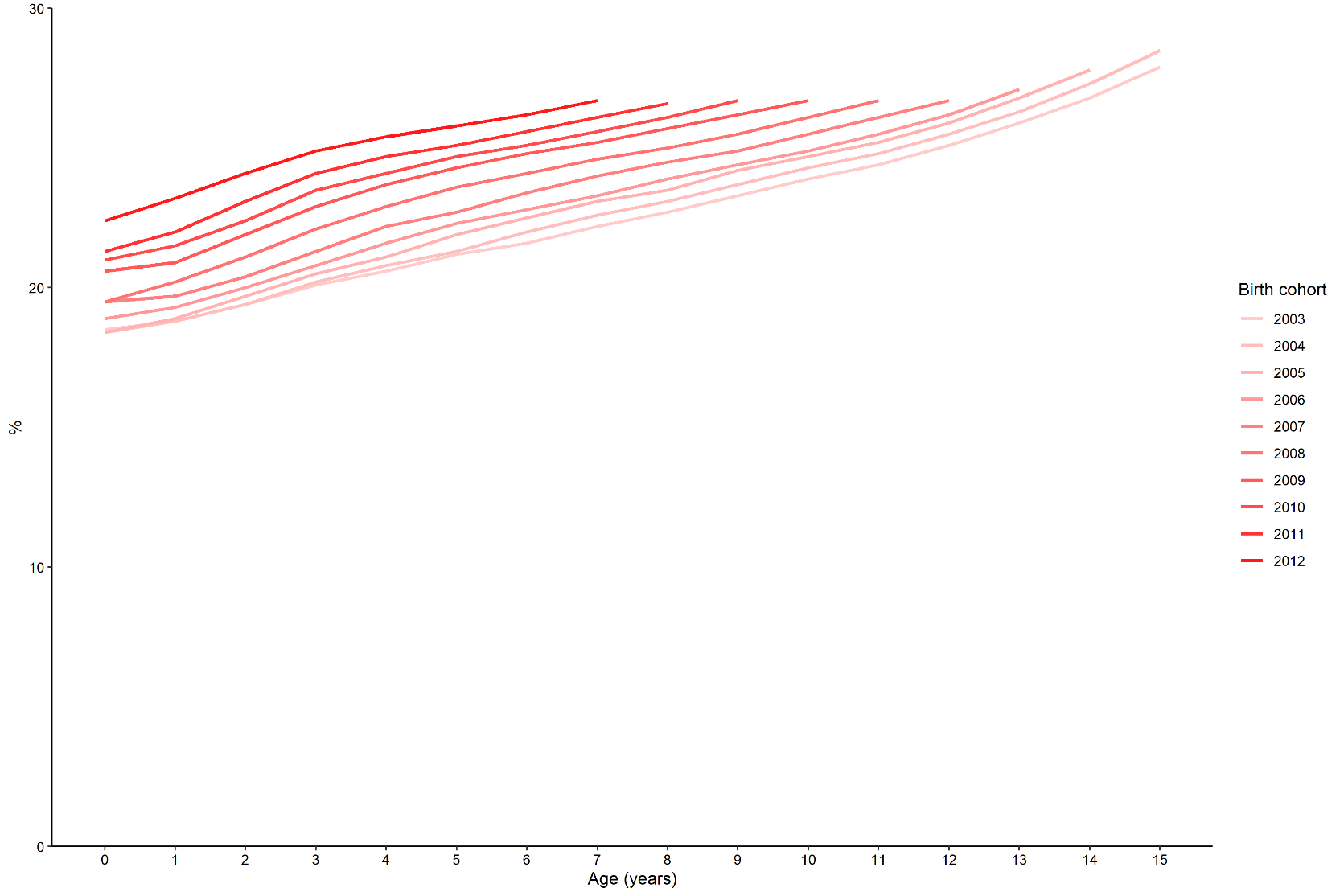


Underlying percentages are available in Supplementary Table S8.

# Figure S6. Cumulative incidence of being admitted to hospital and having any record indicating each sub-type of chronic health condition before age 16 (open cohorts)


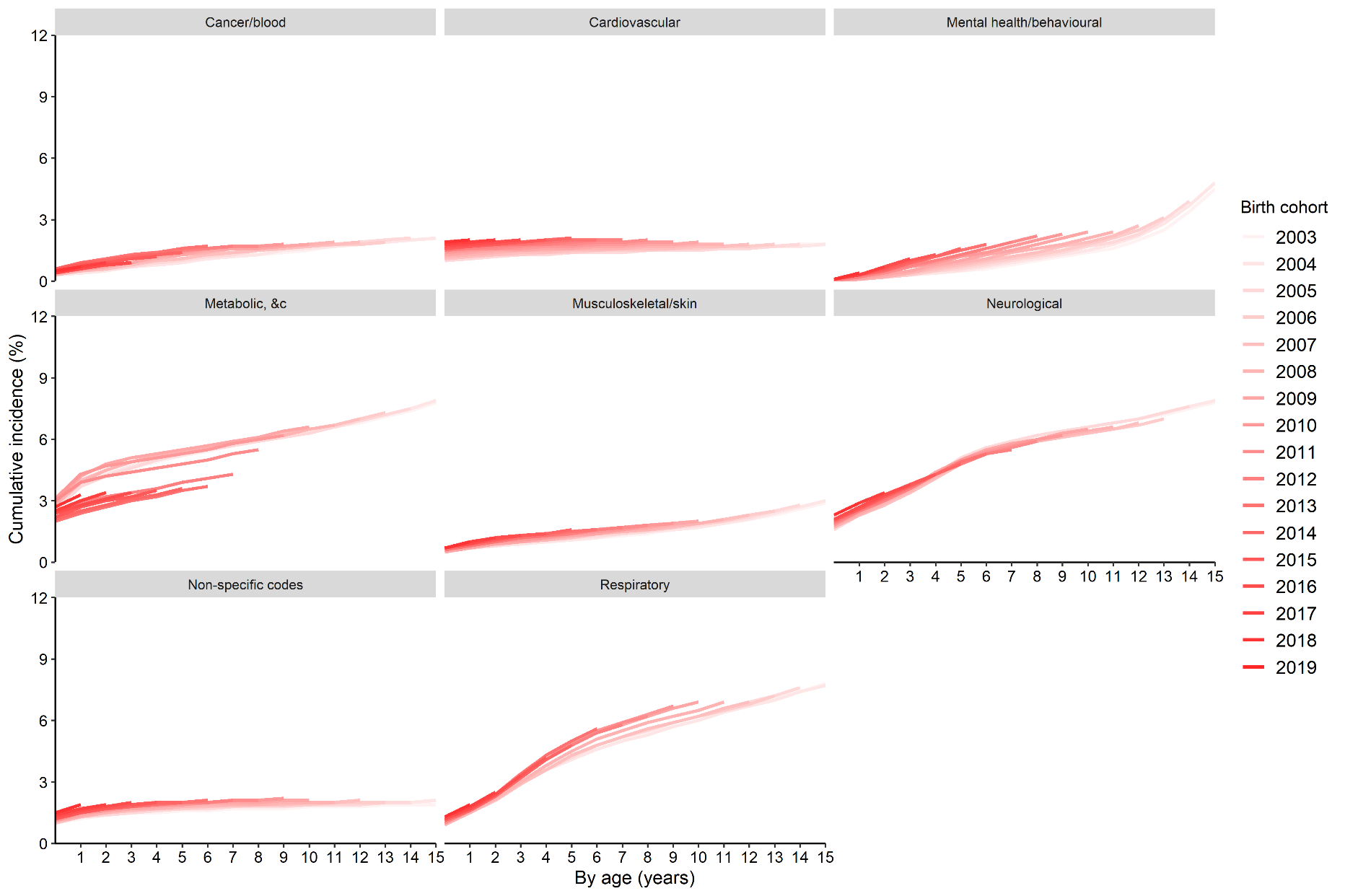


Underlying percentages are available in Supplementary Table S9.
