# Supplementary material for: Cumulative incidence of chronic health conditions recorded in hospital inpatient admissions from birth to age 16 in England
Source: Int J Epidemiol. 2024 Oct 10;53(5):dyae138. doi: 10.1093/ije/dyae138 (PMC11466227; doi:10.1093/ije/dyae138)
Supplement: dyae138_Supplementary_Data [file dyae138_supplementary_data.zip › ije-2024-01-0060-File007.docx]

**Supplementary Tables S2-S9**

**Contents**

[Table S2. R packages used 2](#_Toc171945121)

[Table S3. Cohort demographic and clinical characteristics of each cohort 3](#_Toc171945122)

[Table S4. Cohort demographic and clinical characteristics in each sensitivity analysis 5](#_Toc171945123)

[Table S5. Cumulative incidence of being admitted to hospital and having any record indicating a chronic health condition before age 16 in the main analysis (underlying data for Figures 1 and 2) 7](#_Toc171945124)

[Table S6. The twenty most frequent ICD-10 diagnostic codes detected in each sub-type in the 2002/3 and 2003/4 cohorts grouped by age of first detection (birth to age 4, age 5 to 10 and age 11 to 15); codes are only counted once per child, rather than once per admission 10](#_Toc171945125)

[Table S7. Cumulative incidence of being admitted to hospital and having any record indicating any CHC and each sub-type before age 16 in the sensitivity analyses 22](#_Toc171945126)

[Table S8. Proportions of children with two or more chronic health condition sub-types in each birth cohort (restricted to children in each closed cohort with at least one sub-type recorded; underlying data for Figure 3). 39](#_Toc171945127)

[Table S9. Cumulative incidence of being admitted to hospital and having any record indicating a chronic health condition before age 16 (closed cohorts, underlying data for Figure 3 and Supplementary Figure S6) 40](#_Toc171945128)

# Table S2. R packages used

| Package | CRAN link (all last accessed 13 November 2023) |
| --- | --- |
| data.table | <https://cran.r-project.org/web/packages/data.table/index.html> |
| ggplot2 | <https://cran.r-project.org/web/packages/ggplot2/index.html> |
| ggpubr | <https://cran.r-project.org/web/packages/ggpubr/index.html> |
| ggsurvfit | <https://cran.r-project.org/web/packages/ggsurvfit/index.html> |
| gridExtra | <https://cran.r-project.org/web/packages/gridExtra/index.html> |
| lubridate | <https://cran.r-project.org/web/packages/lubridate/index.html> |
| RODBC | <https://cran.r-project.org/web/packages/RODBC/index.html> |
| scales | <https://cran.r-project.org/web/packages/scales/index.html> |
| survival | <https://cran.r-project.org/web/packages/survival/index.html> |
| survminer | <https://cran.r-project.org/web/packages/survminer/index.html> |
| tidycmprsk | <https://cran.r-project.org/web/packages/tidycmprsk/index.html> |

CRAN Comprehensive R Archive Network.

# Table S3. Cohort demographic and clinical characteristics of each cohort

|  |  | 2002/03 | 2003/04 | 2004/05 | 2005/06 | 2006/07 | 2007/08 | 2008/09 | 2009/10 | 2010/11 | 2011/12 |
| --- | --- | --- | --- | --- | --- | --- | --- | --- | --- | --- | --- |
| Female |  | 260036 (48.8%) | 267393 (48.8%) | 272678 (48.9%) | 281886 (48.8%) | 290051 (48.6%) | 303470 (48.7%) | 308592 (48.7%) | 316087 (48.8%) | 316212 (48.7%) | 308393 (48.7%) |
| Ethnicity | White | 314959 (80.8%) | 343586 (80%) | 365307 (79.4%) | 381556 (77.5%) | 401049 (76.4%) | 422461 (76.2%) | 436438 (75.6%) | 458052 (75.7%) | 465125 (75.7%) | 453053 (75.7%) |
|  | Black | 18051 (4.6%) | 21877 (5.1%) | 24587 (5.3%) | 28366 (5.8%) | 30804 (5.9%) | 32381 (5.8%) | 32978 (5.7%) | 34419 (5.7%) | 34030 (5.5%) | 30352 (5.1%) |
|  | Mixed | 10718 (2.8%) | 12607 (2.9%) | 14963 (3.3%) | 16584 (3.4%) | 19150 (3.6%) | 20646 (3.7%) | 23726 (4.1%) | 26477 (4.4%) | 27032 (4.4%) | 27876 (4.7%) |
|  | Asian | 35442 (9.1%) | 39114 (9.1%) | 42104 (9.1%) | 47503 (9.7%) | 52971 (10.1%) | 56705 (10.2%) | 62076 (10.8%) | 64569 (10.7%) | 66993 (10.9%) | 67043 (11.2%) |
|  | Other | 10460 (2.7%) | 12234 (2.8%) | 13329 (2.9%) | 18033 (3.7%) | 20940 (4%) | 22313 (4%) | 21974 (3.8%) | 21406 (3.5%) | 21396 (3.5%) | 20436 (3.4%) |
|  | Missing | 143776 (-%) | 118218 (-%) | 97637 (-%) | 86404 (-%) | 75681 (-%) | 70427 (-%) | 56382 (-%) | 43448 (-%) | 35060 (-%) | 34910 (-%) |
| IMD | 1 (most deprived) | 146640 (27.5%) | 149219 (27.3%) | 152378 (27.3%) | 162890 (28.2%) | 167558 (27.9%) | 172933 (28.1%) | 172603 (28%) | 175864 (27.9%) | 178545 (27.9%) | 176537 (27.9%) |
|  | 2 | 110878 (20.8%) | 115226 (21.1%) | 118755 (21.3%) | 123595 (21.4%) | 129287 (21.6%) | 134109 (21.8%) | 136812 (22.2%) | 140601 (22.3%) | 144298 (22.6%) | 144050 (22.7%) |
|  | 3 | 95657 (17.9%) | 98842 (18.1%) | 101379 (18.2%) | 103973 (18%) | 107668 (17.9%) | 110412 (18%) | 112715 (18.3%) | 116156 (18.5%) | 117969 (18.4%) | 117558 (18.6%) |
|  | 4 | 89973 (16.9%) | 92066 (16.8%) | 92985 (16.7%) | 94499 (16.3%) | 97934 (16.3%) | 99228 (16.1%) | 99368 (16.1%) | 100870 (16%) | 101999 (15.9%) | 101296 (16%) |
|  | 5 (lest deprived) | 89987 (16.9%) | 91860 (16.8%) | 91884 (16.5%) | 93110 (16.1%) | 97411 (16.2%) | 97811 (15.9%) | 95264 (15.4%) | 96050 (15.3%) | 96708 (15.1%) | 94024 (14.8%) |
|  | Missing | 271 (-%) | 423 (-%) | 546 (-%) | 379 (-%) | 737 (-%) | 10440 (-%) | 16812 (-%) | 18830 (-%) | 10117 (-%) | 205 (-%) |
| Region | NE | 25637 (4.8%) | 24239 (4.4%) | 26396 (4.7%) | 27930 (4.8%) | 28651 (4.8%) | 29352 (4.7%) | 29314 (4.6%) | 30155 (4.7%) | 30007 (4.6%) | 28969 (4.6%) |
|  | NW | 72311 (13.6%) | 73695 (13.5%) | 75290 (13.5%) | 77871 (13.5%) | 78922 (13.1%) | 86391 (13.8%) | 84621 (13.4%) | 84886 (13.1%) | 85251 (13.1%) | 83152 (13.1%) |
|  | Yorks & Hum | 53150 (10%) | 55041 (10.1%) | 55870 (10%) | 58319 (10.1%) | 60578 (10.1%) | 63168 (10.1%) | 63737 (10.1%) | 64753 (10%) | 62759 (9.7%) | 62262 (9.8%) |
|  | E Mid | 43500 (8.2%) | 44503 (8.1%) | 45153 (8.1%) | 47401 (8.2%) | 49414 (8.2%) | 51820 (8.3%) | 51349 (8.1%) | 52152 (8%) | 52414 (8.1%) | 50984 (8%) |
|  | W Mid | 59256 (11.1%) | 60746 (11.1%) | 61584 (11%) | 62560 (10.8%) | 61486 (10.2%) | 63077 (10.1%) | 66373 (10.5%) | 69814 (10.8%) | 70571 (10.9%) | 68529 (10.8%) |
|  | E Eng | 54672 (10.2%) | 57227 (10.4%) | 57752 (10.4%) | 59219 (10.2%) | 63387 (10.6%) | 65843 (10.5%) | 66048 (10.4%) | 66642 (10.3%) | 67767 (10.4%) | 67251 (10.6%) |
|  | Lon | 97543 (18.3%) | 101114 (18.5%) | 100374 (18%) | 107238 (18.5%) | 114301 (19%) | 117133 (18.7%) | 119261 (18.8%) | 124723 (19.2%) | 123485 (19%) | 120108 (19%) |
|  | SE | 82219 (15.4%) | 84379 (15.4%) | 86016 (15.4%) | 88471 (15.3%) | 91986 (15.3%) | 94929 (15.2%) | 98507 (15.5%) | 100701 (15.5%) | 102280 (15.7%) | 96119 (15.2%) |
|  | SW | 45118 (8.5%) | 46692 (8.5%) | 49492 (8.9%) | 49437 (8.5%) | 51870 (8.6%) | 53220 (8.5%) | 54364 (8.6%) | 54545 (8.4%) | 55102 (8.5%) | 56296 (8.9%) |
| Low birth | Yes | 24752 (6.2%) | 26940 (6.3%) | 26288 (6.1%) | 27987 (6.3%) | 29073 (6.5%) | 29526 (6.1%) | 36551 (6.5%) | 37955 (6.4%) | 35914 (6.1%) | 32404 (5.6%) |
| weight | Missing | 132099 (-%) | 118523 (-%) | 125585 (-%) | 133793 (-%) | 156610 (-%) | 143316 (-%) | 75071 (-%) | 59778 (-%) | 57436 (-%) | 51982 (-%) |
| Birth | Normal | 376555 (93.8%) | 402173 (93.7%) | 406054 (93.9%) | 416666 (93.7%) | 414912 (93.5%) | 452091 (93.9%) | 521952 (93.5%) | 550638 (93.6%) | 556286 (93.9%) | 549284 (94.4%) |
| weight | Low | 21165 (5.3%) | 22970 (5.4%) | 22462 (5.2%) | 23938 (5.4%) | 24793 (5.6%) | 25264 (5.2%) | 31019 (5.6%) | 32298 (5.5%) | 30972 (5.2%) | 27863 (4.8%) |
| category | Very Low | 2236 (0.6%) | 2400 (0.6%) | 2421 (0.6%) | 2514 (0.6%) | 2547 (0.6%) | 2601 (0.5%) | 3364 (0.6%) | 3522 (0.6%) | 3103 (0.5%) | 2885 (0.5%) |
|  | Extr low | 1351 (0.3%) | 1570 (0.4%) | 1405 (0.3%) | 1535 (0.3%) | 1733 (0.4%) | 1661 (0.3%) | 2168 (0.4%) | 2135 (0.4%) | 1839 (0.3%) | 1656 (0.3%) |
|  | Missing | 132099 (-%) | 118523 (-%) | 125585 (-%) | 133793 (-%) | 156610 (-%) | 143316 (-%) | 75071 (-%) | 59778 (-%) | 57436 (-%) | 51982 (-%) |
| Premature | Yes | 23197 (6.2%) | 24759 (6.5%) | 23565 (6.2%) | 23464 (6.4%) | 24606 (6.7%) | 28987 (6.6%) | 36346 (6.8%) | 39107 (6.7%) | 36303 (6.2%) | 33156 (5.7%) |
|  | Missing | 161731 (-%) | 165423 (-%) | 176409 (-%) | 212974 (-%) | 233755 (-%) | 183349 (-%) | 99347 (-%) | 63208 (-%) | 62384 (-%) | 52569 (-%) |
| Gest. age | Term | 348478 (93.8%) | 357454 (93.5%) | 357953 (93.8%) | 342008 (93.6%) | 342234 (93.3%) | 412597 (93.4%) | 497881 (93.2%) | 546056 (93.3%) | 550949 (93.8%) | 547945 (94.3%) |
|  | Prem | 19576 (5.3%) | 20901 (5.5%) | 19995 (5.2%) | 19823 (5.4%) | 20564 (5.6%) | 24389 (5.5%) | 30339 (5.7%) | 32925 (5.6%) | 30762 (5.2%) | 28115 (4.8%) |
|  | Very prem | 2439 (0.7%) | 2596 (0.7%) | 2491 (0.7%) | 2456 (0.7%) | 2611 (0.7%) | 3014 (0.7%) | 3949 (0.7%) | 4032 (0.7%) | 3801 (0.6%) | 3419 (0.6%) |
|  | Extr prem | 1182 (0.3%) | 1262 (0.3%) | 1079 (0.3%) | 1185 (0.3%) | 1431 (0.4%) | 1584 (0.4%) | 2058 (0.4%) | 2150 (0.4%) | 1740 (0.3%) | 1622 (0.3%) |
|  | Missing | 161731 (-%) | 165423 (-%) | 176409 (-%) | 212974 (-%) | 233755 (-%) | 183349 (-%) | 99347 (-%) | 63208 (-%) | 62384 (-%) | 52569 (-%) |
| Mother | Yes | 37104 (7.3%) | 37561 (7.1%) | 38243 (7.1%) | 38977 (7%) | 38021 (6.6%) | 38763 (6.4%) | 38584 (6.3%) | 37150 (5.9%) | 33977 (5.3%) | 30635 (4.9%) |
| <20 yrs | Missing | 27174 (-%) | 20832 (-%) | 19603 (-%) | 20978 (-%) | 21512 (-%) | 23597 (-%) | 21068 (-%) | 17212 (-%) | 12394 (-%) | 9684 (-%) |
| Multiple birth |  | 6126 (1.1%) | 4851 (0.9%) | 5117 (0.9%) | 7419 (1.3%) | 13265 (2.2%) | 11930 (1.9%) | 16403 (2.6%) | 16918 (2.6%) | 11759 (1.8%) | 5006 (0.8%) |

Percentages are excluding children with missing values.

Extr: Extremely; Gest. age: Gestational age; IMD: Index of Multiple Deprivation fifths (1 = most deprived, 5 = least deprived); Prem: premature; Regions codes: NE North East; NW North West; Yorks & Hum Yorkshire & The Humber; E Mid East Midlands; W Mid West Midlands; E Eng East of England; Lon London; SE South East; SW South West.

Birthweight:

Normal > 2,500 g

Low > 1,500 g and < 2,500 g

Very low > 1,000 g and < 2,500 g

Extr low < 1,500 g

Gestational age:

Term > 37 weeks

Premture > 32 and < 37 weeks

Very prem > 28 and < 32 weeks

Extr prem < 28 weeks

# Table S4. Cohort demographic and clinical characteristics in each sensitivity analysis

|  |  | **Main analyis** |  | Sensitivity analyses | | | | | | |
| --- | --- | --- | --- | --- | --- | --- | --- | --- | --- | --- |
|  |  | **Birth & HES <16 or NPD** |  | 1. Birth only | 2. Birth & HES <5 | 3. Birth & HES <16 | 4. Birth & NPD | 5. Birth & HES <5 & NPD | 6. Birth & HES <5 & HES 5 to <16 | 7. Birth & HES <5 & HES 5 to <16 & NPD |
|  |  |  |  |  |  |  |  |  |  |  |
| *Birth cohorts* |  | *2003/4* |  | *2002/3 and 2003/4* | | | | | | |
|  |  |  |  |  |  |  |  |  |  |  |
| Female |  | **260036 (48.8%)** |  | 527651 (48.8%) | 304088 (46.2%) | 420104 (48%) | 414200 (48.5%) | 282274 (46.1%) | 268623 (45.6%) | 258556 (45.5%) |
| Ethnicity | White | **314959 (80.8%)** |  | 658545 (80.4%) | 442966 (80.6%) | 567170 (80.6%) | 551936 (80.9%) | 416978 (81%) | 405871 (80.9%) | 392241 (81.1%) |
|  | Black | **18051 (4.6%)** |  | 39928 (4.9%) | 25206 (4.6%) | 33774 (4.8%) | 32469 (4.8%) | 22979 (4.5%) | 22190 (4.4%) | 21116 (4.4%) |
|  | Mixed | **10718 (2.8%)** |  | 23325 (2.8%) | 15975 (2.9%) | 20191 (2.9%) | 19318 (2.8%) | 14764 (2.9%) | 14359 (2.9%) | 13748 (2.8%) |
|  | Asian | **35442 (9.1%)** |  | 74556 (9.1%) | 50921 (9.3%) | 63744 (9.1%) | 60837 (8.9%) | 47072 (9.1%) | 46497 (9.3%) | 44192 (9.1%) |
|  | Other | **10460 (2.7%)** |  | 22694 (2.8%) | 14829 (2.7%) | 19092 (2.7%) | 17817 (2.6%) | 13156 (2.6%) | 12737 (2.5%) | 12093 (2.5%) |
|  | Missing | **143776** |  | 261994 | 108300 | 170847 | 171843 | 96996 | 87751 | 84478 |
| IMD | 1 | **146640 (27.5%)** |  | 295859 (27.4%) | 187670 (28.5%) | 243418 (27.8%) | 236532 (27.7%) | 175100 (28.6%) | 170860 (29%) | 164596 (29%) |
|  | 2 | **110878 (20.8%)** |  | 226104 (20.9%) | 137443 (20.9%) | 181801 (20.8%) | 177327 (20.8%) | 127907 (20.9%) | 123627 (21%) | 119274 (21%) |
|  | 3 | **95657 (17.9%)** |  | 194499 (18%) | 117870 (17.9%) | 157619 (18%) | 154176 (18.1%) | 109653 (17.9%) | 104996 (17.8%) | 101312 (17.9%) |
|  | 4 | **89973 (16.9%)** |  | 182039 (16.9%) | 108337 (16.5%) | 146075 (16.7%) | 142993 (16.7%) | 100405 (16.4%) | 96074 (16.3%) | 92442 (16.3%) |
|  | 5 | **89987 (16.9%)** |  | 181847 (16.8%) | 106431 (16.2%) | 145354 (16.6%) | 142663 (16.7%) | 98466 (16.1%) | 93453 (15.9%) | 89863 (15.8%) |
|  | Missing | **271** |  | 694 | 446 | 551 | 529 | 414 | 395 | 381 |
| Region | NE | **25637 (4.8%)** |  | 49876 (4.6%) | 32124 (4.9%) | 40121 (4.6%) | 39132 (4.6%) | 30416 (5%) | 29687 (5%) | 28878 (5.1%) |
|  | NW | **72311 (13.6%)** |  | 146006 (13.5%) | 90521 (13.8%) | 116210 (13.3%) | 112972 (13.2%) | 84797 (13.9%) | 82510 (14%) | 79805 (14.1%) |
|  | Yorks & Hum | **53150 (10%)** |  | 108191 (10%) | 70880 (10.8%) | 93785 (10.7%) | 93052 (10.9%) | 67234 (11%) | 64688 (11%) | 62737 (11%) |
|  | E Mid | **43500 (8.2%)** |  | 88003 (8.1%) | 52710 (8%) | 70714 (8.1%) | 70505 (8.3%) | 49900 (8.2%) | 47425 (8%) | 45938 (8.1%) |
|  | W Mid | **59256 (11.1%)** |  | 120002 (11.1%) | 72779 (11.1%) | 96094 (11%) | 94674 (11.1%) | 68581 (11.2%) | 65954 (11.2%) | 63768 (11.2%) |
|  | E Eng | **54672 (10.2%)** |  | 111899 (10.4%) | 65942 (10%) | 90597 (10.4%) | 90019 (10.5%) | 61555 (10.1%) | 58226 (9.9%) | 56152 (9.9%) |
|  | Lon | **97543 (18.3%)** |  | 198657 (18.4%) | 110902 (16.8%) | 154481 (17.7%) | 146877 (17.2%) | 98876 (16.2%) | 96279 (16.3%) | 91066 (16%) |
|  | SE | **82219 (15.4%)** |  | 166598 (15.4%) | 98206 (14.9%) | 130076 (14.9%) | 126940 (14.9%) | 91012 (14.9%) | 86833 (14.7%) | 83772 (14.8%) |
|  | SW | **45118 (8.5%)** |  | 91810 (8.5%) | 64133 (9.7%) | 82740 (9.5%) | 80049 (9.4%) | 59574 (9.7%) | 57803 (9.8%) | 55752 (9.8%) |
| Low birth | Yes | **24752 (6.2%)** |  | 51692 (6.2%) | 38160 (7.5%) | 43760 (6.5%) | 40706 (6.1%) | 34536 (7.3%) | 33984 (7.5%) | 32259 (7.3%) |
| weight | Missing | **132099** |  | 250622 | 152319 | 198874 | 188116 | 136644 | 133877 | 127048 |
| Birth | Normal | **376555 (93.8%)** |  | 778728 (93.8%) | 467718 (92.5%) | 632184 (93.5%) | 625398 (93.9%) | 440765 (92.7%) | 421544 (92.5%) | 408561 (92.7%) |
| weight | Low | **21165 (5.3%)** |  | 44135 (5.3%) | 32192 (6.4%) | 37635 (5.6%) | 35290 (5.3%) | 29305 (6.2%) | 28763 (6.3%) | 27322 (6.2%) |
| category | Very Low | **2236 (0.6%)** |  | 4636 (0.6%) | 3982 (0.8%) | 4063 (0.6%) | 3640 (0.5%) | 3546 (0.7%) | 3559 (0.8%) | 3348 (0.8%) |
|  | Extr low | **1351 (0.3%)** |  | 2921 (0.4%) | 1986 (0.4%) | 2062 (0.3%) | 1776 (0.3%) | 1685 (0.4%) | 1662 (0.4%) | 1589 (0.4%) |
|  | Missing | **132099** |  | 250622 | 152319 | 198874 | 188116 | 136644 | 133877 | 127048 |
| Premature | Yes | **23197 (6.2%)** |  | 47956 (6.4%) | 35837 (7.8%) | 40653 (6.6%) | 37682 (6.3%) | 32423 (7.5%) | 32011 (7.8%) | 30262 (7.6%) |
|  | Missing | **161731** |  | 327154 | 200437 | 263358 | 252455 | 182084 | 176684 | 168949 |
| Gest. age | Term | **348478 (93.8%)** |  | 705932 (93.6%) | 421923 (92.2%) | 570807 (93.4%) | 564083 (93.7%) | 397438 (92.5%) | 380710 (92.2%) | 368657 (92.4%) |
|  | Prem | **19576 (5.3%)** |  | 40477 (5.4%) | 29873 (6.5%) | 34574 (5.7%) | 32348 (5.4%) | 27214 (6.3%) | 26780 (6.5%) | 25357 (6.4%) |
|  | Very prem | **2439 (0.7%)** |  | 5035 (0.7%) | 4326 (0.9%) | 4396 (0.7%) | 3909 (0.6%) | 3834 (0.9%) | 3860 (0.9%) | 3607 (0.9%) |
|  | Extr prem | **1182 (0.3%)** |  | 2444 (0.3%) | 1638 (0.4%) | 1683 (0.3%) | 1425 (0.2%) | 1375 (0.3%) | 1371 (0.3%) | 1298 (0.3%) |
|  | Missing | **161731** |  | 327154 | 200437 | 263358 | 252455 | 182084 | 176684 | 168949 |
| Mother | Yes | **37104 (7.3%)** |  | 74665 (7.2%) | 47546 (7.6%) | 61161 (7.3%) | 59532 (7.2%) | 44894 (7.7%) | 43951 (7.8%) | 42605 (7.8%) |
| <20 yrs | Missing | **27174** |  | 48006 | 32770 | 38709 | 31600 | 25367 | 26746 | 23622 |
| Multiple birth |  | **6126 (1.1%)** |  | 10977 (1%) | 8654 (1.3%) | 10080 (1.2%) | 5491 (0.6%) | 4502 (0.7%) | 7561 (1.3%) | 4139 (0.7%) |

Percentages are excluding children with missing values. See Table 2 of the main manuscript for detailed definitions of each sensitivity analysis. Extr: Extremely; Gest. age: Gestational age; IMD: Index of Multiple Deprivation fifths (1 = most deprived, 5 = least deprived); Prem: premature; Regions codes: NE North East; NW North West; Yorks & Hum Yorkshire & The Humber; E Mid East Midlands; W Mid West Midlands; E Eng East of England; Lon London; SE South East; SW South West.

# Table S5. Cumulative incidence of being admitted to hospital and having any record indicating a chronic health condition before age 16 in the main analysis (underlying data for Figures 1 and 2)

|  | Age | | | | | | | | | | | | | | | | |
| --- | --- | --- | --- | --- | --- | --- | --- | --- | --- | --- | --- | --- | --- | --- | --- | --- | --- |
|  | 0 | 1 | 2 | 3 | 4 | 5 | 6 | 7 | 8 | 9 | 10 | 11 | 12 | 13 | 14 | 15 | 16 |
|  |  |  |  |  |  |  |  |  |  |  |  |  |  |  |  |  |  |
| Any chronic health condition |  |  |  |  |  |  |  |  |  |  |  |  |  |  |  |  |  |
|  |  |  |  |  |  |  |  |  |  |  |  |  |  |  |  |  |  |
| 2002/3 | 3.5% | 7.0% | 9.4% | 11.0% | 12.6% | 14.1% | 15.4% | 16.5% | 17.3% | 18.1% | 18.8% | 19.5% | 20.2% | 21.0% | 22.0% | 23.4% | 24.7% |
| 2003/4 | 3.3% | 6.7% | 9.0% | 10.6% | 12.2% | 13.7% | 15.1% | 16.2% | 17.0% | 17.8% | 18.5% | 19.2% | 19.9% | 20.8% | 21.8% | 23.1% | 24.5% |
| 2004/5 | 3.3% | 6.7% | 9.0% | 10.6% | 12.3% | 13.8% | 15.2% | 16.3% | 17.1% | 17.9% | 18.6% | 19.3% | 20.1% | 20.9% | 22.1% | 23.3% |  |
| 2005/6 | 3.4% | 6.9% | 9.1% | 10.7% | 12.3% | 13.9% | 15.2% | 16.3% | 17.2% | 17.9% | 18.6% | 19.3% | 20.1% | 21.0% | 22.0% |  |  |
| 2006/7 | 3.4% | 6.8% | 9.0% | 10.6% | 12.2% | 13.8% | 15.2% | 16.3% | 17.1% | 17.8% | 18.6% | 19.3% | 20.1% | 20.9% |  |  |  |
| 2008/9 | 3.6% | 7.0% | 9.2% | 10.9% | 12.6% | 14.2% | 15.6% | 16.6% | 17.5% | 18.2% | 19.0% | 19.7% | 20.4% |  |  |  |  |
| 2009/10 | 3.7% | 7.1% | 9.4% | 11.0% | 12.6% | 14.2% | 15.6% | 16.6% | 17.5% | 18.3% | 19.1% | 19.8% |  |  |  |  |  |
| 2010/11 | 3.8% | 7.3% | 9.5% | 11.0% | 12.6% | 14.2% | 15.5% | 16.6% | 17.5% | 18.3% | 19.0% |  |  |  |  |  |  |
| 2011/12 | 3.8% | 7.2% | 9.0% | 10.5% | 12.1% | 13.6% | 15.0% | 16.1% | 17.0% | 17.8% |  |  |  |  |  |  |  |
|  |  |  |  |  |  |  |  |  |  |  |  |  |  |  |  |  |  |
| Cancer/ blood |  |  |  |  |  |  |  |  |  |  |  |  |  |  |  |  |  |
|  |  |  |  |  |  |  |  |  |  |  |  |  |  |  |  |  |  |
| 2002/3 & 2003/4 | 0.1% | 0.3% | 0.4% | 0.5% | 0.7% | 0.8% | 0.9% | 1.0% | 1.1% | 1.2% | 1.3% | 1.4% | 1.5% | 1.6% | 1.6% | 1.7% | 1.8% |
| 2004/5 & 2005/6 | 0.1% | 0.3% | 0.5% | 0.6% | 0.7% | 0.8% | 1.0% | 1.1% | 1.2% | 1.3% | 1.4% | 1.5% | 1.5% | 1.6% | 1.7% | 1.7% |  |
| 2006/7 & 2007/8 | 0.1% | 0.3% | 0.5% | 0.6% | 0.8% | 0.9% | 1.1% | 1.2% | 1.3% | 1.4% | 1.5% | 1.5% | 1.6% | 1.6% |  |  |  |
| 2008/9 & 2009/10 | 0.1% | 0.4% | 0.6% | 0.8% | 1.0% | 1.1% | 1.3% | 1.3% | 1.4% | 1.5% | 1.6% | 1.6% |  |  |  |  |  |
| 2010/11 & 2011/12 | 0.1% | 0.6% | 0.8% | 1.0% | 1.1% | 1.2% | 1.4% | 1.4% | 1.5% | 1.6% |  |  |  |  |  |  |  |
|  |  |  |  |  |  |  |  |  |  |  |  |  |  |  |  |  |  |
| Cardiovascular |  |  |  |  |  |  |  |  |  |  |  |  |  |  |  |  |  |
|  |  |  |  |  |  |  |  |  |  |  |  |  |  |  |  |  |  |
| 2002/3 & 2003/4 | 0.8% | 1.0% | 1.1% | 1.2% | 1.2% | 1.3% | 1.3% | 1.3% | 1.4% | 1.4% | 1.4% | 1.4% | 1.5% | 1.5% | 1.6% | 1.6% | 1.7% |
| 2004/5 & 2005/6 | 0.8% | 1.0% | 1.1% | 1.2% | 1.2% | 1.3% | 1.3% | 1.3% | 1.4% | 1.4% | 1.4% | 1.5% | 1.5% | 1.5% | 1.6% | 1.6% |  |
| 2006/7 & 2007/8 | 0.9% | 1.2% | 1.2% | 1.3% | 1.3% | 1.4% | 1.4% | 1.5% | 1.5% | 1.5% | 1.6% | 1.6% | 1.6% | 1.7% |  |  |  |
| 2008/9 & 2009/10 | 1.0% | 1.3% | 1.4% | 1.5% | 1.5% | 1.6% | 1.6% | 1.6% | 1.7% | 1.7% | 1.7% | 1.8% |  |  |  |  |  |
| 2010/11 & 2011/12 | 1.1% | 1.4% | 1.5% | 1.6% | 1.6% | 1.7% | 1.7% | 1.8% | 1.8% | 1.8% |  |  |  |  |  |  |  |
|  |  |  |  |  |  |  |  |  |  |  |  |  |  |  |  |  |  |
|  |  |  |  |  |  |  |  |  |  |  |  |  |  |  |  |  |  |
| Mental health/ behavioural |  |  |  |  |  |  |  |  |  |  |  |  |  |  |  |  |  |
|  |  |  |  |  |  |  |  |  |  |  |  |  |  |  |  |  |  |
| 2002/3 & 2003/4 | ~0.0% | ~0.0% | 0.1% | 0.2% | 0.3% | 0.4% | 0.6% | 0.7% | 0.9% | 1.1% | 1.3% | 1.5% | 1.8% | 2.1% | 2.7% | 3.7% | 4.7% |
| 2004/5 & 2005/6 | ~0.0% | ~0.0% | 0.1% | 0.2% | 0.4% | 0.5% | 0.7% | 0.9% | 1.1% | 1.3% | 1.5% | 1.8% | 2.1% | 2.5% | 3.2% | 3.7% |  |
| 2006/7 & 2007/8 | ~0.0% | ~0.0% | 0.1% | 0.3% | 0.4% | 0.7% | 0.9% | 1.1% | 1.3% | 1.6% | 1.8% | 2.1% | 2.4% | 2.7% |  |  |  |
| 2008/9 & 2009/10 | ~0.0% | 0.1% | 0.2% | 0.4% | 0.7% | 0.9% | 1.2% | 1.4% | 1.7% | 2.0% | 2.2% | 2.4% |  |  |  |  |  |
| 2010/11 & 2011/12 | ~0.0% | 0.1% | 0.2% | 0.5% | 0.8% | 1.1% | 1.4% | 1.6% | 1.9% | 2.1% |  |  |  |  |  |  |  |
|  |  |  |  |  |  |  |  |  |  |  |  |  |  |  |  |  |  |
| Metabolic, &c |  |  |  |  |  |  |  |  |  |  |  |  |  |  |  |  |  |
|  |  |  |  |  |  |  |  |  |  |  |  |  |  |  |  |  |  |
| 2002/3 & 2003/4 | 1.2% | 2.9% | 4.0% | 4.5% | 4.8% | 5.1% | 5.3% | 5.6% | 5.8% | 6.0% | 6.2% | 6.4% | 6.6% | 6.8% | 7.1% | 7.4% | 7.8% |
| 2004/5 & 2005/6 | 1.2% | 3.0% | 4.0% | 4.5% | 4.9% | 5.2% | 5.5% | 5.7% | 5.8% | 6.0% | 6.2% | 6.4% | 6.6% | 6.9% | 7.2% | 7.4% |  |
| 2006/7 & 2007/8 | 1.2% | 3.0% | 4.1% | 4.6% | 5.0% | 5.2% | 5.4% | 5.6% | 5.7% | 5.9% | 6.1% | 6.4% | 6.6% | 6.8% |  |  |  |
| 2008/9 & 2009/10 | 1.3% | 3.2% | 4.2% | 4.6% | 4.8% | 5.0% | 5.2% | 5.4% | 5.6% | 5.8% | 6.0% | 6.2% |  |  |  |  |  |
| 2010/11 & 2011/12 | 1.3% | 2.6% | 3.1% | 3.3% | 3.5% | 3.7% | 3.9% | 4.1% | 4.4% | 4.5% |  |  |  |  |  |  |  |
|  |  |  |  |  |  |  |  |  |  |  |  |  |  |  |  |  |  |
| Musculoskeletal/ skin |  |  |  |  |  |  |  |  |  |  |  |  |  |  |  |  |  |
|  |  |  |  |  |  |  |  |  |  |  |  |  |  |  |  |  |  |
| 2002/3 & 2003/4 | 0.3% | 0.6% | 0.7% | 0.8% | 1.0% | 1.1% | 1.2% | 1.3% | 1.4% | 1.5% | 1.6% | 1.7% | 1.9% | 2.1% | 2.3% | 2.6% | 2.9% |
| 2004/5 & 2005/6 | 0.3% | 0.6% | 0.7% | 0.9% | 1.0% | 1.1% | 1.2% | 1.3% | 1.4% | 1.6% | 1.7% | 1.8% | 2.0% | 2.2% | 2.5% | 2.6% |  |
| 2006/7 & 2007/8 | 0.3% | 0.6% | 0.7% | 0.9% | 1.0% | 1.1% | 1.2% | 1.4% | 1.5% | 1.6% | 1.7% | 1.9% | 2.1% | 2.2% |  |  |  |
| 2008/9 & 2009/10 | 0.3% | 0.6% | 0.8% | 0.9% | 1.1% | 1.2% | 1.3% | 1.4% | 1.6% | 1.7% | 1.8% | 1.9% |  |  |  |  |  |
| 2010/11 & 2011/12 | 0.4% | 0.6% | 0.9% | 1.0% | 1.2% | 1.3% | 1.4% | 1.6% | 1.7% | 1.8% |  |  |  |  |  |  |  |
|  |  |  |  |  |  |  |  |  |  |  |  |  |  |  |  |  |  |
| Neurological |  |  |  |  |  |  |  |  |  |  |  |  |  |  |  |  |  |
|  |  |  |  |  |  |  |  |  |  |  |  |  |  |  |  |  |  |
| 2002/3 & 2003/4 | 1.0% | 1.7% | 2.4% | 2.9% | 3.6% | 4.4% | 5.1% | 5.6% | 5.9% | 6.1% | 6.3% | 6.4% | 6.6% | 6.8% | 7.1% | 7.3% | 7.6% |
| 2004/5 & 2005/6 | 0.9% | 1.7% | 2.3% | 2.8% | 3.5% | 4.2% | 4.8% | 5.3% | 5.6% | 5.8% | 6.0% | 6.2% | 6.4% | 6.6% | 6.8% | 7.0% |  |
| 2006/7 & 2007/8 | 1.0% | 1.8% | 2.3% | 2.9% | 3.5% | 4.2% | 4.8% | 5.3% | 5.6% | 5.8% | 6.0% | 6.2% | 6.3% | 6.5% |  |  |  |
| 2008/9 & 2009/10 | 1.1% | 1.8% | 2.4% | 2.9% | 3.5% | 4.2% | 4.8% | 5.2% | 5.5% | 5.7% | 5.9% | 6.0% |  |  |  |  |  |
| 2010/11 & 2011/12 | 1.1% | 1.9% | 2.4% | 3.0% | 3.5% | 4.2% | 4.7% | 5.1% | 5.4% | 5.5% |  |  |  |  |  |  |  |
|  |  |  |  |  |  |  |  |  |  |  |  |  |  |  |  |  |  |
| Non-specific |  |  |  |  |  |  |  |  |  |  |  |  |  |  |  |  |  |
|  |  |  |  |  |  |  |  |  |  |  |  |  |  |  |  |  |  |
| 2002/3 & 2003/4 | 0.2% | 1.1% | 1.4% | 1.5% | 1.6% | 1.7% | 1.7% | 1.8% | 1.8% | 1.9% | 1.9% | 1.9% | 1.9% | 2.0% | 2.0% | 2.0% | 2.1% |
| 2004/5 & 2005/6 | 0.2% | 1.1% | 1.4% | 1.5% | 1.6% | 1.7% | 1.8% | 1.8% | 1.8% | 1.9% | 1.9% | 1.9% | 1.9% | 2.0% | 2.0% |  |  |
| 2006/7 & 2007/8 | 0.2% | 1.1% | 1.4% | 1.5% | 1.7% | 1.7% | 1.8% | 1.8% | 1.9% | 1.9% | 1.9% | 2.0% | 2.0% |  |  |  |  |
| 2008/9 & 2009/10 | 0.2% | 1.2% | 1.5% | 1.6% | 1.8% | 1.8% | 1.9% | 1.9% | 2.0% | 2.0% | 2.0% |  |  |  |  |  |  |
| 2010/11 & 2011/12 | 0.3% | 1.3% | 1.5% | 1.7% | 1.8% | 1.8% | 1.9% | 2.0% | 2.0% | 2.0% |  |  |  |  |  |  |  |
|  |  |  |  |  |  |  |  |  |  |  |  |  |  |  |  |  |  |
| Respiratory |  |  |  |  |  |  |  |  |  |  |  |  |  |  |  |  |  |
|  |  |  |  |  |  |  |  |  |  |  |  |  |  |  |  |  |  |
| 2002/3 & 2003/4 | 0.5% | 1.0% | 1.7% | 2.3% | 3.0% | 3.6% | 4.2% | 4.6% | 5.0% | 5.3% | 5.6% | 6.0% | 6.3% | 6.6% | 6.9% | 7.3% | 7.6% |
| 2004/5 & 2005/6 | 0.4% | 0.9% | 1.6% | 2.2% | 3.0% | 3.7% | 4.3% | 4.8% | 5.2% | 5.5% | 5.8% | 6.2% | 6.5% | 6.8% | 7.1% | 7.3% |  |
| 2006/7 & 2007/8 | 0.5% | 1.0% | 1.6% | 2.2% | 3.0% | 3.8% | 4.5% | 5.0% | 5.4% | 5.7% | 6.1% | 6.4% | 6.7% | 6.8% |  |  |  |
| 2008/9 & 2009/10 | 0.5% | 1.0% | 1.6% | 2.3% | 3.2% | 4.0% | 4.8% | 5.3% | 5.7% | 6.1% | 6.4% | 6.6% |  |  |  |  |  |
| 2010/11 & 2011/12 | 0.5% | 1.0% | 1.6% | 2.3% | 3.2% | 4.1% | 4.8% | 5.4% | 5.8% | 6.0% |  |  |  |  |  |  |  |
|  |  |  |  |  |  |  |  |  |  |  |  |  |  |  |  |  |  |

The Metabolic, &c group includes metabolic, endocrine, digestive, renal and genitourinary conditions. See Supplementary Table S1 for a full list of codes by each group.

# Table S6. The twenty most frequent ICD-10 diagnostic codes detected in each sub-type in the 2002/3 and 2003/4 cohorts grouped by age of first detection (birth to age 4, age 5 to 10 and age 11 to 15); codes are only counted once per child, rather than once per admission

| ICD-10 code | Condition type | n |
| --- | --- | --- |
|  |  |  |
| **Birth to age 4** | | |
|  |  |  |
| D69: Purpura and other haemorrhagic conditions | Cancer/ Blood | 2115 |
| D70: Agranulocytosis | Cancer/ Blood | 1083 |
| D64: Other anaemias | Cancer/ Blood | 847 |
| Z511: Chemotherapy session for neoplasm | Cancer/ Blood | 735 |
| D50: Iron deficiency anaemia | Cancer/ Blood | 575 |
| Z512: Other chemotherapy | Cancer/ Blood | 456 |
| Y433: Other antineoplastic drugs | Cancer/ Blood | 328 |
| Z85: Personal history of malignant neoplasm | Cancer/ Blood | 326 |
| C91: Lymphoid leukaemia | Cancer/ Blood | 318 |
| D571: Sickle-cell anaemia without crisis | Cancer/ Blood | 259 |
| D689: Coagulation defect unspecified | Cancer/ Blood | 217 |
| D58: Other hereditary haemolytic anaemias | Cancer/ Blood | 192 |
| D570: Sickle-cell anaemia with crisis | Cancer/ Blood | 162 |
| Z08: Follow-up examination after treatment for malignant neoplasms | Cancer/ Blood | 155 |
| C71: Malignant neoplasm of brain | Cancer/ Blood | 154 |
| D80: Immunodeficiency with predominantly antibody defects | Cancer/ Blood | 148 |
| D82: Immunodeficiency associated with other major defects | Cancer/ Blood | 124 |
| D48: Neoplasm of uncertain or unknown behaviour of other and unspecified sites | Cancer/ Blood | 121 |
| Z862: Personal history of diseases of the blood and blood-forming organs and certain disorders involving the immune mechanism | Cancer/ Blood | 116 |
| D72: Other disorders of white blood cells | Cancer/ Blood | 111 |
| Q25: Congenital malformations of great arteries | Cardiovascular | 5538 |
| Q21: Congenital malformations of cardiac septa | Cardiovascular | 5285 |
| Q24: Other congenital malformations of heart | Cardiovascular | 1591 |
| Q22: Congenital malformations of pulmonary and tricuspid valves | Cardiovascular | 986 |
| T827: Infection and inflammatory reaction due to other cardiac and vascular devices implants and grafts | Cardiovascular | 893 |
| Q20: Congenital malformations of cardiac chambers and connections | Cardiovascular | 797 |
| Q23: Congenital malformations of aortic and mitral valves | Cardiovascular | 797 |
| Z95: Presence of cardiac and vascular implants and grafts | Cardiovascular | 579 |
| I27: Other pulmonary heart diseases | Cardiovascular | 464 |
| I50: Heart failure | Cardiovascular | 378 |
| Q27: Other congenital malformations of peripheral vascular system | Cardiovascular | 372 |
| I47: Paroxysmal tachycardia | Cardiovascular | 281 |
| Q26: Congenital malformations of great veins | Cardiovascular | 276 |
| T828: Other specified complications of cardiac and vascular prosthetic devices implants and grafts | Cardiovascular | 248 |
| T825: Mechanical complication of other cardiac and vascular devices and implants | Cardiovascular | 237 |
| I31: Other diseases of pericardium | Cardiovascular | 234 |
| I46: Cardiac arrest | Cardiovascular | 230 |
| I10: Essential (primary) hypertension | Cardiovascular | 227 |
| I51: Complications and ill-defined descriptions of heart disease | Cardiovascular | 195 |
| I49: Other cardiac arrhythmias | Cardiovascular | 123 |
| M00: Pyogenic arthritis | Chronic infections | 388 |
| P351: Congenital cytomegalovirus infection | Chronic infections | 103 |
| B94: Sequelae of other and unspecified infectious and parasitic diseases | Chronic infections | 92 |
| A16: Respiratory tuberculosis not confirmed bacteriologically or histologically | Chronic infections | 63 |
| P359: Congenital viral disease unspecified | Chronic infections | 53 |
| P358: Other congenital viral diseases | Chronic infections | 50 |
| A18: Tuberculosis of other organs | Chronic infections | 44 |
| P352: Congenital herpesviral [herpes simplex] infection | Chronic infections | 37 |
| B377: Candidal sepsis | Chronic infections | 30 |
| A15: Respiratory tuberculosis bacteriologically and histologically confirmed | Chronic infections | 19 |
| A50: Congenital syphilis | Chronic infections | 18 |
| P371: Congenital toxoplasmosis | Chronic infections | 18 |
| B18: Chronic viral hepatitis | Chronic infections | 17 |
| B59: Pneumocystosis | Chronic infections | 16 |
| B24: Unspecified human immunodeficiency virus [HIV] disease | Chronic infections | 15 |
| A17: Tuberculosis of nervous system | Chronic infections | 12 |
| Z21: Asymptomatic human immunodeficiency virus [HIV] infection status | Chronic infections | 12 |
| B20: Human immunodeficiency virus [HIV] disease resulting in infectious and parasitic diseases | Chronic infections | 11 |
| R62: Lack of expected normal physiological development | Codes indicating non-specific chronic condition | 9997 |
| R633: Feeding difficulties and mismanagement | Codes indicating non-specific chronic condition | 6568 |
| Z931: Gastrostomy status | Codes indicating non-specific chronic condition | 1077 |
| Z431: Attention to gastrostomy | Codes indicating non-specific chronic condition | 554 |
| Z755: Holiday relief care | Codes indicating non-specific chronic condition | 148 |
| Z515: Palliative care | Codes indicating non-specific chronic condition | 40 |
| Z993: Dependence on wheelchair | Codes indicating non-specific chronic condition | 27 |
| F809: Developmental disorder of speech and language unspecified | Mental health/ behavioural | 1122 |
| F81: Specific developmental disorders of scholastic skills | Mental health/ behavioural | 870 |
| F84: Pervasive developmental disorders | Mental health/ behavioural | 682 |
| F79: Unspecified mental retardation | Mental health/ behavioural | 219 |
| F82: Specific developmental disorder of motor function | Mental health/ behavioural | 179 |
| F91: Conduct disorders | Mental health/ behavioural | 178 |
| F83: Mixed specific developmental disorders | Mental health/ behavioural | 170 |
| F98: Other behavioural and emotional disorders with onset usually occurring in childhood and adolescence | Mental health/ behavioural | 170 |
| F89: Unspecified disorder of psychological development | Mental health/ behavioural | 116 |
| F90: Hyperkinetic disorders | Mental health/ behavioural | 108 |
| X60: Intentional self-poisoning by and exposure to nonopioid analgesics antipyretics and antirheumatics | Mental health/ behavioural | 69 |
| X64: Intentional self-poisoning by and exposure to other and unspecified drugs medicaments and biological substances | Mental health/ behavioural | 51 |
| Y47: Sedatives hypnotics and antianxiety drugs | Mental health/ behavioural | 50 |
| F808: Other developmental disorders of speech and language | Mental health/ behavioural | 38 |
| F40: Phobic anxiety disorders | Mental health/ behavioural | 31 |
| F801: Expressive language disorder | Mental health/ behavioural | 27 |
| F11: Mental and behavioural disorders due to use of opioids | Mental health/ behavioural | 24 |
| F70: Mild mental retardation | Mental health/ behavioural | 24 |
| F50: Eating disorders | Mental health/ behavioural | 23 |
| F41: Other anxiety disorders | Mental health/ behavioural | 21 |
| K52: Other noninfective gastroenteritis and colitis | Metabolic/ endocrine/ digestive/ renal/ genitourinary | 25819 |
| Q549: Hypospadias unspecified | Metabolic/ endocrine/ digestive/ renal/ genitourinary | 2726 |
| Q620: Congenital hydronephrosis | Metabolic/ endocrine/ digestive/ renal/ genitourinary | 1782 |
| K297: Gastritis unspecified | Metabolic/ endocrine/ digestive/ renal/ genitourinary | 1555 |
| N13: Obstructive and reflux uropathy | Metabolic/ endocrine/ digestive/ renal/ genitourinary | 1504 |
| K90: Intestinal malabsorption | Metabolic/ endocrine/ digestive/ renal/ genitourinary | 1447 |
| Q623: Other obstructive defects of renal pelvis and ureter | Metabolic/ endocrine/ digestive/ renal/ genitourinary | 1330 |
| Q540: Hypospadias balanic | Metabolic/ endocrine/ digestive/ renal/ genitourinary | 1228 |
| N28: Other disorders of kidney and ureter not elsewhere classified | Metabolic/ endocrine/ digestive/ renal/ genitourinary | 1061 |
| Q541: Hypospadias penile | Metabolic/ endocrine/ digestive/ renal/ genitourinary | 818 |
| Q61: Cystic kidney disease | Metabolic/ endocrine/ digestive/ renal/ genitourinary | 774 |
| E10: Type 1 diabetes mellitus | Metabolic/ endocrine/ digestive/ renal/ genitourinary | 741 |
| Q64: Other congenital malformations of urinary system | Metabolic/ endocrine/ digestive/ renal/ genitourinary | 734 |
| P960: Congenital renal failure | Metabolic/ endocrine/ digestive/ renal/ genitourinary | 613 |
| E83: Disorders of mineral metabolism | Metabolic/ endocrine/ digestive/ renal/ genitourinary | 579 |
| K210: Gastro-oesophageal reflux disease with oesophagitis | Metabolic/ endocrine/ digestive/ renal/ genitourinary | 551 |
| K22: Other diseases of oesophagus | Metabolic/ endocrine/ digestive/ renal/ genitourinary | 509 |
| Q638: Other specified congenital malformations of kidney | Metabolic/ endocrine/ digestive/ renal/ genitourinary | 466 |
| K296: Other gastritis | Metabolic/ endocrine/ digestive/ renal/ genitourinary | 464 |
| Q42: Congenital absence atresia and stenosis of large intestine | Metabolic/ endocrine/ digestive/ renal/ genitourinary | 461 |
| Q74: Other congenital malformations of limb(s) | Musculoskeletal/ skin | 911 |
| Q650: Congenital dislocation of hip unilateral | Musculoskeletal/ skin | 908 |
| Q658: Other congenital deformities of hip | Musculoskeletal/ skin | 772 |
| L92: Granulomatous disorders of skin and subcutaneous tissue | Musculoskeletal/ skin | 571 |
| Q759: Congenital malformation of skull and face bones unspecified | Musculoskeletal/ skin | 462 |
| M54: Dorsalgia | Musculoskeletal/ skin | 399 |
| Q870: Congenital malformation syndromes predominantly affecting facial appearance | Musculoskeletal/ skin | 388 |
| Q652: Congenital dislocation of hip unspecified | Musculoskeletal/ skin | 367 |
| M08: Juvenile arthritis | Musculoskeletal/ skin | 352 |
| M30: Polyarteritis nodosa and related conditions | Musculoskeletal/ skin | 327 |
| Q651: Congenital dislocation of hip bilateral | Musculoskeletal/ skin | 325 |
| M43: Other deforming dorsopathies | Musculoskeletal/ skin | 290 |
| Q764: Other congenital malformations of spine not associated with scoliosis | Musculoskeletal/ skin | 283 |
| Q71: Reduction defects of upper limb | Musculoskeletal/ skin | 259 |
| L90: Atrophic disorders of skin | Musculoskeletal/ skin | 237 |
| M41: Scoliosis | Musculoskeletal/ skin | 236 |
| M62: Other disorders of muscle | Musculoskeletal/ skin | 228 |
| Q753: Macrocephaly | Musculoskeletal/ skin | 213 |
| M13: Other arthritis | Musculoskeletal/ skin | 210 |
| Q78: Other osteochondrodysplasias | Musculoskeletal/ skin | 207 |
| H653: Chronic mucoid otitis media | Neurological | 13833 |
| R568: Other and unspecified convulsions | Neurological | 7320 |
| H91: Other hearing loss | Neurological | 2468 |
| P210: Severe birth asphyxia | Neurological | 2335 |
| G409: Epilepsy unspecified | Neurological | 2228 |
| P90: Convulsions of newborn | Neurological | 1937 |
| P52: Intracranial nontraumatic haemorrhage of fetus and newborn | Neurological | 1905 |
| G80: Cerebral palsy | Neurological | 1723 |
| Q90: Down syndrome | Neurological | 1134 |
| H35: Other retinal disorders | Neurological | 1066 |
| G93: Other disorders of brain | Neurological | 1063 |
| Q04: Other congenital malformations of brain | Neurological | 1018 |
| G403: Generalized idiopathic epilepsy and epileptic syndromes | Neurological | 943 |
| Q02: Microcephaly | Neurological | 824 |
| G00: Bacterial meningitis not elsewhere classified | Neurological | 775 |
| H654: Other chronic nonsuppurative otitis media | Neurological | 705 |
| G91: Hydrocephalus | Neurological | 693 |
| G41: Status epilepticus | Neurological | 586 |
| Z982: Presence of cerebrospinal fluid drainage device | Neurological | 526 |
| Q750: Craniosynostosis | Neurological | 512 |
| J45: Asthma | Respiratory | 20099 |
| G473: Sleep apnoea | Respiratory | 3760 |
| J98: Other respiratory disorders | Respiratory | 3330 |
| P27: Chronic respiratory disease originating in the perinatal period | Respiratory | 2225 |
| J46: Status asthmaticus | Respiratory | 1495 |
| Q31: Congenital malformations of larynx | Respiratory | 1213 |
| Q35: Cleft palate | Respiratory | 1105 |
| Q33: Congenital malformations of lung | Respiratory | 741 |
| Q37: Cleft palate with cleft lip | Respiratory | 669 |
| Q36: Cleft lip | Respiratory | 515 |
| J86: Pyothorax | Respiratory | 422 |
| J69: Pneumonitis due to solids and liquids | Respiratory | 373 |
| Q32: Congenital malformations of trachea and bronchus | Respiratory | 352 |
| E84: Cystic fibrosis | Respiratory | 338 |
| Q790: Congenital diaphragmatic hernia | Respiratory | 279 |
| Z930: Tracheostomy status | Respiratory | 267 |
| Q30: Congenital malformations of nose | Respiratory | 251 |
| J44: Other chronic obstructive pulmonary disease | Respiratory | 173 |
| Z430: Attention to tracheostomy | Respiratory | 157 |
| J81: Pulmonary oedema | Respiratory | 118 |
|  |  |  |
| **Age 5 to 10** | | |
|  |  |  |
| Z512: Other chemotherapy | Cancer/ Blood | 2701 |
| D69: Purpura and other haemorrhagic conditions | Cancer/ Blood | 1995 |
| D70: Agranulocytosis | Cancer/ Blood | 531 |
| Z511: Chemotherapy session for neoplasm | Cancer/ Blood | 448 |
| Z85: Personal history of malignant neoplasm | Cancer/ Blood | 447 |
| Y433: Other antineoplastic drugs | Cancer/ Blood | 248 |
| D64: Other anaemias | Cancer/ Blood | 244 |
| Z08: Follow-up examination after treatment for malignant neoplasms | Cancer/ Blood | 203 |
| Z860: Personal history of other neoplasms | Cancer/ Blood | 152 |
| C71: Malignant neoplasm of brain | Cancer/ Blood | 150 |
| C91: Lymphoid leukaemia | Cancer/ Blood | 149 |
| Z862: Personal history of diseases of the blood and blood-forming organs and certain disorders involving the immune mechanism | Cancer/ Blood | 143 |
| D50: Iron deficiency anaemia | Cancer/ Blood | 117 |
| D680: Von Willebrand disease | Cancer/ Blood | 110 |
| Z923: Personal history of irradiation | Cancer/ Blood | 108 |
| D571: Sickle-cell anaemia without crisis | Cancer/ Blood | 106 |
| D43: Neoplasm of uncertain or unknown behaviour of brain and central nervous system | Cancer/ Blood | 98 |
| D689: Coagulation defect unspecified | Cancer/ Blood | 94 |
| D570: Sickle-cell anaemia with crisis | Cancer/ Blood | 91 |
| D72: Other disorders of white blood cells | Cancer/ Blood | 87 |
| Z95: Presence of cardiac and vascular implants and grafts | Cardiovascular | 424 |
| Q21: Congenital malformations of cardiac septa | Cardiovascular | 422 |
| T827: Infection and inflammatory reaction due to other cardiac and vascular devices implants and grafts | Cardiovascular | 271 |
| Q25: Congenital malformations of great arteries | Cardiovascular | 242 |
| I10: Essential (primary) hypertension | Cardiovascular | 228 |
| T825: Mechanical complication of other cardiac and vascular devices and implants | Cardiovascular | 192 |
| Q23: Congenital malformations of aortic and mitral valves | Cardiovascular | 169 |
| Q24: Other congenital malformations of heart | Cardiovascular | 165 |
| Q27: Other congenital malformations of peripheral vascular system | Cardiovascular | 153 |
| T828: Other specified complications of cardiac and vascular prosthetic devices implants and grafts | Cardiovascular | 141 |
| Q22: Congenital malformations of pulmonary and tricuspid valves | Cardiovascular | 96 |
| I31: Other diseases of pericardium | Cardiovascular | 80 |
| I51: Complications and ill-defined descriptions of heart disease | Cardiovascular | 66 |
| I46: Cardiac arrest | Cardiovascular | 57 |
| I47: Paroxysmal tachycardia | Cardiovascular | 52 |
| Q26: Congenital malformations of great veins | Cardiovascular | 51 |
| I15: Secondary hypertension | Cardiovascular | 49 |
| I50: Heart failure | Cardiovascular | 47 |
| I34: Nonrheumatic mitral valve disorders | Cardiovascular | 41 |
| I35: Nonrheumatic aortic valve disorders | Cardiovascular | 39 |
| M00: Pyogenic arthritis | Chronic infections | 173 |
| B94: Sequelae of other and unspecified infectious and parasitic diseases | Chronic infections | 95 |
| A16: Respiratory tuberculosis not confirmed bacteriologically or histologically | Chronic infections | 31 |
| B371: Pulmonary candidiasis | Chronic infections | 16 |
| A18: Tuberculosis of other organs | Chronic infections | 13 |
| A15: Respiratory tuberculosis bacteriologically and histologically confirmed | Chronic infections | 11 |
| R62: Lack of expected normal physiological development | Codes indicating non-specific chronic condition | 1718 |
| Z993: Dependence on wheelchair | Codes indicating non-specific chronic condition | 521 |
| Z931: Gastrostomy status | Codes indicating non-specific chronic condition | 477 |
| R633: Feeding difficulties and mismanagement | Codes indicating non-specific chronic condition | 386 |
| Z431: Attention to gastrostomy | Codes indicating non-specific chronic condition | 352 |
| Z755: Holiday relief care | Codes indicating non-specific chronic condition | 57 |
| Z515: Palliative care | Codes indicating non-specific chronic condition | 52 |
| F84: Pervasive developmental disorders | Mental health/ behavioural | 3358 |
| F81: Specific developmental disorders of scholastic skills | Mental health/ behavioural | 2685 |
| F90: Hyperkinetic disorders | Mental health/ behavioural | 1970 |
| F40: Phobic anxiety disorders | Mental health/ behavioural | 908 |
| F809: Developmental disorder of speech and language unspecified | Mental health/ behavioural | 736 |
| F89: Unspecified disorder of psychological development | Mental health/ behavioural | 699 |
| F41: Other anxiety disorders | Mental health/ behavioural | 621 |
| F79: Unspecified mental retardation | Mental health/ behavioural | 577 |
| F91: Conduct disorders | Mental health/ behavioural | 499 |
| F83: Mixed specific developmental disorders | Mental health/ behavioural | 255 |
| F98: Other behavioural and emotional disorders with onset usually occurring in childhood and adolescence | Mental health/ behavioural | 234 |
| F82: Specific developmental disorder of motor function | Mental health/ behavioural | 210 |
| F95: Tic disorders | Mental health/ behavioural | 196 |
| Z915: Personal history of self-harm | Mental health/ behavioural | 118 |
| F72: Severe mental retardation | Mental health/ behavioural | 91 |
| F50: Eating disorders | Mental health/ behavioural | 67 |
| F69: Unspecified disorder of adult personality and behaviour | Mental health/ behavioural | 60 |
| F45: Somatoform disorders | Mental health/ behavioural | 56 |
| F70: Mild mental retardation | Mental health/ behavioural | 55 |
| F32: Depressive episode | Mental health/ behavioural | 46 |
| K52: Other noninfective gastroenteritis and colitis | Metabolic/ endocrine/ digestive/ renal/ genitourinary | 3477 |
| E10: Type 1 diabetes mellitus | Metabolic/ endocrine/ digestive/ renal/ genitourinary | 1599 |
| K297: Gastritis unspecified | Metabolic/ endocrine/ digestive/ renal/ genitourinary | 1161 |
| K90: Intestinal malabsorption | Metabolic/ endocrine/ digestive/ renal/ genitourinary | 963 |
| E66: Obesity | Metabolic/ endocrine/ digestive/ renal/ genitourinary | 712 |
| N13: Obstructive and reflux uropathy | Metabolic/ endocrine/ digestive/ renal/ genitourinary | 577 |
| N32: Other disorders of bladder | Metabolic/ endocrine/ digestive/ renal/ genitourinary | 527 |
| N12: Tubulo-interstitial nephritis not specified as acute or chronic | Metabolic/ endocrine/ digestive/ renal/ genitourinary | 449 |
| N35: Urethral stricture | Metabolic/ endocrine/ digestive/ renal/ genitourinary | 405 |
| N28: Other disorders of kidney and ureter not elsewhere classified | Metabolic/ endocrine/ digestive/ renal/ genitourinary | 319 |
| K295: Chronic gastritis unspecified | Metabolic/ endocrine/ digestive/ renal/ genitourinary | 303 |
| K20: Oesophagitis | Metabolic/ endocrine/ digestive/ renal/ genitourinary | 290 |
| N394: Other specified urinary incontinence | Metabolic/ endocrine/ digestive/ renal/ genitourinary | 271 |
| N31: Neuromuscular dysfunction of bladder not elsewhere classified | Metabolic/ endocrine/ digestive/ renal/ genitourinary | 265 |
| K22: Other diseases of oesophagus | Metabolic/ endocrine/ digestive/ renal/ genitourinary | 261 |
| K296: Other gastritis | Metabolic/ endocrine/ digestive/ renal/ genitourinary | 256 |
| E83: Disorders of mineral metabolism | Metabolic/ endocrine/ digestive/ renal/ genitourinary | 228 |
| N05: Unspecified nephritic syndrome | Metabolic/ endocrine/ digestive/ renal/ genitourinary | 225 |
| K50: Crohn disease [regional enteritis] | Metabolic/ endocrine/ digestive/ renal/ genitourinary | 221 |
| K66: Other disorders of peritoneum | Metabolic/ endocrine/ digestive/ renal/ genitourinary | 209 |
| M54: Dorsalgia | Musculoskeletal/ skin | 1036 |
| M41: Scoliosis | Musculoskeletal/ skin | 513 |
| M08: Juvenile arthritis | Musculoskeletal/ skin | 395 |
| L90: Atrophic disorders of skin | Musculoskeletal/ skin | 352 |
| M62: Other disorders of muscle | Musculoskeletal/ skin | 346 |
| M35: Other systemic involvement of connective tissue | Musculoskeletal/ skin | 331 |
| M89: Other disorders of bone | Musculoskeletal/ skin | 312 |
| M91: Juvenile osteochondrosis of hip and pelvis | Musculoskeletal/ skin | 299 |
| L92: Granulomatous disorders of skin and subcutaneous tissue | Musculoskeletal/ skin | 282 |
| M43: Other deforming dorsopathies | Musculoskeletal/ skin | 260 |
| M60: Myositis | Musculoskeletal/ skin | 251 |
| Q74: Other congenital malformations of limb(s) | Musculoskeletal/ skin | 216 |
| Q658: Other congenital deformities of hip | Musculoskeletal/ skin | 214 |
| M85: Other disorders of bone density and structure | Musculoskeletal/ skin | 188 |
| M13: Other arthritis | Musculoskeletal/ skin | 176 |
| Q78: Other osteochondrodysplasias | Musculoskeletal/ skin | 126 |
| L40: Psoriasis | Musculoskeletal/ skin | 120 |
| M30: Polyarteritis nodosa and related conditions | Musculoskeletal/ skin | 118 |
| M94: Other disorders of cartilage | Musculoskeletal/ skin | 116 |
| M840: Malunion of fracture | Musculoskeletal/ skin | 108 |
| H653: Chronic mucoid otitis media | Neurological | 11354 |
| H91: Other hearing loss | Neurological | 2490 |
| R568: Other and unspecified convulsions | Neurological | 2426 |
| G409: Epilepsy unspecified | Neurological | 1654 |
| G403: Generalized idiopathic epilepsy and epileptic syndromes | Neurological | 803 |
| G80: Cerebral palsy | Neurological | 802 |
| H900: Conductive hearing loss bilateral | Neurological | 779 |
| G43: Migraine | Neurological | 691 |
| H654: Other chronic nonsuppurative otitis media | Neurological | 642 |
| G93: Other disorders of brain | Neurological | 596 |
| G51: Facial nerve disorders | Neurological | 351 |
| G401: Localization-related (focal)(partial) symptomatic epilepsy and epileptic syndromes with simple partial seizures | Neurological | 336 |
| G479: Sleep disorder unspecified | Neurological | 332 |
| G81: Hemiplegia | Neurological | 322 |
| H740: Tympanosclerosis | Neurological | 300 |
| H47: Other disorders of optic [2nd] nerve and visual pathways | Neurological | 297 |
| Q02: Microcephaly | Neurological | 280 |
| G41: Status epilepticus | Neurological | 272 |
| H663: Other chronic suppurative otitis media | Neurological | 269 |
| H905: Sensorineural hearing loss unspecified | Neurological | 221 |
| J45: Asthma | Respiratory | 18528 |
| G473: Sleep apnoea | Respiratory | 2950 |
| J46: Status asthmaticus | Respiratory | 903 |
| J98: Other respiratory disorders | Respiratory | 764 |
| Q35: Cleft palate | Respiratory | 199 |
| J86: Pyothorax | Respiratory | 159 |
| J47: Bronchiectasis | Respiratory | 134 |
| J69: Pneumonitis due to solids and liquids | Respiratory | 106 |
| J44: Other chronic obstructive pulmonary disease | Respiratory | 84 |
| Q31: Congenital malformations of larynx | Respiratory | 64 |
| Q37: Cleft palate with cleft lip | Respiratory | 56 |
| S27: Injury of other and unspecified intrathoracic organs | Respiratory | 56 |
| E84: Cystic fibrosis | Respiratory | 50 |
| Q32: Congenital malformations of trachea and bronchus | Respiratory | 45 |
| Z930: Tracheostomy status | Respiratory | 42 |
| J81: Pulmonary oedema | Respiratory | 30 |
| J84: Other interstitial pulmonary diseases | Respiratory | 24 |
| Q30: Congenital malformations of nose | Respiratory | 24 |
| P27: Chronic respiratory disease originating in the perinatal period | Respiratory | 22 |
| Z430: Attention to tracheostomy | Respiratory | 22 |
|  |  |  |
| **Age 11 to 15** | | |
|  |  |  |
| D69: Purpura and other haemorrhagic conditions | Cancer/ Blood | 904 |
| D70: Agranulocytosis | Cancer/ Blood | 510 |
| Z512: Other chemotherapy | Cancer/ Blood | 432 |
| D64: Other anaemias | Cancer/ Blood | 422 |
| Z85: Personal history of malignant neoplasm | Cancer/ Blood | 394 |
| D50: Iron deficiency anaemia | Cancer/ Blood | 338 |
| Y433: Other antineoplastic drugs | Cancer/ Blood | 228 |
| Z860: Personal history of other neoplasms | Cancer/ Blood | 225 |
| Z862: Personal history of diseases of the blood and blood-forming organs and certain disorders involving the immune mechanism | Cancer/ Blood | 223 |
| Z923: Personal history of irradiation | Cancer/ Blood | 170 |
| D48: Neoplasm of uncertain or unknown behaviour of other and unspecified sites | Cancer/ Blood | 165 |
| D72: Other disorders of white blood cells | Cancer/ Blood | 156 |
| Z08: Follow-up examination after treatment for malignant neoplasms | Cancer/ Blood | 137 |
| D689: Coagulation defect unspecified | Cancer/ Blood | 121 |
| Z511: Chemotherapy session for neoplasm | Cancer/ Blood | 117 |
| D619: Aplastic anaemia unspecified | Cancer/ Blood | 104 |
| D73: Diseases of spleen | Cancer/ Blood | 102 |
| C71: Malignant neoplasm of brain | Cancer/ Blood | 100 |
| C91: Lymphoid leukaemia | Cancer/ Blood | 99 |
| C81: Hodgkin lymphoma | Cancer/ Blood | 91 |
| Z95: Presence of cardiac and vascular implants and grafts | Cardiovascular | 614 |
| Q21: Congenital malformations of cardiac septa | Cardiovascular | 367 |
| I10: Essential (primary) hypertension | Cardiovascular | 358 |
| T827: Infection and inflammatory reaction due to other cardiac and vascular devices implants and grafts | Cardiovascular | 224 |
| T825: Mechanical complication of other cardiac and vascular devices and implants | Cardiovascular | 219 |
| Q27: Other congenital malformations of peripheral vascular system | Cardiovascular | 216 |
| Q23: Congenital malformations of aortic and mitral valves | Cardiovascular | 211 |
| Q25: Congenital malformations of great arteries | Cardiovascular | 192 |
| T828: Other specified complications of cardiac and vascular prosthetic devices implants and grafts | Cardiovascular | 159 |
| Q24: Other congenital malformations of heart | Cardiovascular | 153 |
| I51: Complications and ill-defined descriptions of heart disease | Cardiovascular | 142 |
| I31: Other diseases of pericardium | Cardiovascular | 133 |
| I49: Other cardiac arrhythmias | Cardiovascular | 114 |
| I47: Paroxysmal tachycardia | Cardiovascular | 87 |
| Q22: Congenital malformations of pulmonary and tricuspid valves | Cardiovascular | 86 |
| I15: Secondary hypertension | Cardiovascular | 72 |
| I46: Cardiac arrest | Cardiovascular | 70 |
| I35: Nonrheumatic aortic valve disorders | Cardiovascular | 66 |
| I34: Nonrheumatic mitral valve disorders | Cardiovascular | 63 |
| Q28: Other congenital malformations of circulatory system | Cardiovascular | 61 |
| M00: Pyogenic arthritis | Chronic infections | 121 |
| B94: Sequelae of other and unspecified infectious and parasitic diseases | Chronic infections | 69 |
| A16: Respiratory tuberculosis not confirmed bacteriologically or histologically | Chronic infections | 33 |
| B371: Pulmonary candidiasis | Chronic infections | 28 |
| B67: Echinococcosis | Chronic infections | 14 |
| A15: Respiratory tuberculosis bacteriologically and histologically confirmed | Chronic infections | 13 |
| B377: Candidal sepsis | Chronic infections | 11 |
| A18: Tuberculosis of other organs | Chronic infections | 10 |
| R62: Lack of expected normal physiological development | Codes indicating non-specific chronic condition | 795 |
| Z993: Dependence on wheelchair | Codes indicating non-specific chronic condition | 712 |
| R633: Feeding difficulties and mismanagement | Codes indicating non-specific chronic condition | 402 |
| Z931: Gastrostomy status | Codes indicating non-specific chronic condition | 281 |
| Z431: Attention to gastrostomy | Codes indicating non-specific chronic condition | 134 |
| Z515: Palliative care | Codes indicating non-specific chronic condition | 103 |
| Z755: Holiday relief care | Codes indicating non-specific chronic condition | 25 |
| Z915: Personal history of self-harm | Mental health/ behavioural | 9481 |
| F41: Other anxiety disorders | Mental health/ behavioural | 7227 |
| X60: Intentional self-poisoning by and exposure to nonopioid analgesics antipyretics and antirheumatics | Mental health/ behavioural | 6916 |
| F84: Pervasive developmental disorders | Mental health/ behavioural | 5214 |
| F32: Depressive episode | Mental health/ behavioural | 4646 |
| F90: Hyperkinetic disorders | Mental health/ behavioural | 3891 |
| X78: Intentional self-harm by sharp object | Mental health/ behavioural | 2899 |
| F17: Mental and behavioural disorders due to use of tobacco | Mental health/ behavioural | 2452 |
| F81: Specific developmental disorders of scholastic skills | Mental health/ behavioural | 2307 |
| X61: Intentional self-poisoning by and exposure to antiepileptic sedative-hypnotic antiparkinsonism and psychotropic drugs not elsewhere classified | Mental health/ behavioural | 2300 |
| X64: Intentional self-poisoning by and exposure to other and unspecified drugs medicaments and biological substances | Mental health/ behavioural | 2168 |
| F50: Eating disorders | Mental health/ behavioural | 1556 |
| F40: Phobic anxiety disorders | Mental health/ behavioural | 1460 |
| F10: Mental and behavioural disorders due to use of alcohol | Mental health/ behavioural | 1405 |
| X62: Intentional self-poisoning by and exposure to narcotics and psychodysleptics [hallucinogens] not elsewhere classified | Mental health/ behavioural | 1355 |
| F89: Unspecified disorder of psychological development | Mental health/ behavioural | 951 |
| F79: Unspecified mental retardation | Mental health/ behavioural | 796 |
| F12: Mental and behavioural disorders due to use of cannabinoids | Mental health/ behavioural | 706 |
| F91: Conduct disorders | Mental health/ behavioural | 584 |
| F43: Reaction to severe stress and adjustment disorders | Mental health/ behavioural | 552 |
| E66: Obesity | Metabolic/ endocrine/ digestive/ renal/ genitourinary | 2216 |
| E10: Type 1 diabetes mellitus | Metabolic/ endocrine/ digestive/ renal/ genitourinary | 1717 |
| K297: Gastritis unspecified | Metabolic/ endocrine/ digestive/ renal/ genitourinary | 1284 |
| K90: Intestinal malabsorption | Metabolic/ endocrine/ digestive/ renal/ genitourinary | 966 |
| K50: Crohn disease [regional enteritis] | Metabolic/ endocrine/ digestive/ renal/ genitourinary | 758 |
| N32: Other disorders of bladder | Metabolic/ endocrine/ digestive/ renal/ genitourinary | 675 |
| N12: Tubulo-interstitial nephritis not specified as acute or chronic | Metabolic/ endocrine/ digestive/ renal/ genitourinary | 656 |
| K66: Other disorders of peritoneum | Metabolic/ endocrine/ digestive/ renal/ genitourinary | 640 |
| Z904: Acquired absence of other parts of digestive tract | Metabolic/ endocrine/ digestive/ renal/ genitourinary | 628 |
| K295: Chronic gastritis unspecified | Metabolic/ endocrine/ digestive/ renal/ genitourinary | 561 |
| K52: Other noninfective gastroenteritis and colitis | Metabolic/ endocrine/ digestive/ renal/ genitourinary | 516 |
| K20: Oesophagitis | Metabolic/ endocrine/ digestive/ renal/ genitourinary | 509 |
| K51: Ulcerative colitis | Metabolic/ endocrine/ digestive/ renal/ genitourinary | 478 |
| K76: Other diseases of liver | Metabolic/ endocrine/ digestive/ renal/ genitourinary | 438 |
| N13: Obstructive and reflux uropathy | Metabolic/ endocrine/ digestive/ renal/ genitourinary | 430 |
| N28: Other disorders of kidney and ureter not elsewhere classified | Metabolic/ endocrine/ digestive/ renal/ genitourinary | 406 |
| E83: Disorders of mineral metabolism | Metabolic/ endocrine/ digestive/ renal/ genitourinary | 394 |
| K22: Other diseases of oesophagus | Metabolic/ endocrine/ digestive/ renal/ genitourinary | 350 |
| K80: Cholelithiasis | Metabolic/ endocrine/ digestive/ renal/ genitourinary | 328 |
| E230: Hypopituitarism | Metabolic/ endocrine/ digestive/ renal/ genitourinary | 288 |
| M54: Dorsalgia | Musculoskeletal/ skin | 2374 |
| M41: Scoliosis | Musculoskeletal/ skin | 2043 |
| M35: Other systemic involvement of connective tissue | Musculoskeletal/ skin | 943 |
| M89: Other disorders of bone | Musculoskeletal/ skin | 689 |
| M93: Other osteochondropathies | Musculoskeletal/ skin | 606 |
| M94: Other disorders of cartilage | Musculoskeletal/ skin | 519 |
| M08: Juvenile arthritis | Musculoskeletal/ skin | 516 |
| M62: Other disorders of muscle | Musculoskeletal/ skin | 488 |
| L90: Atrophic disorders of skin | Musculoskeletal/ skin | 453 |
| M92: Other juvenile osteochondrosis | Musculoskeletal/ skin | 388 |
| Q74: Other congenital malformations of limb(s) | Musculoskeletal/ skin | 374 |
| L40: Psoriasis | Musculoskeletal/ skin | 352 |
| M85: Other disorders of bone density and structure | Musculoskeletal/ skin | 316 |
| M43: Other deforming dorsopathies | Musculoskeletal/ skin | 267 |
| L92: Granulomatous disorders of skin and subcutaneous tissue | Musculoskeletal/ skin | 261 |
| Q658: Other congenital deformities of hip | Musculoskeletal/ skin | 219 |
| M841: Nonunion of fracture [pseudarthrosis] | Musculoskeletal/ skin | 186 |
| Q796: Ehlers-Danlos syndrome | Musculoskeletal/ skin | 179 |
| S32: Fracture of lumbar spine and pelvis | Musculoskeletal/ skin | 179 |
| M13: Other arthritis | Musculoskeletal/ skin | 167 |
| G43: Migraine | Neurological | 2397 |
| R568: Other and unspecified convulsions | Neurological | 2296 |
| G409: Epilepsy unspecified | Neurological | 1179 |
| G93: Other disorders of brain | Neurological | 1089 |
| H91: Other hearing loss | Neurological | 971 |
| G403: Generalized idiopathic epilepsy and epileptic syndromes | Neurological | 760 |
| H653: Chronic mucoid otitis media | Neurological | 651 |
| H47: Other disorders of optic [2nd] nerve and visual pathways | Neurological | 530 |
| G479: Sleep disorder unspecified | Neurological | 430 |
| G51: Facial nerve disorders | Neurological | 401 |
| G80: Cerebral palsy | Neurological | 386 |
| G401: Localization-related (focal)(partial) symptomatic epilepsy and epileptic syndromes with simple partial seizures | Neurological | 379 |
| G44: Other headache syndromes | Neurological | 290 |
| G81: Hemiplegia | Neurological | 266 |
| H18: Other disorders of cornea | Neurological | 253 |
| H663: Other chronic suppurative otitis media | Neurological | 249 |
| H900: Conductive hearing loss bilateral | Neurological | 239 |
| S06: Intracranial injury | Neurological | 235 |
| H740: Tympanosclerosis | Neurological | 215 |
| Q99: Other chromosome abnormalities not elsewhere classified | Neurological | 207 |
| J45: Asthma | Respiratory | 13849 |
| G473: Sleep apnoea | Respiratory | 1055 |
| J98: Other respiratory disorders | Respiratory | 779 |
| J46: Status asthmaticus | Respiratory | 329 |
| S27: Injury of other and unspecified intrathoracic organs | Respiratory | 159 |
| J47: Bronchiectasis | Respiratory | 143 |
| J69: Pneumonitis due to solids and liquids | Respiratory | 115 |
| Q35: Cleft palate | Respiratory | 67 |
| J86: Pyothorax | Respiratory | 58 |
| J43: Emphysema | Respiratory | 50 |
| J81: Pulmonary oedema | Respiratory | 45 |
| J44: Other chronic obstructive pulmonary disease | Respiratory | 43 |
| Q31: Congenital malformations of larynx | Respiratory | 39 |
| Z930: Tracheostomy status | Respiratory | 35 |
| J84: Other interstitial pulmonary diseases | Respiratory | 32 |
| J80: Adult respiratory distress syndrome | Respiratory | 30 |
| J961: Chronic respiratory failure | Respiratory | 29 |
| Q32: Congenital malformations of trachea and bronchus | Respiratory | 29 |
| E84: Cystic fibrosis | Respiratory | 28 |
| J42: Unspecified chronic bronchitis | Respiratory | 20 |

# Table S7. Cumulative incidence of being admitted to hospital and having any record indicating any CHC and each sub-type before age 16 in the sensitivity analyses

| Condition (cohort) | Age | | | | | | | | | | | | | | | | |
| --- | --- | --- | --- | --- | --- | --- | --- | --- | --- | --- | --- | --- | --- | --- | --- | --- | --- |
|  | 0 | 1 | 2 | 3 | 4 | 5 | 6 | 7 | 8 | 9 | 10 | 11 | 12 | 13 | 14 | 15 | 16 |
|  |  |  |  |  |  |  |  |  |  |  |  |  |  |  |  |  |  |
| **Any chronic health condition** | | | | | | | | | | | | | | | | | |
| **2002/3** | | | | | | | | | | | | | | | | | |
| 1. HES birth record only | 3.2% | 6.0% | 7.9% | 9.2% | 10.4% | 11.6% | 12.7% | 13.5% | 14.2% | 14.8% | 15.3% | 15.9% | 16.5% | 17.1% | 17.9% | 19.0% | 20.0% |
| 2. Birth & HES <5 | 4.7% | 9.7% | 13.0% | 15.3% | 17.5% | 19.6% | 21.1% | 22.3% | 23.2% | 24.0% | 24.8% | 25.5% | 26.3% | 27.2% | 28.2% | 29.5% | 30.8% |
| 3. Birth & HES <16 | 3.6% | 7.3% | 9.7% | 11.5% | 13.1% | 14.7% | 16.0% | 17.1% | 18.0% | 18.8% | 19.5% | 20.2% | 21.0% | 21.9% | 22.9% | 24.3% | 25.7% |
| 4. Birth & NPD | 3.3% | 6.7% | 9.1% | 10.8% | 12.3% | 13.9% | 15.3% | 16.4% | 17.2% | 18.0% | 18.7% | 19.4% | 20.2% | 21.1% | 22.1% | 23.5% | 24.8% |
| 5. Birth & HES <5 & NPD | 4.5% | 9.3% | 12.7% | 15.1% | 17.4% | 19.6% | 21.2% | 22.4% | 23.4% | 24.2% | 25.0% | 25.8% | 26.6% | 27.5% | 28.6% | 29.9% | 31.2% |
| 6. Birth & HES <5 & HES 5 to <16 | 4.6% | 9.7% | 13.1% | 15.6% | 17.9% | 20.3% | 22.0% | 23.3% | 24.3% | 25.2% | 26.0% | 26.9% | 27.8% | 28.7% | 29.9% | 31.3% | 32.8% |
| 7. Birth & HES <5 & HES 5 to <16 & NPD | 4.6% | 9.6% | 13.1% | 15.6% | 17.9% | 20.2% | 22.0% | 23.3% | 24.3% | 25.2% | 26.0% | 26.9% | 27.8% | 28.7% | 29.9% | 31.4% | 32.8% |
|  |  |  |  |  |  |  |  |  |  |  |  |  |  |  |  |  |  |
| **2003/4** |  |  |  |  |  |  |  |  |  |  |  |  |  |  |  |  |  |
| 1. HES birth record only | 3.2% | 6.2% | 8.2% | 9.7% | 11.1% | 12.4% | 13.6% | 14.6% | 15.4% | 16.0% | 16.6% | 17.3% | 17.9% | 18.7% | 19.6% | 20.8% | 22.0% |
| 2. Birth & HES <5 | 4.3% | 9.0% | 12.1% | 14.3% | 16.4% | 18.5% | 20.1% | 21.3% | 22.2% | 23.0% | 23.8% | 24.5% | 25.3% | 26.2% | 27.3% | 28.6% | 29.9% |
| 3. Birth & HES <16 | 3.4% | 6.9% | 9.3% | 11.0% | 12.6% | 14.2% | 15.6% | 16.8% | 17.6% | 18.4% | 19.1% | 19.9% | 20.6% | 21.5% | 22.6% | 24.0% | 25.4% |
| 4. Birth & NPD | 3.1% | 6.4% | 8.7% | 10.4% | 12.0% | 13.6% | 14.9% | 16.1% | 16.9% | 17.7% | 18.4% | 19.2% | 19.9% | 20.8% | 21.9% | 23.2% | 24.6% |
| 5. Birth & HES <5 & NPD | 4.1% | 8.7% | 11.8% | 14.1% | 16.3% | 18.5% | 20.1% | 21.3% | 22.3% | 23.1% | 23.9% | 24.7% | 25.5% | 26.4% | 27.5% | 28.9% | 30.2% |
| 6. Birth & HES <5 & HES 5 to <16 | 4.3% | 9.0% | 12.2% | 14.6% | 16.8% | 19.1% | 20.8% | 22.2% | 23.2% | 24.1% | 24.9% | 25.8% | 26.6% | 27.6% | 28.8% | 30.3% | 31.7% |
| 7. Birth & HES <5 & HES 5 to <16 & NPD | 4.2% | 8.9% | 12.1% | 14.5% | 16.8% | 19.0% | 20.8% | 22.1% | 23.2% | 24.0% | 24.9% | 25.7% | 26.6% | 27.6% | 28.8% | 30.3% | 31.7% |
|  |  |  |  |  |  |  |  |  |  |  |  |  |  |  |  |  |  |
| **2004/5** |  |  |  |  |  |  |  |  |  |  |  |  |  |  |  |  |  |
| 1. HES birth record only | 3.2% | 6.3% | 8.4% | 9.9% | 11.4% | 12.8% | 14.1% | 15.1% | 15.9% | 16.6% | 17.2% | 17.9% | 18.5% | 19.3% | 20.4% | 21.5% |  |
| 2. Birth & HES <5 | 4.1% | 8.5% | 11.6% | 13.7% | 15.8% | 17.8% | 19.4% | 20.6% | 21.5% | 22.3% | 23.1% | 23.8% | 24.6% | 25.5% | 26.7% | 27.9% |  |
| 3. Birth & HES <16 | 3.4% | 6.9% | 9.3% | 11.0% | 12.7% | 14.3% | 15.8% | 16.9% | 17.7% | 18.5% | 19.2% | 20.0% | 20.8% | 21.7% | 22.8% | 24.1% |  |
| 4. Birth & NPD | 3.1% | 6.4% | 8.7% | 10.4% | 12.1% | 13.7% | 15.1% | 16.2% | 17.1% | 17.8% | 18.6% | 19.3% | 20.0% | 20.9% | 22.1% | 23.4% |  |
| 5. Birth & HES <5 & NPD | 3.9% | 8.3% | 11.3% | 13.5% | 15.7% | 17.8% | 19.4% | 20.6% | 21.6% | 22.4% | 23.2% | 24.0% | 24.8% | 25.7% | 26.9% | 28.2% |  |
| 6. Birth & HES <5 & HES 5 to <16 | 4.1% | 8.6% | 11.7% | 14.0% | 16.2% | 18.4% | 20.2% | 21.5% | 22.5% | 23.4% | 24.3% | 25.1% | 26.0% | 27.0% | 28.3% | 29.7% |  |
| 7. Birth & HES <5 & HES 5 to <16 & NPD | 4.0% | 8.5% | 11.7% | 13.9% | 16.2% | 18.4% | 20.2% | 21.5% | 22.5% | 23.4% | 24.2% | 25.1% | 26.0% | 27.0% | 28.3% | 29.7% |  |
|  |  |  |  |  |  |  |  |  |  |  |  |  |  |  |  |  |  |
| **2005/6** |  |  |  |  |  |  |  |  |  |  |  |  |  |  |  |  |  |
| 1. HES birth record only | 3.3% | 6.5% | 8.6% | 10.1% | 11.6% | 13.0% | 14.3% | 15.3% | 16.1% | 16.8% | 17.4% | 18.1% | 18.8% | 19.6% | 20.6% |  |  |
| 2. Birth & HES <5 | 4.1% | 8.4% | 11.2% | 13.2% | 15.2% | 17.1% | 18.6% | 19.8% | 20.7% | 21.5% | 22.2% | 23.0% | 23.8% | 24.8% | 25.8% |  |  |
| 3. Birth & HES <16 | 3.5% | 7.1% | 9.4% | 11.1% | 12.7% | 14.4% | 15.8% | 16.9% | 17.8% | 18.5% | 19.3% | 20.0% | 20.8% | 21.8% | 22.8% |  |  |
| 4. Birth & NPD | 3.2% | 6.6% | 8.8% | 10.5% | 12.1% | 13.7% | 15.1% | 16.2% | 17.1% | 17.9% | 18.6% | 19.3% | 20.1% | 21.0% | 22.1% |  |  |
| 5. Birth & HES <5 & NPD | 3.9% | 8.2% | 10.9% | 13.0% | 15.0% | 17.0% | 18.6% | 19.8% | 20.7% | 21.5% | 22.3% | 23.1% | 23.9% | 24.9% | 25.9% |  |  |
| 6. Birth & HES <5 & HES 5 to <16 | 4.0% | 8.5% | 11.4% | 13.5% | 15.7% | 17.8% | 19.5% | 20.8% | 21.9% | 22.8% | 23.6% | 24.5% | 25.4% | 26.4% | 27.6% |  |  |
| 7. Birth & HES <5 & HES 5 to <16 & NPD | 4.0% | 8.4% | 11.3% | 13.5% | 15.6% | 17.7% | 19.4% | 20.8% | 21.8% | 22.7% | 23.5% | 24.4% | 25.3% | 26.4% | 27.6% |  |  |
|  |  |  |  |  |  |  |  |  |  |  |  |  |  |  |  |  |  |
| **2006/7** |  |  |  |  |  |  |  |  |  |  |  |  |  |  |  |  |  |
| 1. HES birth record only | 3.4% | 6.5% | 8.6% | 10.1% | 11.6% | 13.0% | 14.3% | 15.3% | 16.1% | 16.8% | 17.5% | 18.2% | 18.9% | 19.7% |  |  |  |
| 2. Birth & HES <5 | 4.0% | 8.0% | 10.6% | 12.5% | 14.4% | 16.2% | 17.8% | 18.9% | 19.8% | 20.6% | 21.3% | 22.1% | 22.9% | 23.8% |  |  |  |
| 3. Birth & HES <16 | 3.5% | 7.0% | 9.3% | 11.0% | 12.6% | 14.2% | 15.7% | 16.8% | 17.7% | 18.4% | 19.2% | 19.9% | 20.7% | 21.6% |  |  |  |
| 4. Birth & NPD | 3.2% | 6.5% | 8.7% | 10.4% | 12.1% | 13.6% | 15.1% | 16.2% | 17.0% | 17.8% | 18.5% | 19.3% | 20.1% | 21.0% |  |  |  |
| 5. Birth & HES <5 & NPD | 3.7% | 7.7% | 10.3% | 12.3% | 14.3% | 16.1% | 17.7% | 18.9% | 19.8% | 20.6% | 21.4% | 22.2% | 23.0% | 23.9% |  |  |  |
| 6. Birth & HES <5 & HES 5 to <16 | 4.0% | 8.1% | 10.9% | 13.0% | 15.1% | 17.1% | 18.9% | 20.2% | 21.2% | 22.1% | 23.0% | 23.9% | 24.9% | 25.9% |  |  |  |
| 7. Birth & HES <5 & HES 5 to <16 & NPD | 3.9% | 8.0% | 10.8% | 12.9% | 15.0% | 17.0% | 18.8% | 20.1% | 21.2% | 22.1% | 22.9% | 23.8% | 24.8% | 25.8% |  |  |  |
|  |  |  |  |  |  |  |  |  |  |  |  |  |  |  |  |  |  |
| **2007/8** |  |  |  |  |  |  |  |  |  |  |  |  |  |  |  |  |  |
| 1. HES birth record only | 3.5% | 6.6% | 8.6% | 10.1% | 11.6% | 13.1% | 14.3% | 15.3% | 16.0% | 16.7% | 17.4% | 18.1% | 18.7% |  |  |  |  |
| 2. Birth & HES <5 | 4.0% | 7.9% | 10.5% | 12.4% | 14.3% | 16.1% | 17.6% | 18.7% | 19.6% | 20.4% | 21.2% | 22.0% | 22.7% |  |  |  |  |
| 3. Birth & HES <16 | 3.7% | 7.2% | 9.5% | 11.3% | 13.0% | 14.6% | 16.1% | 17.1% | 18.0% | 18.8% | 19.6% | 20.4% | 21.1% |  |  |  |  |
| 4. Birth & NPD | 3.4% | 6.8% | 9.0% | 10.7% | 12.4% | 14.1% | 15.5% | 16.6% | 17.5% | 18.3% | 19.0% | 19.8% | 20.5% |  |  |  |  |
| 5. Birth & HES <5 & NPD | 3.8% | 7.7% | 10.3% | 12.2% | 14.2% | 16.1% | 17.6% | 18.8% | 19.7% | 20.5% | 21.3% | 22.1% | 22.9% |  |  |  |  |
| 6. Birth & HES <5 & HES 5 to <16 | 4.1% | 8.2% | 10.9% | 13.1% | 15.2% | 17.3% | 19.0% | 20.3% | 21.4% | 22.3% | 23.3% | 24.2% | 25.0% |  |  |  |  |
| 7. Birth & HES <5 & HES 5 to <16 & NPD | 4.0% | 8.1% | 10.9% | 13.0% | 15.1% | 17.2% | 19.0% | 20.3% | 21.4% | 22.3% | 23.3% | 24.2% | 25.0% |  |  |  |  |
|  |  |  |  |  |  |  |  |  |  |  |  |  |  |  |  |  |  |
| **2008/9** |  |  |  |  |  |  |  |  |  |  |  |  |  |  |  |  |  |
| 1. HES birth record only | 3.6% | 6.9% | 9.0% | 10.5% | 12.1% | 13.5% | 14.8% | 15.8% | 16.6% | 17.4% | 18.1% | 18.8% |  |  |  |  |  |
| 2. Birth & HES <5 | 4.0% | 7.9% | 10.4% | 12.3% | 14.1% | 15.8% | 17.3% | 18.4% | 19.3% | 20.1% | 20.9% | 21.7% |  |  |  |  |  |
| 3. Birth & HES <16 | 3.8% | 7.3% | 9.7% | 11.3% | 13.1% | 14.6% | 16.1% | 17.2% | 18.1% | 18.9% | 19.7% | 20.4% |  |  |  |  |  |
| 4. Birth & NPD | 3.4% | 6.9% | 9.1% | 10.8% | 12.5% | 14.1% | 15.5% | 16.6% | 17.5% | 18.3% | 19.1% | 19.9% |  |  |  |  |  |
| 5. Birth & HES <5 & NPD | 3.8% | 7.7% | 10.2% | 12.1% | 14.0% | 15.8% | 17.3% | 18.5% | 19.4% | 20.3% | 21.1% | 21.9% |  |  |  |  |  |
| 6. Birth & HES <5 & HES 5 to <16 | 4.1% | 8.3% | 11.1% | 13.1% | 15.2% | 17.2% | 19.0% | 20.4% | 21.5% | 22.5% | 23.5% | 24.4% |  |  |  |  |  |
| 7. Birth & HES <5 & HES 5 to <16 & NPD | 4.0% | 8.2% | 11.0% | 13.0% | 15.1% | 17.1% | 19.0% | 20.4% | 21.5% | 22.5% | 23.5% | 24.4% |  |  |  |  |  |
|  |  |  |  |  |  |  |  |  |  |  |  |  |  |  |  |  |  |
| **2009/10** |  |  |  |  |  |  |  |  |  |  |  |  |  |  |  |  |  |
| 1. HES birth record only | 3.8% | 7.2% | 9.3% | 10.8% | 12.3% | 13.8% | 15.1% | 16.2% | 17.0% | 17.8% | 18.5% |  |  |  |  |  |  |
| 2. Birth & HES <5 | 4.2% | 8.1% | 10.5% | 12.2% | 13.9% | 15.7% | 17.1% | 18.3% | 19.2% | 20.0% | 20.8% |  |  |  |  |  |  |
| 3. Birth & HES <16 | 3.9% | 7.6% | 9.8% | 11.4% | 13.0% | 14.6% | 16.1% | 17.2% | 18.1% | 19.0% | 19.7% |  |  |  |  |  |  |
| 4. Birth & NPD | 3.6% | 7.1% | 9.2% | 10.8% | 12.4% | 14.0% | 15.5% | 16.6% | 17.5% | 18.3% | 19.1% |  |  |  |  |  |  |
| 5. Birth & HES <5 & NPD | 4.0% | 7.9% | 10.2% | 12.0% | 13.8% | 15.6% | 17.1% | 18.3% | 19.3% | 20.1% | 20.9% |  |  |  |  |  |  |
| 6. Birth & HES <5 & HES 5 to <16 | 4.4% | 8.7% | 11.3% | 13.3% | 15.3% | 17.4% | 19.3% | 20.7% | 21.9% | 23.0% | 23.9% |  |  |  |  |  |  |
| 7. Birth & HES <5 & HES 5 to <16 & NPD | 4.3% | 8.6% | 11.2% | 13.2% | 15.2% | 17.3% | 19.2% | 20.6% | 21.8% | 22.9% | 23.9% |  |  |  |  |  |  |
|  |  |  |  |  |  |  |  |  |  |  |  |  |  |  |  |  |  |
| **2010/11** |  |  |  |  |  |  |  |  |  |  |  |  |  |  |  |  |  |
| 1. HES birth record only | 3.8% | 7.1% | 8.8% | 10.3% | 11.8% | 13.4% | 14.7% | 15.8% | 16.6% | 17.4% |  |  |  |  |  |  |  |
| 2. Birth & HES <5 | 4.2% | 7.9% | 9.9% | 11.6% | 13.3% | 15.1% | 16.6% | 17.7% | 18.6% | 19.4% |  |  |  |  |  |  |  |
| 3. Birth & HES <16 | 3.9% | 7.5% | 9.4% | 10.9% | 12.6% | 14.2% | 15.7% | 16.8% | 17.8% | 18.6% |  |  |  |  |  |  |  |
| 4. Birth & NPD | 3.6% | 7.0% | 8.8% | 10.3% | 11.9% | 13.5% | 15.0% | 16.1% | 17.0% | 17.8% |  |  |  |  |  |  |  |
| 5. Birth & HES <5 & NPD | 3.9% | 7.7% | 9.7% | 11.4% | 13.2% | 15.0% | 16.6% | 17.7% | 18.7% | 19.5% |  |  |  |  |  |  |  |
| 6. Birth & HES <5 & HES 5 to <16 | 4.5% | 8.7% | 11.0% | 12.9% | 15.1% | 17.2% | 19.2% | 20.8% | 22.0% | 23.1% |  |  |  |  |  |  |  |
| 7. Birth & HES <5 & HES 5 to <16 & NPD | 4.4% | 8.6% | 10.8% | 12.8% | 15.0% | 17.1% | 19.1% | 20.7% | 22.0% | 23.0% |  |  |  |  |  |  |  |
|  |  |  |  |  |  |  |  |  |  |  |  |  |  |  |  |  |  |
| **2011/12** |  |  |  |  |  |  |  |  |  |  |  |  |  |  |  |  |  |
| 1. HES birth record only | 3.9% | 6.7% | 8.2% | 9.7% | 11.3% | 12.8% | 14.2% | 15.3% | 16.1% |  |  |  |  |  |  |  |  |
| 2. Birth & HES <5 | 4.2% | 7.3% | 9.0% | 10.7% | 12.5% | 14.2% | 15.7% | 16.9% | 17.7% |  |  |  |  |  |  |  |  |
| 3. Birth & HES <16 | 4.0% | 7.0% | 8.6% | 10.2% | 11.9% | 13.5% | 15.0% | 16.2% | 17.1% |  |  |  |  |  |  |  |  |
| 4. Birth & NPD | 3.7% | 6.5% | 8.1% | 9.6% | 11.3% | 12.9% | 14.4% | 15.6% | 16.4% |  |  |  |  |  |  |  |  |
| 5. Birth & HES <5 & NPD | 4.0% | 7.1% | 8.8% | 10.5% | 12.4% | 14.2% | 15.7% | 16.9% | 17.8% |  |  |  |  |  |  |  |  |
| 6. Birth & HES <5 & HES 5 to <16 | 4.7% | 8.3% | 10.4% | 12.4% | 14.7% | 16.9% | 19.0% | 20.8% | 22.0% |  |  |  |  |  |  |  |  |
| 7. Birth & HES <5 & HES 5 to <16 & NPD | 4.6% | 8.2% | 10.3% | 12.3% | 14.6% | 16.8% | 19.0% | 20.7% | 21.9% |  |  |  |  |  |  |  |  |
|  |  |  |  |  |  |  |  |  |  |  |  |  |  |  |  |  |  |
| **Cancer / blood** | | | | | | | | | | | | | | | | | |
| **2002/3 & 2003/4** | | | | | | | | | | | | | | | | | |
| 1. HES birth record only | 0.1% | 0.3% | 0.4% | 0.5% | 0.6% | 0.6% | 0.7% | 0.8% | 0.9% | 1.0% | 1.1% | 1.2% | 1.3% | 1.3% | 1.4% | 1.4% | 1.5% |
| 2. Birth & HES <5 | 0.1% | 0.4% | 0.6% | 0.8% | 0.9% | 1.0% | 1.2% | 1.3% | 1.4% | 1.6% | 1.7% | 1.8% | 1.9% | 2.0% | 2.1% | 2.1% | 2.2% |
| 3. Birth & HES <16 | 0.1% | 0.3% | 0.5% | 0.6% | 0.7% | 0.8% | 0.9% | 1.0% | 1.1% | 1.2% | 1.4% | 1.5% | 1.5% | 1.6% | 1.7% | 1.8% | 1.9% |
| 4. Birth & NPD | 0.1% | 0.3% | 0.4% | 0.5% | 0.6% | 0.7% | 0.8% | 1.0% | 1.1% | 1.2% | 1.3% | 1.4% | 1.5% | 1.6% | 1.6% | 1.7% | 1.8% |
| 5. Birth & HES <5 & NPD | 0.1% | 0.4% | 0.6% | 0.7% | 0.9% | 1.0% | 1.2% | 1.3% | 1.4% | 1.5% | 1.7% | 1.8% | 1.9% | 2.0% | 2.1% | 2.1% | 2.2% |
| 6. Birth & HES <5 & HES 5 to <16 | 0.1% | 0.4% | 0.6% | 0.7% | 0.9% | 1.1% | 1.2% | 1.3% | 1.5% | 1.6% | 1.8% | 1.9% | 2.0% | 2.1% | 2.2% | 2.3% | 2.4% |
| 7. Birth & HES <5 & HES 5 to <16 & NPD | 0.1% | 0.4% | 0.6% | 0.7% | 0.9% | 1.1% | 1.2% | 1.3% | 1.5% | 1.6% | 1.8% | 1.9% | 2.0% | 2.1% | 2.2% | 2.3% | 2.4% |
|  |  |  |  |  |  |  |  |  |  |  |  |  |  |  |  |  |  |
| **2004/5 & 2005/6** |  |  |  |  |  |  |  |  |  |  |  |  |  |  |  |  |  |
| 1. HES birth record only | 0.1% | 0.3% | 0.4% | 0.5% | 0.7% | 0.8% | 0.9% | 1.0% | 1.1% | 1.2% | 1.3% | 1.4% | 1.4% | 1.5% | 1.6% |  |  |
| 2. Birth & HES <5 | 0.1% | 0.4% | 0.6% | 0.7% | 0.9% | 1.1% | 1.2% | 1.4% | 1.5% | 1.6% | 1.7% | 1.8% | 1.8% | 1.9% | 2.0% |  |  |
| 3. Birth & HES <16 | 0.1% | 0.3% | 0.5% | 0.6% | 0.7% | 0.9% | 1.0% | 1.1% | 1.3% | 1.4% | 1.5% | 1.5% | 1.6% | 1.7% | 1.7% |  |  |
| 4. Birth & NPD | 0.1% | 0.3% | 0.4% | 0.5% | 0.7% | 0.8% | 0.9% | 1.1% | 1.2% | 1.3% | 1.4% | 1.5% | 1.5% | 1.6% | 1.7% |  |  |
| 5. Birth & HES <5 & NPD | 0.1% | 0.4% | 0.5% | 0.7% | 0.9% | 1.0% | 1.2% | 1.3% | 1.5% | 1.6% | 1.7% | 1.8% | 1.8% | 1.9% | 2.0% |  |  |
| 6. Birth & HES <5 & HES 5 to <16 | 0.1% | 0.4% | 0.6% | 0.7% | 0.9% | 1.1% | 1.3% | 1.4% | 1.6% | 1.7% | 1.8% | 1.9% | 1.9% | 2.0% | 2.1% |  |  |
| 7. Birth & HES <5 & HES 5 to <16 & NPD | 0.1% | 0.4% | 0.6% | 0.7% | 0.9% | 1.1% | 1.2% | 1.4% | 1.5% | 1.7% | 1.8% | 1.9% | 1.9% | 2.0% | 2.1% |  |  |
|  |  |  |  |  |  |  |  |  |  |  |  |  |  |  |  |  |  |
| **2006/7 & 2007/8** |  |  |  |  |  |  |  |  |  |  |  |  |  |  |  |  |  |
| 1. HES birth record only | 0.1% | 0.3% | 0.4% | 0.6% | 0.7% | 0.9% | 1.0% | 1.1% | 1.2% | 1.3% | 1.3% | 1.4% | 1.5% |  |  |  |  |
| 2. Birth & HES <5 | 0.1% | 0.4% | 0.6% | 0.7% | 0.9% | 1.1% | 1.3% | 1.4% | 1.5% | 1.6% | 1.6% | 1.7% | 1.8% |  |  |  |  |
| 3. Birth & HES <16 | 0.1% | 0.3% | 0.5% | 0.6% | 0.8% | 1.0% | 1.1% | 1.3% | 1.4% | 1.4% | 1.5% | 1.6% | 1.6% |  |  |  |  |
| 4. Birth & NPD | 0.1% | 0.3% | 0.4% | 0.6% | 0.8% | 0.9% | 1.1% | 1.2% | 1.3% | 1.4% | 1.4% | 1.5% | 1.6% |  |  |  |  |
| 5. Birth & HES <5 & NPD | 0.1% | 0.3% | 0.5% | 0.7% | 0.9% | 1.1% | 1.2% | 1.4% | 1.5% | 1.6% | 1.6% | 1.7% | 1.8% |  |  |  |  |
| 6. Birth & HES <5 & HES 5 to <16 | 0.1% | 0.4% | 0.6% | 0.7% | 1.0% | 1.2% | 1.3% | 1.5% | 1.6% | 1.7% | 1.8% | 1.9% | 2.0% |  |  |  |  |
| 7. Birth & HES <5 & HES 5 to <16 & NPD | 0.1% | 0.4% | 0.6% | 0.7% | 0.9% | 1.1% | 1.3% | 1.5% | 1.6% | 1.7% | 1.8% | 1.9% | 1.9% |  |  |  |  |
|  |  |  |  |  |  |  |  |  |  |  |  |  |  |  |  |  |  |
| **2008/9 & 2009/10** |  |  |  |  |  |  |  |  |  |  |  |  |  |  |  |  |  |
| 1. HES birth record only | 0.1% | 0.4% | 0.6% | 0.8% | 0.9% | 1.1% | 1.2% | 1.3% | 1.4% | 1.4% | 1.5% |  |  |  |  |  |  |
| 2. Birth & HES <5 | 0.1% | 0.5% | 0.7% | 0.9% | 1.1% | 1.3% | 1.4% | 1.5% | 1.6% | 1.6% | 1.7% |  |  |  |  |  |  |
| 3. Birth & HES <16 | 0.1% | 0.5% | 0.7% | 0.8% | 1.0% | 1.2% | 1.3% | 1.4% | 1.5% | 1.5% | 1.6% |  |  |  |  |  |  |
| 4. Birth & NPD | 0.1% | 0.4% | 0.6% | 0.8% | 0.9% | 1.1% | 1.2% | 1.3% | 1.4% | 1.5% | 1.5% |  |  |  |  |  |  |
| 5. Birth & HES <5 & NPD | 0.1% | 0.5% | 0.7% | 0.8% | 1.0% | 1.2% | 1.4% | 1.5% | 1.5% | 1.6% | 1.7% |  |  |  |  |  |  |
| 6. Birth & HES <5 & HES 5 to <16 | 0.1% | 0.5% | 0.8% | 1.0% | 1.2% | 1.4% | 1.6% | 1.7% | 1.8% | 1.9% | 1.9% |  |  |  |  |  |  |
| 7. Birth & HES <5 & HES 5 to <16 & NPD | 0.1% | 0.5% | 0.7% | 0.9% | 1.2% | 1.4% | 1.5% | 1.7% | 1.7% | 1.8% | 1.9% |  |  |  |  |  |  |
|  |  |  |  |  |  |  |  |  |  |  |  |  |  |  |  |  |  |
| **2010/11 & 2011/12** |  |  |  |  |  |  |  |  |  |  |  |  |  |  |  |  |  |
| 1. HES birth record only | 0.1% | 0.6% | 0.8% | 1.0% | 1.1% | 1.2% | 1.3% | 1.4% | 1.5% |  |  |  |  |  |  |  |  |
| 2. Birth & HES <5 | 0.1% | 0.6% | 0.9% | 1.1% | 1.2% | 1.4% | 1.5% | 1.6% | 1.7% |  |  |  |  |  |  |  |  |
| 3. Birth & HES <16 | 0.1% | 0.6% | 0.8% | 1.0% | 1.2% | 1.3% | 1.4% | 1.5% | 1.6% |  |  |  |  |  |  |  |  |
| 4. Birth & NPD | 0.1% | 0.5% | 0.8% | 0.9% | 1.1% | 1.2% | 1.3% | 1.4% | 1.5% |  |  |  |  |  |  |  |  |
| 5. Birth & HES <5 & NPD | 0.1% | 0.6% | 0.8% | 1.0% | 1.2% | 1.3% | 1.5% | 1.6% | 1.6% |  |  |  |  |  |  |  |  |
| 6. Birth & HES <5 & HES 5 to <16 | 0.2% | 0.7% | 1.0% | 1.2% | 1.4% | 1.6% | 1.7% | 1.9% | 2.0% |  |  |  |  |  |  |  |  |
| 7. Birth & HES <5 & HES 5 to <16 & NPD | 0.1% | 0.7% | 1.0% | 1.2% | 1.4% | 1.6% | 1.7% | 1.9% | 2.0% |  |  |  |  |  |  |  |  |
|  |  |  |  |  |  |  |  |  |  |  |  |  |  |  |  |  |  |
| **Cardiovascular** | | | | | | | | | | | | | | | | | |
| **2002/3 & 2003/4** | | | | | | | | | | | | | | | | | |
| 1. HES birth record only |  |  |  |  |  |  |  |  |  |  |  |  |  |  |  |  |  |
| 2. Birth & HES <5 | 0.7% | 0.9% | 1.0% | 1.1% | 1.1% | 1.1% | 1.2% | 1.2% | 1.2% | 1.2% | 1.3% | 1.3% | 1.3% | 1.3% | 1.4% | 1.4% | 1.5% |
| 3. Birth & HES <16 | 1.0% | 1.4% | 1.5% | 1.6% | 1.7% | 1.7% | 1.8% | 1.8% | 1.8% | 1.9% | 1.9% | 1.9% | 2.0% | 2.0% | 2.1% | 2.1% | 2.2% |
| 4. Birth & NPD | 0.8% | 1.1% | 1.2% | 1.2% | 1.3% | 1.3% | 1.3% | 1.4% | 1.4% | 1.4% | 1.5% | 1.5% | 1.5% | 1.6% | 1.6% | 1.7% | 1.7% |
| 5. Birth & HES <5 & NPD | 0.7% | 0.9% | 1.0% | 1.0% | 1.1% | 1.1% | 1.2% | 1.2% | 1.2% | 1.3% | 1.3% | 1.3% | 1.3% | 1.4% | 1.4% | 1.5% | 1.5% |
| 6. Birth & HES <5 & HES 5 to <16 | 0.9% | 1.3% | 1.4% | 1.4% | 1.5% | 1.6% | 1.6% | 1.7% | 1.7% | 1.7% | 1.8% | 1.8% | 1.8% | 1.9% | 1.9% | 2.0% | 2.1% |
| 7. Birth & HES <5 & HES 5 to <16 & NPD | 1.0% | 1.4% | 1.5% | 1.5% | 1.6% | 1.7% | 1.7% | 1.8% | 1.8% | 1.8% | 1.9% | 1.9% | 2.0% | 2.0% | 2.1% | 2.1% | 2.2% |
|  |  |  |  |  |  |  |  |  |  |  |  |  |  |  |  |  |  |
| **2004/5 & 2005/6** |  |  |  |  |  |  |  |  |  |  |  |  |  |  |  |  |  |
| 1. HES birth record only | 0.8% | 1.0% | 1.1% | 1.1% | 1.2% | 1.2% | 1.3% | 1.3% | 1.3% | 1.3% | 1.4% | 1.4% | 1.4% | 1.5% | 1.5% |  |  |
| 2. Birth & HES <5 | 1.0% | 1.3% | 1.4% | 1.5% | 1.6% | 1.6% | 1.7% | 1.7% | 1.7% | 1.8% | 1.8% | 1.8% | 1.9% | 1.9% | 2.0% |  |  |
| 3. Birth & HES <16 | 0.8% | 1.1% | 1.2% | 1.2% | 1.3% | 1.3% | 1.4% | 1.4% | 1.4% | 1.5% | 1.5% | 1.5% | 1.6% | 1.6% | 1.6% |  |  |
| 4. Birth & NPD | 0.7% | 0.9% | 1.0% | 1.1% | 1.1% | 1.2% | 1.2% | 1.2% | 1.3% | 1.3% | 1.3% | 1.3% | 1.4% | 1.4% | 1.5% |  |  |
| 5. Birth & HES <5 & NPD | 0.9% | 1.2% | 1.3% | 1.3% | 1.4% | 1.5% | 1.5% | 1.5% | 1.6% | 1.6% | 1.7% | 1.7% | 1.7% | 1.8% | 1.8% |  |  |
| 6. Birth & HES <5 & HES 5 to <16 | 0.9% | 1.3% | 1.4% | 1.4% | 1.5% | 1.6% | 1.6% | 1.7% | 1.7% | 1.7% | 1.8% | 1.8% | 1.9% | 1.9% | 2.0% |  |  |
| 7. Birth & HES <5 & HES 5 to <16 & NPD | 0.9% | 1.2% | 1.3% | 1.4% | 1.5% | 1.5% | 1.6% | 1.6% | 1.7% | 1.7% | 1.7% | 1.8% | 1.8% | 1.9% | 1.9% |  |  |
|  |  |  |  |  |  |  |  |  |  |  |  |  |  |  |  |  |  |
| **2006/7 & 2007/8** |  |  |  |  |  |  |  |  |  |  |  |  |  |  |  |  |  |
| 1. HES birth record only | 0.9% | 1.1% | 1.2% | 1.2% | 1.3% | 1.3% | 1.4% | 1.4% | 1.4% | 1.5% | 1.5% | 1.5% | 1.6% |  |  |  |  |
| 2. Birth & HES <5 | 1.0% | 1.3% | 1.4% | 1.5% | 1.6% | 1.6% | 1.6% | 1.7% | 1.7% | 1.8% | 1.8% | 1.8% | 1.9% |  |  |  |  |
| 3. Birth & HES <16 | 0.9% | 1.2% | 1.3% | 1.3% | 1.4% | 1.4% | 1.5% | 1.5% | 1.5% | 1.6% | 1.6% | 1.6% | 1.7% |  |  |  |  |
| 4. Birth & NPD | 0.8% | 1.0% | 1.1% | 1.2% | 1.2% | 1.3% | 1.3% | 1.3% | 1.4% | 1.4% | 1.4% | 1.5% | 1.5% |  |  |  |  |
| 5. Birth & HES <5 & NPD | 0.9% | 1.2% | 1.3% | 1.3% | 1.4% | 1.5% | 1.5% | 1.5% | 1.6% | 1.6% | 1.6% | 1.7% | 1.7% |  |  |  |  |
| 6. Birth & HES <5 & HES 5 to <16 | 1.0% | 1.3% | 1.4% | 1.5% | 1.6% | 1.6% | 1.7% | 1.7% | 1.8% | 1.8% | 1.8% | 1.9% | 1.9% |  |  |  |  |
| 7. Birth & HES <5 & HES 5 to <16 & NPD | 0.9% | 1.3% | 1.4% | 1.4% | 1.5% | 1.6% | 1.6% | 1.7% | 1.7% | 1.7% | 1.8% | 1.8% | 1.9% |  |  |  |  |
|  |  |  |  |  |  |  |  |  |  |  |  |  |  |  |  |  |  |
| **2008/9 & 2009/10** |  |  |  |  |  |  |  |  |  |  |  |  |  |  |  |  |  |
| 1. HES birth record only | 1.0% | 1.3% | 1.4% | 1.4% | 1.5% | 1.5% | 1.6% | 1.6% | 1.6% | 1.7% | 1.7% |  |  |  |  |  |  |
| 2. Birth & HES <5 | 1.1% | 1.5% | 1.5% | 1.6% | 1.7% | 1.7% | 1.8% | 1.8% | 1.8% | 1.9% | 1.9% |  |  |  |  |  |  |
| 3. Birth & HES <16 | 1.0% | 1.4% | 1.4% | 1.5% | 1.6% | 1.6% | 1.6% | 1.7% | 1.7% | 1.8% | 1.8% |  |  |  |  |  |  |
| 4. Birth & NPD | 0.9% | 1.2% | 1.3% | 1.3% | 1.4% | 1.4% | 1.5% | 1.5% | 1.5% | 1.6% | 1.6% |  |  |  |  |  |  |
| 5. Birth & HES <5 & NPD | 1.0% | 1.3% | 1.4% | 1.5% | 1.5% | 1.6% | 1.6% | 1.7% | 1.7% | 1.7% | 1.8% |  |  |  |  |  |  |
| 6. Birth & HES <5 & HES 5 to <16 | 1.2% | 1.5% | 1.6% | 1.7% | 1.8% | 1.8% | 1.9% | 1.9% | 2.0% | 2.0% | 2.1% |  |  |  |  |  |  |
| 7. Birth & HES <5 & HES 5 to <16 & NPD | 1.1% | 1.5% | 1.6% | 1.7% | 1.7% | 1.8% | 1.8% | 1.9% | 1.9% | 2.0% | 2.0% |  |  |  |  |  |  |
|  |  |  |  |  |  |  |  |  |  |  |  |  |  |  |  |  |  |
| **2010/11 & 2011/12** |  |  |  |  |  |  |  |  |  |  |  |  |  |  |  |  |  |
| 1. HES birth record only | 1.1% | 1.4% | 1.5% | 1.6% | 1.6% | 1.7% | 1.7% | 1.8% | 1.8% |  |  |  |  |  |  |  |  |
| 2. Birth & HES <5 | 1.2% | 1.5% | 1.6% | 1.7% | 1.8% | 1.8% | 1.9% | 1.9% | 2.0% |  |  |  |  |  |  |  |  |
| 3. Birth & HES <16 | 1.1% | 1.5% | 1.6% | 1.6% | 1.7% | 1.7% | 1.8% | 1.8% | 1.9% |  |  |  |  |  |  |  |  |
| 4. Birth & NPD | 1.0% | 1.3% | 1.4% | 1.4% | 1.5% | 1.5% | 1.6% | 1.6% | 1.7% |  |  |  |  |  |  |  |  |
| 5. Birth & HES <5 & NPD | 1.1% | 1.4% | 1.5% | 1.6% | 1.6% | 1.7% | 1.7% | 1.8% | 1.8% |  |  |  |  |  |  |  |  |
| 6. Birth & HES <5 & HES 5 to <16 | 1.3% | 1.7% | 1.9% | 1.9% | 2.0% | 2.1% | 2.2% | 2.2% | 2.3% |  |  |  |  |  |  |  |  |
| 7. Birth & HES <5 & HES 5 to <16 & NPD | 1.3% | 1.7% | 1.8% | 1.9% | 2.0% | 2.1% | 2.1% | 2.2% | 2.2% |  |  |  |  |  |  |  |  |
|  |  |  |  |  |  |  |  |  |  |  |  |  |  |  |  |  |  |
| **Mental health / behavioural** | | | | | | | | | | | | | | | | | |
| **2002/3 & 2003/4** | | | | | | | | | | | | | | | | | |
| 1. HES birth record only | ~0.0% | ~0.0% | 0.1% | 0.2% | 0.3% | 0.4% | 0.5% | 0.6% | 0.8% | 0.9% | 1.1% | 1.3% | 1.5% | 1.8% | 2.3% | 3.1% | 3.9% |
| 2. Birth & HES <5 | ~0.0% | ~0.0% | 0.1% | 0.3% | 0.4% | 0.6% | 0.8% | 1.0% | 1.2% | 1.4% | 1.7% | 2.0% | 2.3% | 2.7% | 3.4% | 4.4% | 5.4% |
| 3. Birth & HES <16 | ~0.0% | ~0.0% | 0.1% | 0.2% | 0.3% | 0.5% | 0.6% | 0.8% | 0.9% | 1.1% | 1.3% | 1.6% | 1.8% | 2.2% | 2.9% | 3.8% | 4.8% |
| 4. Birth & NPD | ~0.0% | ~0.0% | 0.1% | 0.2% | 0.3% | 0.4% | 0.6% | 0.7% | 0.9% | 1.1% | 1.3% | 1.5% | 1.8% | 2.2% | 2.8% | 3.8% | 4.8% |
| 5. Birth & HES <5 & NPD | ~0.0% | ~0.0% | 0.1% | 0.3% | 0.4% | 0.6% | 0.8% | 1.0% | 1.2% | 1.5% | 1.7% | 2.0% | 2.3% | 2.8% | 3.5% | 4.5% | 5.6% |
| 6. Birth & HES <5 & HES 5 to <16 | ~0.0% | ~0.0% | 0.1% | 0.3% | 0.4% | 0.6% | 0.8% | 1.1% | 1.3% | 1.6% | 1.9% | 2.2% | 2.5% | 3.0% | 3.7% | 4.8% | 6.0% |
| 7. Birth & HES <5 & HES 5 to <16 & NPD | ~0.0% | ~0.0% | 0.1% | 0.3% | 0.4% | 0.6% | 0.8% | 1.1% | 1.3% | 1.6% | 1.9% | 2.2% | 2.5% | 3.0% | 3.7% | 4.8% | 6.0% |
|  |  |  |  |  |  |  |  |  |  |  |  |  |  |  |  |  |  |
| **2004/5 & 2005/6** |  |  |  |  |  |  |  |  |  |  |  |  |  |  |  |  |  |
| 1. HES birth record only | ~0.0% | ~0.0% | 0.1% | 0.2% | 0.3% | 0.5% | 0.6% | 0.8% | 1.0% | 1.2% | 1.4% | 1.6% | 1.9% | 2.3% | 2.9% |  |  |
| 2. Birth & HES <5 | ~0.0% | 0.1% | 0.1% | 0.3% | 0.4% | 0.7% | 0.9% | 1.1% | 1.3% | 1.6% | 1.9% | 2.1% | 2.5% | 3.0% | 3.7% |  |  |
| 3. Birth & HES <16 | ~0.0% | ~0.0% | 0.1% | 0.2% | 0.4% | 0.5% | 0.7% | 0.9% | 1.1% | 1.4% | 1.6% | 1.8% | 2.1% | 2.6% | 3.3% |  |  |
| 4. Birth & NPD | ~0.0% | ~0.0% | 0.1% | 0.2% | 0.3% | 0.5% | 0.7% | 0.9% | 1.1% | 1.3% | 1.5% | 1.8% | 2.1% | 2.5% | 3.2% |  |  |
| 5. Birth & HES <5 & NPD | ~0.0% | ~0.0% | 0.1% | 0.3% | 0.4% | 0.6% | 0.9% | 1.1% | 1.3% | 1.6% | 1.9% | 2.2% | 2.5% | 3.0% | 3.7% |  |  |
| 6. Birth & HES <5 & HES 5 to <16 | ~0.0% | ~0.0% | 0.1% | 0.3% | 0.5% | 0.7% | 0.9% | 1.2% | 1.5% | 1.8% | 2.1% | 2.4% | 2.7% | 3.3% | 4.1% |  |  |
| 7. Birth & HES <5 & HES 5 to <16 & NPD | ~0.0% | ~0.0% | 0.1% | 0.3% | 0.5% | 0.7% | 0.9% | 1.2% | 1.4% | 1.7% | 2.1% | 2.4% | 2.7% | 3.3% | 4.1% |  |  |
|  |  |  |  |  |  |  |  |  |  |  |  |  |  |  |  |  |  |
| **2006/7 & 2007/8** |  |  |  |  |  |  |  |  |  |  |  |  |  |  |  |  |  |
| 1. HES birth record only | ~0.0% | ~0.0% | 0.1% | 0.2% | 0.4% | 0.6% | 0.8% | 1.0% | 1.2% | 1.4% | 1.7% | 1.9% | 2.2% |  |  |  |  |
| 2. Birth & HES <5 | ~0.0% | ~0.0% | 0.1% | 0.3% | 0.5% | 0.8% | 1.0% | 1.3% | 1.5% | 1.8% | 2.1% | 2.4% | 2.7% |  |  |  |  |
| 3. Birth & HES <16 | ~0.0% | ~0.0% | 0.1% | 0.3% | 0.5% | 0.7% | 0.9% | 1.1% | 1.4% | 1.6% | 1.9% | 2.2% | 2.5% |  |  |  |  |
| 4. Birth & NPD | ~0.0% | ~0.0% | 0.1% | 0.3% | 0.4% | 0.7% | 0.9% | 1.1% | 1.4% | 1.6% | 1.9% | 2.1% | 2.4% |  |  |  |  |
| 5. Birth & HES <5 & NPD | ~0.0% | ~0.0% | 0.1% | 0.3% | 0.5% | 0.8% | 1.0% | 1.3% | 1.6% | 1.8% | 2.1% | 2.4% | 2.8% |  |  |  |  |
| 6. Birth & HES <5 & HES 5 to <16 | ~0.0% | ~0.0% | 0.1% | 0.3% | 0.6% | 0.9% | 1.2% | 1.5% | 1.8% | 2.1% | 2.4% | 2.7% | 3.1% |  |  |  |  |
| 7. Birth & HES <5 & HES 5 to <16 & NPD | ~0.0% | ~0.0% | 0.1% | 0.3% | 0.6% | 0.9% | 1.2% | 1.4% | 1.7% | 2.1% | 2.4% | 2.7% | 3.1% |  |  |  |  |
|  |  |  |  |  |  |  |  |  |  |  |  |  |  |  |  |  |  |
| **2008/9 & 2009/10** |  |  |  |  |  |  |  |  |  |  |  |  |  |  |  |  |  |
| 1. HES birth record only | ~0.0% | 0.1% | 0.2% | 0.4% | 0.6% | 0.9% | 1.1% | 1.3% | 1.6% | 1.9% | 2.1% |  |  |  |  |  |  |
| 2. Birth & HES <5 | ~0.0% | 0.1% | 0.2% | 0.4% | 0.7% | 1.0% | 1.3% | 1.6% | 1.8% | 2.2% | 2.5% |  |  |  |  |  |  |
| 3. Birth & HES <16 | ~0.0% | 0.1% | 0.2% | 0.4% | 0.7% | 0.9% | 1.2% | 1.4% | 1.7% | 2.0% | 2.3% |  |  |  |  |  |  |
| 4. Birth & NPD | ~0.0% | ~0.0% | 0.2% | 0.4% | 0.7% | 0.9% | 1.2% | 1.4% | 1.7% | 2.0% | 2.3% |  |  |  |  |  |  |
| 5. Birth & HES <5 & NPD | ~0.0% | 0.1% | 0.2% | 0.4% | 0.7% | 1.0% | 1.3% | 1.6% | 1.9% | 2.2% | 2.5% |  |  |  |  |  |  |
| 6. Birth & HES <5 & HES 5 to <16 | ~0.0% | 0.1% | 0.2% | 0.5% | 0.9% | 1.2% | 1.5% | 1.9% | 2.2% | 2.6% | 3.0% |  |  |  |  |  |  |
| 7. Birth & HES <5 & HES 5 to <16 & NPD | ~0.0% | 0.1% | 0.2% | 0.5% | 0.8% | 1.2% | 1.5% | 1.9% | 2.2% | 2.6% | 3.0% |  |  |  |  |  |  |
|  |  |  |  |  |  |  |  |  |  |  |  |  |  |  |  |  |  |
| **2010/11 & 2011/12** |  |  |  |  |  |  |  |  |  |  |  |  |  |  |  |  |  |
| 1. HES birth record only | ~0.0% | 0.1% | 0.2% | 0.5% | 0.8% | 1.0% | 1.3% | 1.6% | 1.9% |  |  |  |  |  |  |  |  |
| 2. Birth & HES <5 | ~0.0% | 0.1% | 0.3% | 0.6% | 0.9% | 1.2% | 1.5% | 1.8% | 2.1% |  |  |  |  |  |  |  |  |
| 3. Birth & HES <16 | ~0.0% | 0.1% | 0.3% | 0.5% | 0.8% | 1.1% | 1.4% | 1.7% | 2.0% |  |  |  |  |  |  |  |  |
| 4. Birth & NPD | ~0.0% | 0.1% | 0.2% | 0.5% | 0.8% | 1.1% | 1.4% | 1.7% | 1.9% |  |  |  |  |  |  |  |  |
| 5. Birth & HES <5 & NPD | ~0.0% | 0.1% | 0.3% | 0.5% | 0.9% | 1.2% | 1.5% | 1.8% | 2.1% |  |  |  |  |  |  |  |  |
| 6. Birth & HES <5 & HES 5 to <16 | ~0.0% | 0.1% | 0.3% | 0.7% | 1.1% | 1.5% | 2.0% | 2.4% | 2.8% |  |  |  |  |  |  |  |  |
| 7. Birth & HES <5 & HES 5 to <16 & NPD | ~0.0% | 0.1% | 0.3% | 0.7% | 1.1% | 1.5% | 1.9% | 2.4% | 2.8% |  |  |  |  |  |  |  |  |
|  |  |  |  |  |  |  |  |  |  |  |  |  |  |  |  |  |  |
| **Metabolic, &c.** | | | | | | | | | | | | | | | | | |
| **2002/3 & 2003/4** | | | | | | | | | | | | | | | | | |
| 1. HES birth record only | 1.1% | 2.6% | 3.4% | 3.9% | 4.2% | 4.4% | 4.6% | 4.8% | 5.0% | 5.2% | 5.3% | 5.5% | 5.7% | 5.9% | 6.1% | 6.4% | 6.7% |
| 2. Birth & HES <5 | 1.6% | 4.0% | 5.4% | 6.1% | 6.6% | 7.0% | 7.3% | 7.5% | 7.8% | 8.0% | 8.2% | 8.4% | 8.6% | 8.9% | 9.2% | 9.5% | 9.9% |
| 3. Birth & HES <16 | 1.3% | 3.0% | 4.1% | 4.6% | 5.0% | 5.3% | 5.5% | 5.8% | 6.0% | 6.2% | 6.4% | 6.6% | 6.8% | 7.1% | 7.4% | 7.7% | 8.1% |
| 4. Birth & NPD | 1.2% | 2.8% | 3.9% | 4.4% | 4.7% | 5.0% | 5.3% | 5.5% | 5.7% | 5.9% | 6.1% | 6.3% | 6.5% | 6.8% | 7.1% | 7.4% | 7.7% |
| 5. Birth & HES <5 & NPD | 1.6% | 3.9% | 5.3% | 6.0% | 6.5% | 6.9% | 7.2% | 7.5% | 7.8% | 8.0% | 8.2% | 8.4% | 8.7% | 8.9% | 9.2% | 9.6% | 10.0% |
| 6. Birth & HES <5 & HES 5 to <16 | 1.6% | 4.0% | 5.5% | 6.2% | 6.7% | 7.1% | 7.4% | 7.7% | 8.0% | 8.3% | 8.5% | 8.7% | 9.0% | 9.3% | 9.6% | 10.0% | 10.4% |
| 7. Birth & HES <5 & HES 5 to <16 & NPD | 1.6% | 4.0% | 5.5% | 6.2% | 6.7% | 7.1% | 7.4% | 7.7% | 8.0% | 8.3% | 8.5% | 8.7% | 9.0% | 9.3% | 9.6% | 10.0% | 10.4% |
|  |  |  |  |  |  |  |  |  |  |  |  |  |  |  |  |  |  |
| **2004/5 & 2005/6** |  |  |  |  |  |  |  |  |  |  |  |  |  |  |  |  |  |
| 1. HES birth record only | 1.2% | 2.8% | 3.8% | 4.2% | 4.6% | 4.8% | 5.1% | 5.3% | 5.4% | 5.6% | 5.8% | 5.9% | 6.1% | 6.4% | 6.7% |  |  |
| 2. Birth & HES <5 | 1.5% | 3.8% | 5.1% | 5.7% | 6.2% | 6.6% | 6.8% | 7.1% | 7.3% | 7.4% | 7.7% | 7.9% | 8.1% | 8.4% | 8.7% |  |  |
| 3. Birth & HES <16 | 1.3% | 3.1% | 4.2% | 4.7% | 5.1% | 5.4% | 5.6% | 5.9% | 6.0% | 6.2% | 6.4% | 6.6% | 6.8% | 7.1% | 7.4% |  |  |
| 4. Birth & NPD | 1.2% | 2.9% | 3.9% | 4.4% | 4.8% | 5.1% | 5.4% | 5.6% | 5.8% | 5.9% | 6.1% | 6.3% | 6.6% | 6.8% | 7.1% |  |  |
| 5. Birth & HES <5 & NPD | 1.4% | 3.7% | 5.0% | 5.6% | 6.1% | 6.5% | 6.8% | 7.0% | 7.2% | 7.4% | 7.6% | 7.8% | 8.1% | 8.4% | 8.7% |  |  |
| 6. Birth & HES <5 & HES 5 to <16 | 1.5% | 3.8% | 5.2% | 5.8% | 6.3% | 6.7% | 7.1% | 7.3% | 7.5% | 7.7% | 8.0% | 8.2% | 8.5% | 8.8% | 9.1% |  |  |
| 7. Birth & HES <5 & HES 5 to <16 & NPD | 1.5% | 3.8% | 5.2% | 5.8% | 6.3% | 6.7% | 7.1% | 7.3% | 7.5% | 7.7% | 7.9% | 8.2% | 8.5% | 8.8% | 9.1% |  |  |
|  |  |  |  |  |  |  |  |  |  |  |  |  |  |  |  |  |  |
| **2006/7 & 2007/8** |  |  |  |  |  |  |  |  |  |  |  |  |  |  |  |  |  |
| 1. HES birth record only | 1.2% | 2.9% | 3.8% | 4.3% | 4.6% | 4.8% | 5.0% | 5.2% | 5.3% | 5.5% | 5.7% | 5.9% | 6.1% |  |  |  |  |
| 2. Birth & HES <5 | 1.4% | 3.5% | 4.7% | 5.3% | 5.7% | 6.0% | 6.2% | 6.4% | 6.6% | 6.8% | 7.0% | 7.3% | 7.5% |  |  |  |  |
| 3. Birth & HES <16 | 1.3% | 3.1% | 4.2% | 4.8% | 5.1% | 5.4% | 5.6% | 5.7% | 5.9% | 6.1% | 6.3% | 6.6% | 6.8% |  |  |  |  |
| 4. Birth & NPD | 1.2% | 3.0% | 4.0% | 4.5% | 4.9% | 5.1% | 5.3% | 5.5% | 5.7% | 5.9% | 6.1% | 6.3% | 6.6% |  |  |  |  |
| 5. Birth & HES <5 & NPD | 1.4% | 3.4% | 4.7% | 5.3% | 5.7% | 6.0% | 6.2% | 6.4% | 6.6% | 6.8% | 7.0% | 7.2% | 7.5% |  |  |  |  |
| 6. Birth & HES <5 & HES 5 to <16 | 1.4% | 3.6% | 4.9% | 5.6% | 6.0% | 6.4% | 6.6% | 6.8% | 7.0% | 7.2% | 7.5% | 7.8% | 8.1% |  |  |  |  |
| 7. Birth & HES <5 & HES 5 to <16 & NPD | 1.4% | 3.6% | 4.9% | 5.5% | 6.0% | 6.3% | 6.6% | 6.8% | 7.0% | 7.2% | 7.5% | 7.7% | 8.0% |  |  |  |  |
|  |  |  |  |  |  |  |  |  |  |  |  |  |  |  |  |  |  |
| **2008/9 & 2009/10** |  |  |  |  |  |  |  |  |  |  |  |  |  |  |  |  |  |
| 1. HES birth record only | 1.3% | 3.1% | 4.1% | 4.5% | 4.7% | 4.8% | 5.0% | 5.2% | 5.4% | 5.6% | 5.8% |  |  |  |  |  |  |
| 2. Birth & HES <5 | 1.4% | 3.6% | 4.7% | 5.1% | 5.4% | 5.6% | 5.8% | 6.0% | 6.2% | 6.4% | 6.6% |  |  |  |  |  |  |
| 3. Birth & HES <16 | 1.3% | 3.3% | 4.4% | 4.8% | 5.0% | 5.2% | 5.4% | 5.6% | 5.8% | 6.0% | 6.2% |  |  |  |  |  |  |
| 4. Birth & NPD | 1.2% | 3.1% | 4.2% | 4.5% | 4.8% | 4.9% | 5.1% | 5.3% | 5.5% | 5.7% | 6.0% |  |  |  |  |  |  |
| 5. Birth & HES <5 & NPD | 1.4% | 3.5% | 4.6% | 5.1% | 5.3% | 5.5% | 5.7% | 5.9% | 6.1% | 6.4% | 6.6% |  |  |  |  |  |  |
| 6. Birth & HES <5 & HES 5 to <16 | 1.5% | 3.8% | 5.1% | 5.5% | 5.8% | 6.0% | 6.3% | 6.5% | 6.8% | 7.1% | 7.3% |  |  |  |  |  |  |
| 7. Birth & HES <5 & HES 5 to <16 & NPD | 1.5% | 3.8% | 5.0% | 5.5% | 5.8% | 6.0% | 6.3% | 6.5% | 6.8% | 7.0% | 7.3% |  |  |  |  |  |  |
|  |  |  |  |  |  |  |  |  |  |  |  |  |  |  |  |  |  |
| **2010/11 & 2011/12** |  |  |  |  |  |  |  |  |  |  |  |  |  |  |  |  |  |
| 1. HES birth record only | 1.3% | 2.6% | 3.0% | 3.3% | 3.5% | 3.7% | 3.8% | 4.1% | 4.3% |  |  |  |  |  |  |  |  |
| 2. Birth & HES <5 | 1.4% | 2.9% | 3.4% | 3.6% | 3.9% | 4.1% | 4.3% | 4.5% | 4.7% |  |  |  |  |  |  |  |  |
| 3. Birth & HES <16 | 1.3% | 2.8% | 3.2% | 3.5% | 3.7% | 3.9% | 4.1% | 4.3% | 4.5% |  |  |  |  |  |  |  |  |
| 4. Birth & NPD | 1.2% | 2.6% | 3.0% | 3.2% | 3.4% | 3.6% | 3.9% | 4.1% | 4.3% |  |  |  |  |  |  |  |  |
| 5. Birth & HES <5 & NPD | 1.3% | 2.8% | 3.3% | 3.6% | 3.8% | 4.0% | 4.2% | 4.5% | 4.7% |  |  |  |  |  |  |  |  |
| 6. Birth & HES <5 & HES 5 to <16 | 1.5% | 3.3% | 3.9% | 4.2% | 4.4% | 4.7% | 5.0% | 5.3% | 5.7% |  |  |  |  |  |  |  |  |
| 7. Birth & HES <5 & HES 5 to <16 & NPD | 1.5% | 3.2% | 3.8% | 4.1% | 4.4% | 4.7% | 5.0% | 5.3% | 5.6% |  |  |  |  |  |  |  |  |
|  |  |  |  |  |  |  |  |  |  |  |  |  |  |  |  |  |  |
| **Musculoskeletal / skin** | | | | | | | | | | | | | | | | | |
| **2002/3 & 2003/4** | | | | | | | | | | | | | | | | | |
| 1. HES birth record only | 0.3% | 0.5% | 0.6% | 0.7% | 0.8% | 0.9% | 1.0% | 1.1% | 1.2% | 1.3% | 1.4% | 1.5% | 1.6% | 1.8% | 2.0% | 2.2% | 2.5% |
| 2. Birth & HES <5 | 0.4% | 0.7% | 1.0% | 1.1% | 1.3% | 1.4% | 1.6% | 1.7% | 1.8% | 1.9% | 2.1% | 2.2% | 2.4% | 2.6% | 2.9% | 3.2% | 3.5% |
| 3. Birth & HES <16 | 0.3% | 0.6% | 0.7% | 0.9% | 1.0% | 1.1% | 1.2% | 1.3% | 1.4% | 1.5% | 1.7% | 1.8% | 2.0% | 2.2% | 2.4% | 2.7% | 3.0% |
| 4. Birth & NPD | 0.3% | 0.5% | 0.7% | 0.8% | 0.9% | 1.0% | 1.1% | 1.2% | 1.4% | 1.5% | 1.6% | 1.7% | 1.9% | 2.1% | 2.3% | 2.6% | 2.9% |
| 5. Birth & HES <5 & NPD | 0.4% | 0.7% | 0.9% | 1.1% | 1.3% | 1.4% | 1.6% | 1.7% | 1.8% | 1.9% | 2.1% | 2.2% | 2.4% | 2.7% | 2.9% | 3.3% | 3.6% |
| 6. Birth & HES <5 & HES 5 to <16 | 0.4% | 0.7% | 1.0% | 1.1% | 1.3% | 1.5% | 1.6% | 1.7% | 1.9% | 2.0% | 2.2% | 2.3% | 2.6% | 2.8% | 3.1% | 3.4% | 3.8% |
| 7. Birth & HES <5 & HES 5 to <16 & NPD | 0.4% | 0.7% | 1.0% | 1.1% | 1.3% | 1.5% | 1.6% | 1.7% | 1.9% | 2.0% | 2.2% | 2.3% | 2.6% | 2.8% | 3.1% | 3.4% | 3.8% |
|  |  |  |  |  |  |  |  |  |  |  |  |  |  |  |  |  |  |
| **2004/5 & 2005/6** |  |  |  |  |  |  |  |  |  |  |  |  |  |  |  |  |  |
| 1. HES birth record only | 0.3% | 0.5% | 0.7% | 0.8% | 0.9% | 1.0% | 1.1% | 1.2% | 1.3% | 1.4% | 1.6% | 1.7% | 1.9% | 2.1% | 2.3% |  |  |
| 2. Birth & HES <5 | 0.4% | 0.7% | 0.9% | 1.1% | 1.3% | 1.4% | 1.5% | 1.6% | 1.8% | 1.9% | 2.0% | 2.2% | 2.4% | 2.6% | 2.9% |  |  |
| 3. Birth & HES <16 | 0.3% | 0.6% | 0.8% | 0.9% | 1.0% | 1.2% | 1.3% | 1.4% | 1.5% | 1.6% | 1.7% | 1.9% | 2.1% | 2.3% | 2.5% |  |  |
| 4. Birth & NPD | 0.3% | 0.5% | 0.7% | 0.9% | 1.0% | 1.1% | 1.2% | 1.3% | 1.4% | 1.5% | 1.7% | 1.8% | 2.0% | 2.2% | 2.5% |  |  |
| 5. Birth & HES <5 & NPD | 0.4% | 0.7% | 0.9% | 1.1% | 1.2% | 1.4% | 1.5% | 1.6% | 1.8% | 1.9% | 2.0% | 2.2% | 2.4% | 2.6% | 2.9% |  |  |
| 6. Birth & HES <5 & HES 5 to <16 | 0.4% | 0.7% | 0.9% | 1.1% | 1.3% | 1.4% | 1.6% | 1.7% | 1.9% | 2.0% | 2.1% | 2.3% | 2.5% | 2.8% | 3.1% |  |  |
| 7. Birth & HES <5 & HES 5 to <16 & NPD | 0.4% | 0.7% | 0.9% | 1.1% | 1.3% | 1.4% | 1.6% | 1.7% | 1.8% | 2.0% | 2.1% | 2.3% | 2.5% | 2.8% | 3.1% |  |  |
|  |  |  |  |  |  |  |  |  |  |  |  |  |  |  |  |  |  |
| **2006/7 & 2007/8** |  |  |  |  |  |  |  |  |  |  |  |  |  |  |  |  |  |
| 1. HES birth record only | 0.3% | 0.5% | 0.7% | 0.8% | 1.0% | 1.1% | 1.2% | 1.3% | 1.4% | 1.5% | 1.6% | 1.8% | 1.9% |  |  |  |  |
| 2. Birth & HES <5 | 0.4% | 0.7% | 0.9% | 1.0% | 1.2% | 1.3% | 1.4% | 1.5% | 1.7% | 1.8% | 2.0% | 2.1% | 2.3% |  |  |  |  |
| 3. Birth & HES <16 | 0.3% | 0.6% | 0.8% | 0.9% | 1.0% | 1.2% | 1.3% | 1.4% | 1.5% | 1.6% | 1.8% | 2.0% | 2.1% |  |  |  |  |
| 4. Birth & NPD | 0.3% | 0.5% | 0.7% | 0.9% | 1.0% | 1.1% | 1.2% | 1.3% | 1.5% | 1.6% | 1.7% | 1.9% | 2.1% |  |  |  |  |
| 5. Birth & HES <5 & NPD | 0.3% | 0.6% | 0.8% | 1.0% | 1.2% | 1.3% | 1.4% | 1.5% | 1.7% | 1.8% | 2.0% | 2.1% | 2.3% |  |  |  |  |
| 6. Birth & HES <5 & HES 5 to <16 | 0.4% | 0.7% | 0.9% | 1.1% | 1.2% | 1.4% | 1.5% | 1.7% | 1.8% | 2.0% | 2.2% | 2.4% | 2.6% |  |  |  |  |
| 7. Birth & HES <5 & HES 5 to <16 & NPD | 0.4% | 0.7% | 0.9% | 1.1% | 1.2% | 1.4% | 1.5% | 1.7% | 1.8% | 2.0% | 2.2% | 2.4% | 2.6% |  |  |  |  |
|  |  |  |  |  |  |  |  |  |  |  |  |  |  |  |  |  |  |
| **2008/9 & 2009/10** |  |  |  |  |  |  |  |  |  |  |  |  |  |  |  |  |  |
| 1. HES birth record only | 0.3% | 0.6% | 0.8% | 0.9% | 1.0% | 1.2% | 1.3% | 1.4% | 1.5% | 1.6% | 1.8% |  |  |  |  |  |  |
| 2. Birth & HES <5 | 0.4% | 0.7% | 0.9% | 1.0% | 1.2% | 1.3% | 1.5% | 1.6% | 1.7% | 1.9% | 2.0% |  |  |  |  |  |  |
| 3. Birth & HES <16 | 0.4% | 0.6% | 0.8% | 1.0% | 1.1% | 1.2% | 1.4% | 1.5% | 1.6% | 1.8% | 1.9% |  |  |  |  |  |  |
| 4. Birth & NPD | 0.3% | 0.6% | 0.8% | 0.9% | 1.1% | 1.2% | 1.3% | 1.4% | 1.6% | 1.7% | 1.8% |  |  |  |  |  |  |
| 5. Birth & HES <5 & NPD | 0.4% | 0.6% | 0.8% | 1.0% | 1.2% | 1.3% | 1.4% | 1.6% | 1.7% | 1.9% | 2.0% |  |  |  |  |  |  |
| 6. Birth & HES <5 & HES 5 to <16 | 0.4% | 0.7% | 0.9% | 1.1% | 1.3% | 1.5% | 1.6% | 1.8% | 2.0% | 2.1% | 2.3% |  |  |  |  |  |  |
| 7. Birth & HES <5 & HES 5 to <16 & NPD | 0.4% | 0.7% | 0.9% | 1.1% | 1.3% | 1.5% | 1.6% | 1.8% | 1.9% | 2.1% | 2.3% |  |  |  |  |  |  |
|  |  |  |  |  |  |  |  |  |  |  |  |  |  |  |  |  |  |
| **2010/11 & 2011/12** |  |  |  |  |  |  |  |  |  |  |  |  |  |  |  |  |  |
| 1. HES birth record only | 0.4% | 0.6% | 0.8% | 1.0% | 1.1% | 1.3% | 1.4% | 1.5% | 1.6% |  |  |  |  |  |  |  |  |
| 2. Birth & HES <5 | 0.4% | 0.7% | 0.9% | 1.1% | 1.3% | 1.4% | 1.6% | 1.7% | 1.8% |  |  |  |  |  |  |  |  |
| 3. Birth & HES <16 | 0.4% | 0.7% | 0.9% | 1.0% | 1.2% | 1.3% | 1.5% | 1.6% | 1.7% |  |  |  |  |  |  |  |  |
| 4. Birth & NPD | 0.3% | 0.6% | 0.8% | 1.0% | 1.1% | 1.3% | 1.4% | 1.5% | 1.7% |  |  |  |  |  |  |  |  |
| 5. Birth & HES <5 & NPD | 0.4% | 0.7% | 0.9% | 1.1% | 1.2% | 1.4% | 1.5% | 1.7% | 1.8% |  |  |  |  |  |  |  |  |
| 6. Birth & HES <5 & HES 5 to <16 | 0.4% | 0.8% | 1.1% | 1.3% | 1.5% | 1.7% | 1.9% | 2.1% | 2.3% |  |  |  |  |  |  |  |  |
| 7. Birth & HES <5 & HES 5 to <16 & NPD | 0.4% | 0.8% | 1.1% | 1.3% | 1.5% | 1.7% | 1.9% | 2.1% | 2.2% |  |  |  |  |  |  |  |  |
|  |  |  |  |  |  |  |  |  |  |  |  |  |  |  |  |  |  |
| **Neurological** | | | | | | | | | | | | | | | | | |
| **2002/3 & 2003/4** | | | | | | | | | | | | | | | | | |
| 1. HES birth record only | 0.9% | 1.5% | 2.1% | 2.6% | 3.1% | 3.8% | 4.4% | 4.8% | 5.1% | 5.2% | 5.4% | 5.6% | 5.7% | 5.9% | 6.1% | 6.3% | 6.5% |
| 2. Birth & HES <5 | 1.3% | 2.3% | 3.2% | 4.0% | 4.8% | 6.0% | 6.8% | 7.4% | 7.7% | 8.0% | 8.2% | 8.4% | 8.6% | 8.8% | 9.0% | 9.3% | 9.6% |
| 3. Birth & HES <16 | 1.0% | 1.7% | 2.4% | 3.0% | 3.7% | 4.5% | 5.3% | 5.8% | 6.1% | 6.3% | 6.5% | 6.7% | 6.9% | 7.1% | 7.3% | 7.6% | 7.9% |
| 4. Birth & NPD | 0.9% | 1.5% | 2.2% | 2.8% | 3.4% | 4.3% | 5.0% | 5.5% | 5.8% | 6.0% | 6.2% | 6.4% | 6.6% | 6.8% | 7.0% | 7.3% | 7.5% |
| 5. Birth & HES <5 & NPD | 1.2% | 2.1% | 3.0% | 3.8% | 4.7% | 5.9% | 6.8% | 7.3% | 7.7% | 8.0% | 8.2% | 8.4% | 8.6% | 8.8% | 9.1% | 9.4% | 9.7% |
| 6. Birth & HES <5 & HES 5 to <16 | 1.2% | 2.2% | 3.2% | 4.0% | 4.9% | 6.2% | 7.1% | 7.7% | 8.1% | 8.4% | 8.6% | 8.8% | 9.1% | 9.3% | 9.6% | 9.9% | 10.2% |
| 7. Birth & HES <5 & HES 5 to <16 & NPD | 1.2% | 2.2% | 3.1% | 3.9% | 4.9% | 6.1% | 7.1% | 7.7% | 8.1% | 8.4% | 8.6% | 8.8% | 9.1% | 9.3% | 9.6% | 9.9% | 10.2% |
|  |  |  |  |  |  |  |  |  |  |  |  |  |  |  |  |  |  |
| **2004/5 & 2005/6** |  |  |  |  |  |  |  |  |  |  |  |  |  |  |  |  |  |
| 1. HES birth record only | 1.0% | 1.6% | 2.2% | 2.7% | 3.3% | 3.9% | 4.5% | 5.0% | 5.2% | 5.4% | 5.6% | 5.8% | 6.0% | 6.1% | 6.3% |  |  |
| 2. Birth & HES <5 | 1.2% | 2.1% | 2.8% | 3.5% | 4.4% | 5.3% | 6.0% | 6.5% | 6.9% | 7.1% | 7.3% | 7.5% | 7.7% | 8.0% | 8.2% |  |  |
| 3. Birth & HES <16 | 1.0% | 1.7% | 2.3% | 2.9% | 3.6% | 4.3% | 5.0% | 5.5% | 5.8% | 6.0% | 6.2% | 6.4% | 6.6% | 6.8% | 7.0% |  |  |
| 4. Birth & NPD | 0.9% | 1.5% | 2.2% | 2.7% | 3.4% | 4.1% | 4.8% | 5.2% | 5.5% | 5.8% | 6.0% | 6.1% | 6.3% | 6.6% | 6.8% |  |  |
| 5. Birth & HES <5 & NPD | 1.1% | 1.9% | 2.7% | 3.4% | 4.2% | 5.2% | 6.0% | 6.5% | 6.8% | 7.1% | 7.3% | 7.5% | 7.7% | 8.0% | 8.2% |  |  |
| 6. Birth & HES <5 & HES 5 to <16 | 1.1% | 2.0% | 2.8% | 3.6% | 4.5% | 5.5% | 6.3% | 6.9% | 7.3% | 7.5% | 7.8% | 8.0% | 8.2% | 8.5% | 8.7% |  |  |
| 7. Birth & HES <5 & HES 5 to <16 & NPD | 1.1% | 2.0% | 2.8% | 3.6% | 4.4% | 5.4% | 6.3% | 6.9% | 7.3% | 7.5% | 7.7% | 8.0% | 8.2% | 8.5% | 8.7% |  |  |
|  |  |  |  |  |  |  |  |  |  |  |  |  |  |  |  |  |  |
| **2006/7 & 2007/8** |  |  |  |  |  |  |  |  |  |  |  |  |  |  |  |  |  |
| 1. HES birth record only | 1.1% | 1.7% | 2.3% | 2.7% | 3.3% | 3.9% | 4.5% | 4.9% | 5.2% | 5.4% | 5.6% | 5.7% | 5.9% |  |  |  |  |
| 2. Birth & HES <5 | 1.2% | 2.0% | 2.7% | 3.3% | 4.0% | 4.8% | 5.6% | 6.0% | 6.3% | 6.5% | 6.8% | 6.9% | 7.1% |  |  |  |  |
| 3. Birth & HES <16 | 1.1% | 1.8% | 2.4% | 3.0% | 3.6% | 4.3% | 5.0% | 5.5% | 5.7% | 6.0% | 6.2% | 6.4% | 6.5% |  |  |  |  |
| 4. Birth & NPD | 0.9% | 1.6% | 2.2% | 2.7% | 3.4% | 4.1% | 4.8% | 5.2% | 5.5% | 5.7% | 5.9% | 6.1% | 6.3% |  |  |  |  |
| 5. Birth & HES <5 & NPD | 1.1% | 1.9% | 2.6% | 3.2% | 3.9% | 4.7% | 5.5% | 6.0% | 6.3% | 6.5% | 6.7% | 6.9% | 7.1% |  |  |  |  |
| 6. Birth & HES <5 & HES 5 to <16 | 1.2% | 2.0% | 2.8% | 3.4% | 4.2% | 5.1% | 6.0% | 6.6% | 6.9% | 7.2% | 7.4% | 7.6% | 7.9% |  |  |  |  |
| 7. Birth & HES <5 & HES 5 to <16 & NPD | 1.1% | 2.0% | 2.7% | 3.4% | 4.2% | 5.1% | 6.0% | 6.5% | 6.9% | 7.2% | 7.4% | 7.6% | 7.9% |  |  |  |  |
|  |  |  |  |  |  |  |  |  |  |  |  |  |  |  |  |  |  |
| **2008/9 & 2009/10** |  |  |  |  |  |  |  |  |  |  |  |  |  |  |  |  |  |
| 1. HES birth record only | 1.1% | 1.8% | 2.4% | 2.9% | 3.5% | 4.1% | 4.7% | 5.1% | 5.3% | 5.5% | 5.7% |  |  |  |  |  |  |
| 2. Birth & HES <5 | 1.2% | 2.0% | 2.6% | 3.3% | 3.9% | 4.7% | 5.3% | 5.8% | 6.0% | 6.3% | 6.5% |  |  |  |  |  |  |
| 3. Birth & HES <16 | 1.1% | 1.9% | 2.5% | 3.0% | 3.7% | 4.3% | 5.0% | 5.4% | 5.7% | 5.9% | 6.1% |  |  |  |  |  |  |
| 4. Birth & NPD | 1.0% | 1.7% | 2.3% | 2.8% | 3.4% | 4.1% | 4.7% | 5.2% | 5.5% | 5.7% | 5.9% |  |  |  |  |  |  |
| 5. Birth & HES <5 & NPD | 1.1% | 1.9% | 2.5% | 3.1% | 3.8% | 4.6% | 5.3% | 5.7% | 6.0% | 6.3% | 6.5% |  |  |  |  |  |  |
| 6. Birth & HES <5 & HES 5 to <16 | 1.2% | 2.1% | 2.8% | 3.5% | 4.3% | 5.2% | 6.0% | 6.6% | 6.9% | 7.2% | 7.5% |  |  |  |  |  |  |
| 7. Birth & HES <5 & HES 5 to <16 & NPD | 1.2% | 2.0% | 2.8% | 3.5% | 4.3% | 5.2% | 6.0% | 6.5% | 6.9% | 7.2% | 7.4% |  |  |  |  |  |  |
|  |  |  |  |  |  |  |  |  |  |  |  |  |  |  |  |  |  |
| **2010/11 & 2011/12** |  |  |  |  |  |  |  |  |  |  |  |  |  |  |  |  |  |
| 1. HES birth record only | 1.2% | 1.9% | 2.4% | 3.0% | 3.5% | 4.1% | 4.7% | 5.0% | 5.3% |  |  |  |  |  |  |  |  |
| 2. Birth & HES <5 | 1.2% | 2.0% | 2.7% | 3.3% | 3.9% | 4.6% | 5.2% | 5.6% | 5.8% |  |  |  |  |  |  |  |  |
| 3. Birth & HES <16 | 1.2% | 1.9% | 2.5% | 3.1% | 3.7% | 4.3% | 4.9% | 5.3% | 5.6% |  |  |  |  |  |  |  |  |
| 4. Birth & NPD | 1.0% | 1.7% | 2.3% | 2.9% | 3.4% | 4.1% | 4.7% | 5.1% | 5.3% |  |  |  |  |  |  |  |  |
| 5. Birth & HES <5 & NPD | 1.1% | 1.9% | 2.5% | 3.1% | 3.8% | 4.5% | 5.1% | 5.5% | 5.8% |  |  |  |  |  |  |  |  |
| 6. Birth & HES <5 & HES 5 to <16 | 1.3% | 2.3% | 3.0% | 3.7% | 4.6% | 5.5% | 6.3% | 6.9% | 7.2% |  |  |  |  |  |  |  |  |
| 7. Birth & HES <5 & HES 5 to <16 & NPD | 1.3% | 2.2% | 3.0% | 3.7% | 4.5% | 5.4% | 6.3% | 6.8% | 7.2% |  |  |  |  |  |  |  |  |
|  |  |  |  |  |  |  |  |  |  |  |  |  |  |  |  |  |  |
| **Non-specific codes** | | | | | | | | | | | | | | | | | |
| **2002/3 & 2003/4** | | | | | | | | | | | | | | | | | |
| 1. HES birth record only | 0.2% | 1.0% | 1.2% | 1.3% | 1.4% | 1.4% | 1.5% | 1.5% | 1.5% | 1.6% | 1.6% | 1.6% | 1.6% | 1.7% | 1.7% | 1.7% | 1.7% |
| 2. Birth & HES <5 | 0.3% | 1.6% | 1.9% | 2.1% | 2.2% | 2.3% | 2.4% | 2.5% | 2.5% | 2.5% | 2.6% | 2.6% | 2.7% | 2.7% | 2.7% | 2.8% | 2.8% |
| 3. Birth & HES <16 | 0.2% | 1.2% | 1.4% | 1.6% | 1.7% | 1.8% | 1.8% | 1.9% | 1.9% | 1.9% | 2.0% | 2.0% | 2.0% | 2.0% | 2.1% | 2.1% | 2.1% |
| 4. Birth & NPD | 0.2% | 1.1% | 1.3% | 1.4% | 1.5% | 1.6% | 1.7% | 1.7% | 1.8% | 1.8% | 1.8% | 1.8% | 1.9% | 1.9% | 1.9% | 2.0% | 2.0% |
| 5. Birth & HES <5 & NPD | 0.3% | 1.5% | 1.8% | 2.0% | 2.2% | 2.3% | 2.3% | 2.4% | 2.4% | 2.5% | 2.5% | 2.6% | 2.6% | 2.6% | 2.7% | 2.7% | 2.7% |
| 6. Birth & HES <5 & HES 5 to <16 | 0.3% | 1.5% | 1.9% | 2.1% | 2.2% | 2.4% | 2.4% | 2.5% | 2.6% | 2.6% | 2.6% | 2.7% | 2.7% | 2.7% | 2.8% | 2.8% | 2.9% |
| 7. Birth & HES <5 & HES 5 to <16 & NPD | 0.3% | 1.5% | 1.9% | 2.1% | 2.2% | 2.3% | 2.4% | 2.5% | 2.5% | 2.6% | 2.6% | 2.7% | 2.7% | 2.7% | 2.8% | 2.8% | 2.9% |
|  |  |  |  |  |  |  |  |  |  |  |  |  |  |  |  |  |  |
| **2004/5 & 2005/6** |  |  |  |  |  |  |  |  |  |  |  |  |  |  |  |  |  |
| 1. HES birth record only | 0.2% | 1.0% | 1.3% | 1.4% | 1.5% | 1.6% | 1.6% | 1.7% | 1.7% | 1.7% | 1.7% | 1.8% | 1.8% | 1.8% | 1.9% |  |  |
| 2. Birth & HES <5 | 0.2% | 1.4% | 1.8% | 1.9% | 2.1% | 2.2% | 2.2% | 2.3% | 2.3% | 2.4% | 2.4% | 2.4% | 2.5% | 2.5% | 2.5% |  |  |
| 3. Birth & HES <16 | 0.2% | 1.2% | 1.4% | 1.6% | 1.7% | 1.8% | 1.8% | 1.9% | 1.9% | 1.9% | 2.0% | 2.0% | 2.0% | 2.0% | 2.1% |  |  |
| 4. Birth & NPD | 0.2% | 1.1% | 1.3% | 1.5% | 1.6% | 1.6% | 1.7% | 1.7% | 1.8% | 1.8% | 1.8% | 1.9% | 1.9% | 1.9% | 2.0% |  |  |
| 5. Birth & HES <5 & NPD | 0.2% | 1.4% | 1.7% | 1.9% | 2.0% | 2.1% | 2.2% | 2.2% | 2.3% | 2.3% | 2.3% | 2.4% | 2.4% | 2.4% | 2.5% |  |  |
| 6. Birth & HES <5 & HES 5 to <16 | 0.2% | 1.4% | 1.8% | 2.0% | 2.1% | 2.2% | 2.3% | 2.4% | 2.4% | 2.4% | 2.5% | 2.5% | 2.5% | 2.6% | 2.6% |  |  |
| 7. Birth & HES <5 & HES 5 to <16 & NPD | 0.2% | 1.4% | 1.8% | 1.9% | 2.1% | 2.2% | 2.3% | 2.3% | 2.4% | 2.4% | 2.4% | 2.5% | 2.5% | 2.6% | 2.6% |  |  |
|  |  |  |  |  |  |  |  |  |  |  |  |  |  |  |  |  |  |
| **2006/7 & 2007/8** |  |  |  |  |  |  |  |  |  |  |  |  |  |  |  |  |  |
| 1. HES birth record only | 0.2% | 1.0% | 1.3% | 1.4% | 1.5% | 1.6% | 1.7% | 1.7% | 1.7% | 1.7% | 1.8% | 1.8% | 1.8% |  |  |  |  |
| 2. Birth & HES <5 | 0.2% | 1.3% | 1.6% | 1.8% | 1.9% | 2.0% | 2.1% | 2.1% | 2.2% | 2.2% | 2.2% | 2.3% | 2.3% |  |  |  |  |
| 3. Birth & HES <16 | 0.2% | 1.2% | 1.4% | 1.6% | 1.7% | 1.8% | 1.9% | 1.9% | 1.9% | 2.0% | 2.0% | 2.0% | 2.1% |  |  |  |  |
| 4. Birth & NPD | 0.2% | 1.1% | 1.3% | 1.5% | 1.6% | 1.7% | 1.7% | 1.8% | 1.8% | 1.8% | 1.9% | 1.9% | 1.9% |  |  |  |  |
| 5. Birth & HES <5 & NPD | 0.2% | 1.3% | 1.6% | 1.7% | 1.9% | 2.0% | 2.0% | 2.1% | 2.1% | 2.2% | 2.2% | 2.2% | 2.3% |  |  |  |  |
| 6. Birth & HES <5 & HES 5 to <16 | 0.2% | 1.3% | 1.7% | 1.9% | 2.0% | 2.1% | 2.2% | 2.3% | 2.3% | 2.3% | 2.4% | 2.4% | 2.5% |  |  |  |  |
| 7. Birth & HES <5 & HES 5 to <16 & NPD | 0.2% | 1.3% | 1.7% | 1.9% | 2.0% | 2.1% | 2.2% | 2.2% | 2.3% | 2.3% | 2.4% | 2.4% | 2.4% |  |  |  |  |
|  |  |  |  |  |  |  |  |  |  |  |  |  |  |  |  |  |  |
| **2008/9 & 2009/10** |  |  |  |  |  |  |  |  |  |  |  |  |  |  |  |  |  |
| 1. HES birth record only | 0.2% | 1.2% | 1.4% | 1.6% | 1.7% | 1.7% | 1.8% | 1.8% | 1.9% | 1.9% | 2.0% |  |  |  |  |  |  |
| 2. Birth & HES <5 | 0.3% | 1.3% | 1.7% | 1.8% | 2.0% | 2.0% | 2.1% | 2.2% | 2.2% | 2.2% | 2.3% |  |  |  |  |  |  |
| 3. Birth & HES <16 | 0.2% | 1.2% | 1.5% | 1.7% | 1.8% | 1.9% | 2.0% | 2.0% | 2.0% | 2.1% | 2.1% |  |  |  |  |  |  |
| 4. Birth & NPD | 0.2% | 1.2% | 1.4% | 1.6% | 1.7% | 1.8% | 1.8% | 1.9% | 1.9% | 2.0% | 2.0% |  |  |  |  |  |  |
| 5. Birth & HES <5 & NPD | 0.2% | 1.3% | 1.6% | 1.8% | 1.9% | 2.0% | 2.1% | 2.1% | 2.1% | 2.2% | 2.2% |  |  |  |  |  |  |
| 6. Birth & HES <5 & HES 5 to <16 | 0.2% | 1.4% | 1.8% | 2.0% | 2.1% | 2.2% | 2.3% | 2.4% | 2.4% | 2.5% | 2.5% |  |  |  |  |  |  |
| 7. Birth & HES <5 & HES 5 to <16 & NPD | 0.2% | 1.4% | 1.8% | 2.0% | 2.1% | 2.2% | 2.3% | 2.4% | 2.4% | 2.5% | 2.5% |  |  |  |  |  |  |
|  |  |  |  |  |  |  |  |  |  |  |  |  |  |  |  |  |  |
| **2010/11 & 2011/12** |  |  |  |  |  |  |  |  |  |  |  |  |  |  |  |  |  |
| 1. HES birth record only | 0.2% | 1.2% | 1.5% | 1.6% | 1.7% | 1.8% | 1.9% | 1.9% | 2.0% |  |  |  |  |  |  |  |  |
| 2. Birth & HES <5 | 0.3% | 1.4% | 1.7% | 1.8% | 1.9% | 2.0% | 2.1% | 2.2% | 2.2% |  |  |  |  |  |  |  |  |
| 3. Birth & HES <16 | 0.3% | 1.3% | 1.6% | 1.7% | 1.8% | 1.9% | 2.0% | 2.1% | 2.1% |  |  |  |  |  |  |  |  |
| 4. Birth & NPD | 0.2% | 1.2% | 1.5% | 1.6% | 1.7% | 1.8% | 1.9% | 1.9% | 2.0% |  |  |  |  |  |  |  |  |
| 5. Birth & HES <5 & NPD | 0.3% | 1.3% | 1.6% | 1.8% | 1.9% | 2.0% | 2.1% | 2.1% | 2.2% |  |  |  |  |  |  |  |  |
| 6. Birth & HES <5 & HES 5 to <16 | 0.3% | 1.5% | 1.9% | 2.1% | 2.3% | 2.4% | 2.5% | 2.6% | 2.6% |  |  |  |  |  |  |  |  |
| 7. Birth & HES <5 & HES 5 to <16 & NPD | 0.3% | 1.5% | 1.9% | 2.1% | 2.2% | 2.4% | 2.5% | 2.5% | 2.6% |  |  |  |  |  |  |  |  |
|  |  |  |  |  |  |  |  |  |  |  |  |  |  |  |  |  |  |
| **Respiratory** | | | | | | | | | | | | | | | | | |
| **2002/3 & 2003/4** | | | | | | | | | | | | | | | | | |
| 1. HES birth record only | 0.4% | 0.9% | 1.4% | 2.0% | 2.6% | 3.1% | 3.6% | 3.9% | 4.2% | 4.5% | 4.8% | 5.1% | 5.3% | 5.6% | 5.9% | 6.2% | 6.4% |
| 2. Birth & HES <5 | 0.6% | 1.4% | 2.3% | 3.2% | 4.2% | 5.0% | 5.7% | 6.2% | 6.6% | 7.0% | 7.4% | 7.8% | 8.1% | 8.5% | 8.9% | 9.2% | 9.6% |
| 3. Birth & HES <16 | 0.5% | 1.0% | 1.7% | 2.4% | 3.1% | 3.8% | 4.3% | 4.8% | 5.2% | 5.5% | 5.9% | 6.2% | 6.5% | 6.9% | 7.2% | 7.6% | 7.9% |
| 4. Birth & NPD | 0.4% | 0.9% | 1.6% | 2.3% | 3.0% | 3.6% | 4.2% | 4.6% | 5.0% | 5.3% | 5.7% | 6.0% | 6.3% | 6.7% | 7.0% | 7.3% | 7.7% |
| 5. Birth & HES <5 & NPD | 0.6% | 1.3% | 2.2% | 3.2% | 4.2% | 5.0% | 5.7% | 6.2% | 6.7% | 7.1% | 7.5% | 7.9% | 8.3% | 8.6% | 9.0% | 9.4% | 9.8% |
| 6. Birth & HES <5 & HES 5 to <16 | 0.6% | 1.4% | 2.3% | 3.3% | 4.3% | 5.2% | 6.0% | 6.5% | 7.0% | 7.5% | 7.9% | 8.3% | 8.7% | 9.1% | 9.5% | 10.0% | 10.3% |
| 7. Birth & HES <5 & HES 5 to <16 & NPD | 0.6% | 1.3% | 2.3% | 3.3% | 4.3% | 5.2% | 5.9% | 6.5% | 7.0% | 7.4% | 7.9% | 8.3% | 8.7% | 9.1% | 9.5% | 10.0% | 10.3% |
|  |  |  |  |  |  |  |  |  |  |  |  |  |  |  |  |  |  |
| **2004/5 & 2005/6** |  |  |  |  |  |  |  |  |  |  |  |  |  |  |  |  |  |
| 1. HES birth record only | 0.4% | 0.9% | 1.5% | 2.1% | 2.8% | 3.4% | 4.0% | 4.4% | 4.8% | 5.1% | 5.4% | 5.7% | 6.0% | 6.3% | 6.6% |  |  |
| 2. Birth & HES <5 | 0.5% | 1.2% | 2.0% | 2.8% | 3.8% | 4.7% | 5.4% | 5.9% | 6.4% | 6.8% | 7.1% | 7.5% | 7.8% | 8.2% | 8.5% |  |  |
| 3. Birth & HES <16 | 0.4% | 1.0% | 1.6% | 2.3% | 3.1% | 3.8% | 4.4% | 4.9% | 5.4% | 5.7% | 6.1% | 6.4% | 6.7% | 7.0% | 7.4% |  |  |
| 4. Birth & NPD | 0.4% | 0.9% | 1.5% | 2.2% | 2.9% | 3.7% | 4.3% | 4.8% | 5.2% | 5.5% | 5.8% | 6.2% | 6.5% | 6.8% | 7.1% |  |  |
| 5. Birth & HES <5 & NPD | 0.5% | 1.1% | 1.9% | 2.8% | 3.7% | 4.7% | 5.4% | 5.9% | 6.4% | 6.8% | 7.2% | 7.5% | 7.9% | 8.3% | 8.6% |  |  |
| 6. Birth & HES <5 & HES 5 to <16 | 0.5% | 1.2% | 2.0% | 2.9% | 3.9% | 4.9% | 5.7% | 6.3% | 6.8% | 7.3% | 7.7% | 8.1% | 8.5% | 8.9% | 9.2% |  |  |
| 7. Birth & HES <5 & HES 5 to <16 & NPD | 0.5% | 1.1% | 2.0% | 2.9% | 3.9% | 4.9% | 5.7% | 6.3% | 6.8% | 7.3% | 7.7% | 8.1% | 8.4% | 8.8% | 9.2% |  |  |
|  |  |  |  |  |  |  |  |  |  |  |  |  |  |  |  |  |  |
| **2006/7 & 2007/8** |  |  |  |  |  |  |  |  |  |  |  |  |  |  |  |  |  |
| 1. HES birth record only | 0.5% | 0.9% | 1.5% | 2.1% | 2.8% | 3.5% | 4.1% | 4.6% | 5.0% | 5.3% | 5.6% | 5.9% | 6.1% |  |  |  |  |
| 2. Birth & HES <5 | 0.6% | 1.1% | 1.8% | 2.6% | 3.5% | 4.4% | 5.2% | 5.7% | 6.2% | 6.5% | 6.9% | 7.2% | 7.5% |  |  |  |  |
| 3. Birth & HES <16 | 0.5% | 1.0% | 1.6% | 2.3% | 3.1% | 3.9% | 4.6% | 5.1% | 5.6% | 5.9% | 6.3% | 6.6% | 6.9% |  |  |  |  |
| 4. Birth & NPD | 0.5% | 0.9% | 1.5% | 2.2% | 3.0% | 3.8% | 4.5% | 5.0% | 5.4% | 5.8% | 6.1% | 6.4% | 6.7% |  |  |  |  |
| 5. Birth & HES <5 & NPD | 0.5% | 1.0% | 1.7% | 2.5% | 3.5% | 4.4% | 5.2% | 5.8% | 6.2% | 6.6% | 7.0% | 7.3% | 7.6% |  |  |  |  |
| 6. Birth & HES <5 & HES 5 to <16 | 0.6% | 1.2% | 1.9% | 2.8% | 3.8% | 4.8% | 5.6% | 6.3% | 6.8% | 7.3% | 7.7% | 8.1% | 8.4% |  |  |  |  |
| 7. Birth & HES <5 & HES 5 to <16 & NPD | 0.6% | 1.1% | 1.8% | 2.7% | 3.7% | 4.8% | 5.6% | 6.3% | 6.8% | 7.2% | 7.7% | 8.1% | 8.4% |  |  |  |  |
|  |  |  |  |  |  |  |  |  |  |  |  |  |  |  |  |  |  |
| **2008/9 & 2009/10** |  |  |  |  |  |  |  |  |  |  |  |  |  |  |  |  |  |
| 1. HES birth record only | 0.5% | 1.0% | 1.5% | 2.2% | 3.1% | 3.9% | 4.6% | 5.1% | 5.5% | 5.8% | 6.1% |  |  |  |  |  |  |
| 2. Birth & HES <5 | 0.6% | 1.1% | 1.8% | 2.6% | 3.6% | 4.5% | 5.3% | 5.9% | 6.3% | 6.7% | 7.0% |  |  |  |  |  |  |
| 3. Birth & HES <16 | 0.6% | 1.1% | 1.6% | 2.4% | 3.3% | 4.2% | 4.9% | 5.5% | 5.9% | 6.3% | 6.6% |  |  |  |  |  |  |
| 4. Birth & NPD | 0.5% | 0.9% | 1.5% | 2.2% | 3.2% | 4.0% | 4.8% | 5.3% | 5.7% | 6.1% | 6.5% |  |  |  |  |  |  |
| 5. Birth & HES <5 & NPD | 0.5% | 1.0% | 1.7% | 2.5% | 3.5% | 4.5% | 5.3% | 5.9% | 6.4% | 6.8% | 7.1% |  |  |  |  |  |  |
| 6. Birth & HES <5 & HES 5 to <16 | 0.6% | 1.2% | 1.9% | 2.9% | 4.0% | 5.1% | 6.1% | 6.8% | 7.3% | 7.8% | 8.3% |  |  |  |  |  |  |
| 7. Birth & HES <5 & HES 5 to <16 & NPD | 0.6% | 1.2% | 1.9% | 2.8% | 4.0% | 5.1% | 6.0% | 6.8% | 7.3% | 7.8% | 8.2% |  |  |  |  |  |  |
|  |  |  |  |  |  |  |  |  |  |  |  |  |  |  |  |  |  |
| **2010/11 & 2011/12** |  |  |  |  |  |  |  |  |  |  |  |  |  |  |  |  |  |
| 1. HES birth record only | 0.5% | 1.0% | 1.6% | 2.3% | 3.2% | 4.0% | 4.7% | 5.3% | 5.7% |  |  |  |  |  |  |  |  |
| 2. Birth & HES <5 | 0.6% | 1.1% | 1.7% | 2.6% | 3.6% | 4.5% | 5.3% | 5.9% | 6.3% |  |  |  |  |  |  |  |  |
| 3. Birth & HES <16 | 0.6% | 1.1% | 1.6% | 2.4% | 3.4% | 4.3% | 5.1% | 5.6% | 6.0% |  |  |  |  |  |  |  |  |
| 4. Birth & NPD | 0.5% | 1.0% | 1.5% | 2.3% | 3.2% | 4.1% | 4.9% | 5.4% | 5.8% |  |  |  |  |  |  |  |  |
| 5. Birth & HES <5 & NPD | 0.6% | 1.1% | 1.7% | 2.5% | 3.6% | 4.6% | 5.4% | 6.0% | 6.4% |  |  |  |  |  |  |  |  |
| 6. Birth & HES <5 & HES 5 to <16 | 0.7% | 1.3% | 2.0% | 3.0% | 4.3% | 5.5% | 6.6% | 7.4% | 8.0% |  |  |  |  |  |  |  |  |
| 7. Birth & HES <5 & HES 5 to <16 & NPD | 0.7% | 1.3% | 2.0% | 3.0% | 4.2% | 5.5% | 6.5% | 7.4% | 8.0% |  |  |  |  |  |  |  |  |
|  |  |  |  |  |  |  |  |  |  |  |  |  |  |  |  |  |  |

HES Hospital Episode Statistics; NPD National Pupil Database. See main text for explanation of each sensitivity analysis. The Metabolic, &c group includes metabolic, endocrine, digestive, renal and genitourinary conditions. See Supplementary Table S1 for a full list of codes by each group.

# Table S8. Proportions of children with two or more chronic health condition sub-types in each birth cohort (restricted to children in each closed cohort with at least one sub-type recorded; underlying data for Figure 3).

| Birth | Age | | | | | | | | | | | | | | | |
| --- | --- | --- | --- | --- | --- | --- | --- | --- | --- | --- | --- | --- | --- | --- | --- | --- |
| cohort | 0 | 1 | 2 | 3 | 4 | 5 | 6 | 7 | 8 | 9 | 10 | 11 | 12 | 13 | 14 | 15 |
|  |  |  |  |  |  |  |  |  |  |  |  |  |  |  |  |  |
|  |  |  |  |  |  |  |  |  |  |  |  |  |  |  |  |  |
| 2002/3 | 18.5% | 18.8% | 19.4% | 20.1% | 20.6% | 21.2% | 21.6% | 22.2% | 22.7% | 23.3% | 23.9% | 24.4% | 25.1% | 25.9% | 26.8% | 27.9% |
| 2003/4 | 18.4% | 18.8% | 19.4% | 20.2% | 20.8% | 21.3% | 22.0% | 22.6% | 23.1% | 23.7% | 24.3% | 24.8% | 25.5% | 26.3% | 27.3% | 28.5% |
| 2004/5 | 18.4% | 18.9% | 19.7% | 20.5% | 21.1% | 21.9% | 22.5% | 23.1% | 23.5% | 24.2% | 24.7% | 25.2% | 25.9% | 26.8% | 27.8% |  |
| 2005/6 | 18.9% | 19.3% | 20.0% | 20.8% | 21.6% | 22.3% | 22.8% | 23.3% | 23.9% | 24.4% | 24.9% | 25.5% | 26.2% | 27.1% |  |  |
| 2006/7 | 19.5% | 19.7% | 20.4% | 21.3% | 22.2% | 22.7% | 23.4% | 24.0% | 24.5% | 24.9% | 25.5% | 26.1% | 26.7% |  |  |  |
| 2008/9 | 19.5% | 20.2% | 21.1% | 22.1% | 22.9% | 23.6% | 24.1% | 24.6% | 25.0% | 25.5% | 26.1% | 26.7% |  |  |  |  |
| 2009/10 | 20.6% | 20.9% | 21.9% | 22.9% | 23.7% | 24.3% | 24.8% | 25.2% | 25.7% | 26.2% | 26.7% |  |  |  |  |  |
| 2010/11 | 21.0% | 21.5% | 22.4% | 23.5% | 24.1% | 24.7% | 25.1% | 25.6% | 26.1% | 26.7% |  |  |  |  |  |  |
| 2011/12 | 21.3% | 22.0% | 23.1% | 24.1% | 24.7% | 25.1% | 25.6% | 26.1% | 26.6% |  |  |  |  |  |  |  |
|  |  |  |  |  |  |  |  |  |  |  |  |  |  |  |  |  |

# Table S9. Cumulative incidence of being admitted to hospital and having any record indicating a chronic health condition before age 16 (closed cohorts, underlying data for Figure 3 and Supplementary Figure S6)

|  | By age | | | | | | | | | | | | | | | |
| --- | --- | --- | --- | --- | --- | --- | --- | --- | --- | --- | --- | --- | --- | --- | --- | --- |
| Birth cohort | 1 | 2 | 3 | 4 | 5 | 6 | 7 | 8 | 9 | 10 | 11 | 12 | 13 | 14 | 15 | 16 |
|  |  |  |  |  |  |  |  |  |  |  |  |  |  |  |  |  |
| Any chronic health condition |  |  |  |  |  |  |  |  |  |  |  |  |  |  |  |  |
|  |  |  |  |  |  |  |  |  |  |  |  |  |  |  |  |  |
| 2002/3 | 6.3% | 8.8% | 10.6% | 12.2% | 13.8% | 15.3% | 16.5% | 17.4% | 18.2% | 19.0% | 19.8% | 20.6% | 21.5% | 22.6% | 24.0% | 25.6% |
| 2003/4 | 6.2% | 8.6% | 10.4% | 12.0% | 13.7% | 15.1% | 16.3% | 17.3% | 18.1% | 18.9% | 19.7% | 20.5% | 21.4% | 22.5% | 23.9% | 25.5% |
| 2004/5 | 6.1% | 8.6% | 10.3% | 12.0% | 13.7% | 15.2% | 16.5% | 17.4% | 18.2% | 19.0% | 19.8% | 20.6% | 21.6% | 22.8% | 24.2% |  |
| 2005/6 | 6.4% | 8.7% | 10.5% | 12.2% | 13.8% | 15.3% | 16.5% | 17.4% | 18.2% | 19.0% | 19.7% | 20.5% | 21.4% | 22.6% |  |  |
| 2006/7 | 6.4% | 8.8% | 10.5% | 12.2% | 13.8% | 15.3% | 16.4% | 17.3% | 18.2% | 18.9% | 19.7% | 20.6% | 21.6% |  |  |  |
| 2008/9 | 6.5% | 8.8% | 10.7% | 12.4% | 14.1% | 15.6% | 16.8% | 17.7% | 18.5% | 19.3% | 20.1% | 21.0% |  |  |  |  |
| 2009/10 | 6.7% | 9.2% | 11.1% | 12.8% | 14.5% | 16.0% | 17.2% | 18.1% | 19.0% | 19.8% | 20.7% |  |  |  |  |  |
| 2010/11 | 6.9% | 9.3% | 11.0% | 12.7% | 14.4% | 15.9% | 17.1% | 18.1% | 19.0% | 19.9% |  |  |  |  |  |  |
| 2011/12 | 7.1% | 9.2% | 10.8% | 12.4% | 14.1% | 15.5% | 16.7% | 17.7% | 18.6% |  |  |  |  |  |  |  |
| 2012/13 | 6.7% | 8.4% | 9.9% | 11.5% | 13.1% | 14.6% | 15.8% | 16.8% |  |  |  |  |  |  |  |  |
| 2013/14 | 6.5% | 8.2% | 9.8% | 11.5% | 13.2% | 14.7% | 15.9% |  |  |  |  |  |  |  |  |  |
| 2014/15 | 6.6% | 8.2% | 9.7% | 11.4% | 13.0% | 14.5% |  |  |  |  |  |  |  |  |  |  |
| 2015/16 | 6.7% | 8.3% | 9.9% | 11.6% | 13.2% |  |  |  |  |  |  |  |  |  |  |  |
| 2016/17 | 6.8% | 8.5% | 10.0% | 11.7% |  |  |  |  |  |  |  |  |  |  |  |  |
| 2017/18 | 7.1% | 8.9% | 10.5% |  |  |  |  |  |  |  |  |  |  |  |  |  |
| 2018/19 | 7.4% | 9.2% |  |  |  |  |  |  |  |  |  |  |  |  |  |  |
|  |  |  |  |  |  |  |  |  |  |  |  |  |  |  |  |  |
| Cancer/ blood |  |  |  |  |  |  |  |  |  |  |  |  |  |  |  |  |
|  |  |  |  |  |  |  |  |  |  |  |  |  |  |  |  |  |
| 2002/3 | 0.3% | 0.4% | 0.6% | 0.7% | 0.8% | 0.9% | 1.1% | 1.2% | 1.3% | 1.4% | 1.5% | 1.7% | 1.8% | 1.9% | 2.0% | 2.1% |
| 2003/4 | 0.3% | 0.4% | 0.5% | 0.7% | 0.8% | 0.9% | 1.1% | 1.2% | 1.3% | 1.5% | 1.6% | 1.7% | 1.8% | 1.9% | 2.0% | 2.1% |
| 2004/5 | 0.3% | 0.5% | 0.6% | 0.7% | 0.9% | 1.0% | 1.2% | 1.3% | 1.5% | 1.6% | 1.7% | 1.8% | 1.9% | 2.0% | 2.1% |  |
| 2005/6 | 0.3% | 0.5% | 0.6% | 0.8% | 1.0% | 1.1% | 1.3% | 1.4% | 1.5% | 1.6% | 1.7% | 1.8% | 1.8% | 1.9% |  |  |
| 2006/7 | 0.3% | 0.5% | 0.6% | 0.8% | 1.0% | 1.1% | 1.3% | 1.4% | 1.5% | 1.6% | 1.7% | 1.8% | 1.9% |  |  |  |
| 2008/9 | 0.3% | 0.5% | 0.7% | 0.9% | 1.1% | 1.3% | 1.4% | 1.6% | 1.6% | 1.7% | 1.8% | 1.9% |  |  |  |  |
| 2009/10 | 0.4% | 0.6% | 0.7% | 0.9% | 1.2% | 1.3% | 1.5% | 1.6% | 1.6% | 1.7% | 1.8% |  |  |  |  |  |
| 2010/11 | 0.4% | 0.7% | 0.9% | 1.1% | 1.3% | 1.4% | 1.6% | 1.7% | 1.7% | 1.8% |  |  |  |  |  |  |
| 2011/12 | 0.5% | 0.8% | 1.0% | 1.2% | 1.3% | 1.5% | 1.6% | 1.7% | 1.7% |  |  |  |  |  |  |  |
| 2012/13 | 0.5% | 0.8% | 1.0% | 1.2% | 1.3% | 1.5% | 1.6% | 1.7% |  |  |  |  |  |  |  |  |
| 2013/14 | 0.6% | 0.9% | 1.1% | 1.3% | 1.4% | 1.6% | 1.7% |  |  |  |  |  |  |  |  |  |
| 2014/15 | 0.6% | 0.9% | 1.0% | 1.2% | 1.3% | 1.4% |  |  |  |  |  |  |  |  |  |  |
| 2015/16 | 0.6% | 0.8% | 0.9% | 1.1% | 1.2% |  |  |  |  |  |  |  |  |  |  |  |
| 2016/17 | 0.4% | 0.6% | 0.8% | 0.9% |  |  |  |  |  |  |  |  |  |  |  |  |
| 2017/18 | 0.4% | 0.7% | 0.9% |  |  |  |  |  |  |  |  |  |  |  |  |  |
| 2018/19 | 0.5% | 0.7% |  |  |  |  |  |  |  |  |  |  |  |  |  |  |
|  |  |  |  |  |  |  |  |  |  |  |  |  |  |  |  |  |
| Cardiovascular |  |  |  |  |  |  |  |  |  |  |  |  |  |  |  |  |
|  |  |  |  |  |  |  |  |  |  |  |  |  |  |  |  |  |
| 2002/3 | 1.1% | 1.2% | 1.3% | 1.3% | 1.4% | 1.4% | 1.4% | 1.5% | 1.5% | 1.5% | 1.6% | 1.6% | 1.6% | 1.7% | 1.8% | 1.8% |
| 2003/4 | 1.0% | 1.1% | 1.2% | 1.3% | 1.3% | 1.4% | 1.4% | 1.4% | 1.5% | 1.5% | 1.5% | 1.6% | 1.6% | 1.7% | 1.7% | 1.8% |
| 2004/5 | 1.0% | 1.1% | 1.2% | 1.3% | 1.3% | 1.4% | 1.4% | 1.4% | 1.5% | 1.5% | 1.5% | 1.6% | 1.6% | 1.7% | 1.8% |  |
| 2005/6 | 1.1% | 1.2% | 1.3% | 1.3% | 1.4% | 1.4% | 1.5% | 1.5% | 1.5% | 1.6% | 1.6% | 1.7% | 1.7% | 1.8% |  |  |
| 2006/7 | 1.1% | 1.2% | 1.3% | 1.4% | 1.4% | 1.5% | 1.5% | 1.6% | 1.6% | 1.6% | 1.7% | 1.7% | 1.8% |  |  |  |
| 2008/9 | 1.2% | 1.3% | 1.4% | 1.5% | 1.5% | 1.6% | 1.6% | 1.6% | 1.7% | 1.7% | 1.8% | 1.8% |  |  |  |  |
| 2009/10 | 1.3% | 1.4% | 1.5% | 1.5% | 1.6% | 1.6% | 1.7% | 1.7% | 1.8% | 1.8% | 1.9% |  |  |  |  |  |
| 2010/11 | 1.4% | 1.5% | 1.6% | 1.6% | 1.7% | 1.7% | 1.8% | 1.8% | 1.9% | 1.9% |  |  |  |  |  |  |
| 2011/12 | 1.5% | 1.6% | 1.6% | 1.7% | 1.8% | 1.8% | 1.9% | 1.9% | 2.0% |  |  |  |  |  |  |  |
| 2012/13 | 1.5% | 1.6% | 1.7% | 1.8% | 1.8% | 1.9% | 2.0% | 2.0% |  |  |  |  |  |  |  |  |
| 2013/14 | 1.5% | 1.7% | 1.8% | 1.8% | 1.9% | 2.0% | 2.0% |  |  |  |  |  |  |  |  |  |
| 2014/15 | 1.6% | 1.7% | 1.8% | 1.9% | 2.0% | 2.1% |  |  |  |  |  |  |  |  |  |  |
| 2015/16 | 1.6% | 1.7% | 1.8% | 1.9% | 2.0% |  |  |  |  |  |  |  |  |  |  |  |
| 2016/17 | 1.7% | 1.8% | 1.9% | 2.0% |  |  |  |  |  |  |  |  |  |  |  |  |
| 2017/18 | 1.8% | 1.9% | 2.0% |  |  |  |  |  |  |  |  |  |  |  |  |  |
| 2018/19 | 1.9% | 2.0% |  |  |  |  |  |  |  |  |  |  |  |  |  |  |
|  |  |  |  |  |  |  |  |  |  |  |  |  |  |  |  |  |
|  |  |  |  |  |  |  |  |  |  |  |  |  |  |  |  |  |
| Mental health/ behavioural |  |  |  |  |  |  |  |  |  |  |  |  |  |  |  |  |
|  |  |  |  |  |  |  |  |  |  |  |  |  |  |  |  |  |
| 2002/3 | ~0.0% | 0.1% | 0.2% | 0.3% | 0.4% | 0.5% | 0.6% | 0.8% | 1.0% | 1.2% | 1.4% | 1.6% | 2.0% | 2.5% | 3.4% | 4.5% |
| 2003/4 | ~0.0% | 0.1% | 0.2% | 0.3% | 0.4% | 0.5% | 0.7% | 0.9% | 1.1% | 1.3% | 1.5% | 1.8% | 2.2% | 2.8% | 3.7% | 4.8% |
| 2004/5 | ~0.0% | 0.1% | 0.2% | 0.3% | 0.5% | 0.6% | 0.8% | 1.0% | 1.2% | 1.4% | 1.7% | 1.9% | 2.3% | 3.0% | 3.9% |  |
| 2005/6 | ~0.0% | 0.1% | 0.2% | 0.3% | 0.5% | 0.7% | 0.9% | 1.1% | 1.3% | 1.5% | 1.8% | 2.1% | 2.5% | 3.1% |  |  |
| 2006/7 | ~0.0% | 0.1% | 0.2% | 0.4% | 0.6% | 0.8% | 1.0% | 1.2% | 1.4% | 1.7% | 1.9% | 2.2% | 2.7% |  |  |  |
| 2008/9 | ~0.0% | 0.1% | 0.2% | 0.4% | 0.7% | 0.9% | 1.1% | 1.4% | 1.6% | 1.8% | 2.1% | 2.4% |  |  |  |  |
| 2009/10 | ~0.0% | 0.1% | 0.3% | 0.5% | 0.8% | 1.0% | 1.3% | 1.5% | 1.8% | 2.1% | 2.4% |  |  |  |  |  |
| 2010/11 | ~0.0% | 0.2% | 0.4% | 0.7% | 0.9% | 1.2% | 1.4% | 1.7% | 2.0% | 2.3% |  |  |  |  |  |  |
| 2011/12 | ~0.0% | 0.2% | 0.4% | 0.7% | 1.0% | 1.3% | 1.6% | 1.9% | 2.2% |  |  |  |  |  |  |  |
| 2012/13 | 0.1% | 0.2% | 0.5% | 0.8% | 1.0% | 1.3% | 1.6% | 1.9% |  |  |  |  |  |  |  |  |
| 2013/14 | 0.1% | 0.3% | 0.5% | 0.9% | 1.2% | 1.5% | 1.8% |  |  |  |  |  |  |  |  |  |
| 2014/15 | 0.1% | 0.3% | 0.5% | 0.9% | 1.2% | 1.6% |  |  |  |  |  |  |  |  |  |  |
| 2015/16 | 0.1% | 0.3% | 0.6% | 1.0% | 1.3% |  |  |  |  |  |  |  |  |  |  |  |
| 2016/17 | 0.1% | 0.3% | 0.7% | 1.1% |  |  |  |  |  |  |  |  |  |  |  |  |
| 2017/18 | 0.1% | 0.3% | 0.7% |  |  |  |  |  |  |  |  |  |  |  |  |  |
| 2018/19 | 0.1% | 0.4% |  |  |  |  |  |  |  |  |  |  |  |  |  |  |
|  |  |  |  |  |  |  |  |  |  |  |  |  |  |  |  |  |
| Metabolic, &c |  |  |  |  |  |  |  |  |  |  |  |  |  |  |  |  |
|  |  |  |  |  |  |  |  |  |  |  |  |  |  |  |  |  |
| 2002/3 | 2.6% | 3.7% | 4.2% | 4.6% | 4.9% | 5.2% | 5.4% | 5.7% | 5.9% | 6.2% | 6.4% | 6.6% | 6.8% | 7.1% | 7.4% | 7.8% |
| 2003/4 | 2.6% | 3.8% | 4.3% | 4.7% | 5.0% | 5.2% | 5.5% | 5.8% | 6.0% | 6.2% | 6.4% | 6.6% | 6.9% | 7.2% | 7.5% | 7.9% |
| 2004/5 | 2.6% | 3.7% | 4.2% | 4.6% | 5.0% | 5.3% | 5.5% | 5.7% | 5.9% | 6.1% | 6.3% | 6.6% | 6.9% | 7.2% | 7.5% |  |
| 2005/6 | 2.8% | 3.9% | 4.5% | 4.9% | 5.2% | 5.5% | 5.7% | 5.9% | 6.1% | 6.3% | 6.5% | 6.7% | 7.0% | 7.3% |  |  |
| 2006/7 | 2.8% | 4.0% | 4.5% | 4.9% | 5.2% | 5.5% | 5.7% | 5.8% | 6.0% | 6.3% | 6.5% | 6.7% | 7.0% |  |  |  |
| 2008/9 | 2.8% | 3.9% | 4.5% | 4.9% | 5.2% | 5.4% | 5.6% | 5.8% | 6.0% | 6.2% | 6.5% | 6.7% |  |  |  |  |
| 2009/10 | 2.9% | 4.2% | 4.8% | 5.1% | 5.3% | 5.5% | 5.7% | 5.9% | 6.1% | 6.4% | 6.6% |  |  |  |  |  |
| 2010/11 | 3.1% | 4.3% | 4.7% | 4.9% | 5.1% | 5.3% | 5.5% | 5.8% | 6.0% | 6.2% |  |  |  |  |  |  |
| 2011/12 | 3.0% | 3.9% | 4.2% | 4.4% | 4.6% | 4.8% | 5.0% | 5.3% | 5.5% |  |  |  |  |  |  |  |
| 2012/13 | 2.5% | 2.9% | 3.2% | 3.4% | 3.6% | 3.9% | 4.1% | 4.3% |  |  |  |  |  |  |  |  |
| 2013/14 | 2.0% | 2.4% | 2.7% | 3.0% | 3.2% | 3.5% | 3.7% |  |  |  |  |  |  |  |  |  |
| 2014/15 | 2.1% | 2.5% | 2.8% | 3.1% | 3.3% | 3.6% |  |  |  |  |  |  |  |  |  |  |
| 2015/16 | 2.2% | 2.7% | 3.0% | 3.2% | 3.5% |  |  |  |  |  |  |  |  |  |  |  |
| 2016/17 | 2.4% | 2.8% | 3.1% | 3.4% |  |  |  |  |  |  |  |  |  |  |  |  |
| 2017/18 | 2.5% | 3.0% | 3.4% |  |  |  |  |  |  |  |  |  |  |  |  |  |
| 2018/19 | 2.7% | 3.3% |  |  |  |  |  |  |  |  |  |  |  |  |  |  |
|  |  |  |  |  |  |  |  |  |  |  |  |  |  |  |  |  |
| Musculoskeletal/ skin |  |  |  |  |  |  |  |  |  |  |  |  |  |  |  |  |
|  |  |  |  |  |  |  |  |  |  |  |  |  |  |  |  |  |
| 2002/3 | 0.5% | 0.7% | 0.8% | 0.9% | 1.0% | 1.1% | 1.2% | 1.3% | 1.4% | 1.6% | 1.7% | 1.9% | 2.1% | 2.3% | 2.6% | 2.9% |
| 2003/4 | 0.5% | 0.7% | 0.8% | 0.9% | 1.0% | 1.1% | 1.2% | 1.4% | 1.5% | 1.6% | 1.7% | 1.9% | 2.1% | 2.4% | 2.7% | 3.0% |
| 2004/5 | 0.5% | 0.7% | 0.8% | 1.0% | 1.1% | 1.2% | 1.3% | 1.4% | 1.5% | 1.7% | 1.8% | 2.0% | 2.2% | 2.5% | 2.8% |  |
| 2005/6 | 0.5% | 0.7% | 0.9% | 1.0% | 1.1% | 1.2% | 1.4% | 1.5% | 1.6% | 1.7% | 1.9% | 2.0% | 2.3% | 2.5% |  |  |
| 2006/7 | 0.5% | 0.7% | 0.9% | 1.0% | 1.1% | 1.2% | 1.4% | 1.5% | 1.6% | 1.7% | 1.9% | 2.1% | 2.3% |  |  |  |
| 2008/9 | 0.5% | 0.7% | 0.9% | 1.0% | 1.1% | 1.3% | 1.4% | 1.5% | 1.6% | 1.8% | 1.9% | 2.1% |  |  |  |  |
| 2009/10 | 0.5% | 0.7% | 0.9% | 1.0% | 1.2% | 1.3% | 1.4% | 1.6% | 1.7% | 1.9% | 2.0% |  |  |  |  |  |
| 2010/11 | 0.6% | 0.8% | 1.0% | 1.1% | 1.2% | 1.4% | 1.5% | 1.6% | 1.8% | 1.9% |  |  |  |  |  |  |
| 2011/12 | 0.6% | 0.8% | 1.0% | 1.2% | 1.3% | 1.4% | 1.6% | 1.7% | 1.8% |  |  |  |  |  |  |  |
| 2012/13 | 0.6% | 0.8% | 1.0% | 1.2% | 1.3% | 1.4% | 1.6% | 1.7% |  |  |  |  |  |  |  |  |
| 2013/14 | 0.6% | 0.8% | 1.0% | 1.2% | 1.3% | 1.5% | 1.6% |  |  |  |  |  |  |  |  |  |
| 2014/15 | 0.7% | 0.9% | 1.1% | 1.3% | 1.4% | 1.6% |  |  |  |  |  |  |  |  |  |  |
| 2015/16 | 0.7% | 0.9% | 1.1% | 1.3% | 1.4% |  |  |  |  |  |  |  |  |  |  |  |
| 2016/17 | 0.7% | 0.9% | 1.2% | 1.3% |  |  |  |  |  |  |  |  |  |  |  |  |
| 2017/18 | 0.7% | 1.0% | 1.2% |  |  |  |  |  |  |  |  |  |  |  |  |  |
| 2018/19 | 0.7% | 1.0% |  |  |  |  |  |  |  |  |  |  |  |  |  |  |
|  |  |  |  |  |  |  |  |  |  |  |  |  |  |  |  |  |
|  |  |  |  |  |  |  |  |  |  |  |  |  |  |  |  |  |
|  |  |  |  |  |  |  |  |  |  |  |  |  |  |  |  |  |
| Neurological |  |  |  |  |  |  |  |  |  |  |  |  |  |  |  |  |
|  |  |  |  |  |  |  |  |  |  |  |  |  |  |  |  |  |
| 2002/3 | 1.7% | 2.3% | 2.9% | 3.5% | 4.3% | 5.1% | 5.6% | 5.9% | 6.2% | 6.4% | 6.6% | 6.8% | 7.0% | 7.2% | 7.5% | 7.8% |
| 2003/4 | 1.6% | 2.3% | 2.8% | 3.5% | 4.3% | 5.1% | 5.6% | 5.9% | 6.2% | 6.4% | 6.6% | 6.8% | 7.0% | 7.3% | 7.6% | 7.9% |
| 2004/5 | 1.6% | 2.3% | 2.9% | 3.6% | 4.3% | 5.1% | 5.6% | 5.9% | 6.2% | 6.4% | 6.6% | 6.8% | 7.0% | 7.3% | 7.6% |  |
| 2005/6 | 1.7% | 2.3% | 2.9% | 3.5% | 4.2% | 4.9% | 5.4% | 5.7% | 5.9% | 6.1% | 6.3% | 6.5% | 6.7% | 7.0% |  |  |
| 2006/7 | 1.7% | 2.3% | 2.8% | 3.4% | 4.1% | 4.8% | 5.3% | 5.7% | 5.9% | 6.1% | 6.3% | 6.5% | 6.8% |  |  |  |
| 2008/9 | 1.7% | 2.3% | 2.9% | 3.5% | 4.2% | 4.9% | 5.4% | 5.7% | 5.9% | 6.2% | 6.4% | 6.6% |  |  |  |  |
| 2009/10 | 1.8% | 2.4% | 3.0% | 3.6% | 4.4% | 5.0% | 5.5% | 5.8% | 6.0% | 6.3% | 6.5% |  |  |  |  |  |
| 2010/11 | 1.8% | 2.5% | 3.0% | 3.6% | 4.3% | 5.0% | 5.4% | 5.7% | 6.0% | 6.2% |  |  |  |  |  |  |
| 2011/12 | 1.9% | 2.5% | 3.1% | 3.6% | 4.3% | 4.9% | 5.3% | 5.6% | 5.9% |  |  |  |  |  |  |  |
| 2012/13 | 1.9% | 2.5% | 3.0% | 3.6% | 4.3% | 4.8% | 5.3% | 5.5% |  |  |  |  |  |  |  |  |
| 2013/14 | 2.0% | 2.6% | 3.1% | 3.7% | 4.3% | 4.9% | 5.3% |  |  |  |  |  |  |  |  |  |
| 2014/15 | 2.0% | 2.6% | 3.2% | 3.7% | 4.3% | 4.8% |  |  |  |  |  |  |  |  |  |  |
| 2015/16 | 2.1% | 2.6% | 3.2% | 3.8% | 4.3% |  |  |  |  |  |  |  |  |  |  |  |
| 2016/17 | 2.1% | 2.7% | 3.3% | 3.8% |  |  |  |  |  |  |  |  |  |  |  |  |
| 2017/18 | 2.3% | 2.9% | 3.4% |  |  |  |  |  |  |  |  |  |  |  |  |  |
| 2018/19 | 2.3% | 2.9% |  |  |  |  |  |  |  |  |  |  |  |  |  |  |
|  |  |  |  |  |  |  |  |  |  |  |  |  |  |  |  |  |
| Non-specific |  |  |  |  |  |  |  |  |  |  |  |  |  |  |  |  |
|  |  |  |  |  |  |  |  |  |  |  |  |  |  |  |  |  |
| 2002/3 | 1.0% | 1.2% | 1.4% | 1.5% | 1.5% | 1.6% | 1.6% | 1.7% | 1.7% | 1.7% | 1.8% | 1.8% | 1.8% | 1.9% | 1.9% | 1.9% |
| 2003/4 | 1.0% | 1.3% | 1.4% | 1.5% | 1.6% | 1.7% | 1.7% | 1.8% | 1.8% | 1.8% | 1.9% | 1.9% | 1.9% | 2.0% | 2.0% | 2.1% |
| 2004/5 | 1.0% | 1.3% | 1.4% | 1.5% | 1.6% | 1.7% | 1.7% | 1.8% | 1.8% | 1.8% | 1.9% | 1.9% | 1.9% | 2.0% | 2.0% |  |
| 2005/6 | 1.0% | 1.3% | 1.5% | 1.6% | 1.7% | 1.7% | 1.8% | 1.8% | 1.9% | 1.9% | 1.9% | 2.0% | 2.0% | 2.0% |  |  |
| 2006/7 | 1.0% | 1.3% | 1.5% | 1.6% | 1.7% | 1.8% | 1.8% | 1.9% | 1.9% | 1.9% | 2.0% | 2.0% | 2.1% |  |  |  |
| 2008/9 | 1.1% | 1.3% | 1.5% | 1.7% | 1.7% | 1.8% | 1.9% | 1.9% | 1.9% | 2.0% | 2.0% | 2.0% |  |  |  |  |
| 2009/10 | 1.1% | 1.4% | 1.6% | 1.7% | 1.8% | 1.9% | 2.0% | 2.0% | 2.0% | 2.1% | 2.1% |  |  |  |  |  |
| 2010/11 | 1.1% | 1.5% | 1.6% | 1.8% | 1.9% | 1.9% | 2.0% | 2.1% | 2.1% | 2.2% |  |  |  |  |  |  |
| 2011/12 | 1.2% | 1.5% | 1.7% | 1.8% | 1.9% | 2.0% | 2.0% | 2.1% | 2.1% |  |  |  |  |  |  |  |
| 2012/13 | 1.2% | 1.5% | 1.7% | 1.8% | 1.9% | 2.0% | 2.0% | 2.1% |  |  |  |  |  |  |  |  |
| 2013/14 | 1.2% | 1.5% | 1.7% | 1.8% | 1.9% | 2.0% | 2.1% |  |  |  |  |  |  |  |  |  |
| 2014/15 | 1.2% | 1.5% | 1.7% | 1.8% | 2.0% | 2.0% |  |  |  |  |  |  |  |  |  |  |
| 2015/16 | 1.2% | 1.5% | 1.7% | 1.9% | 2.0% |  |  |  |  |  |  |  |  |  |  |  |
| 2016/17 | 1.3% | 1.6% | 1.8% | 2.0% |  |  |  |  |  |  |  |  |  |  |  |  |
| 2017/18 | 1.4% | 1.7% | 1.9% |  |  |  |  |  |  |  |  |  |  |  |  |  |
| 2018/19 | 1.5% | 1.9% |  |  |  |  |  |  |  |  |  |  |  |  |  |  |
|  |  |  |  |  |  |  |  |  |  |  |  |  |  |  |  |  |
| Respiratory |  |  |  |  |  |  |  |  |  |  |  |  |  |  |  |  |
|  |  |  |  |  |  |  |  |  |  |  |  |  |  |  |  |  |
| 2002/3 | 1.0% | 1.6% | 2.3% | 3.0% | 3.7% | 4.2% | 4.6% | 5.0% | 5.4% | 5.7% | 6.0% | 6.4% | 6.7% | 7.1% | 7.4% | 7.8% |
| 2003/4 | 0.9% | 1.6% | 2.2% | 2.9% | 3.6% | 4.1% | 4.6% | 5.0% | 5.3% | 5.7% | 6.0% | 6.4% | 6.7% | 7.0% | 7.4% | 7.7% |
| 2004/5 | 0.9% | 1.5% | 2.2% | 3.0% | 3.6% | 4.2% | 4.8% | 5.2% | 5.5% | 5.9% | 6.2% | 6.5% | 6.9% | 7.2% | 7.6% |  |
| 2005/6 | 0.9% | 1.5% | 2.2% | 2.9% | 3.6% | 4.3% | 4.8% | 5.2% | 5.6% | 5.9% | 6.2% | 6.5% | 6.8% | 7.2% |  |  |
| 2006/7 | 0.9% | 1.5% | 2.1% | 2.9% | 3.6% | 4.3% | 4.8% | 5.2% | 5.6% | 5.9% | 6.2% | 6.6% | 6.9% |  |  |  |
| 2008/9 | 0.9% | 1.5% | 2.2% | 3.0% | 3.8% | 4.5% | 5.1% | 5.5% | 5.9% | 6.2% | 6.5% | 6.9% |  |  |  |  |
| 2009/10 | 1.0% | 1.6% | 2.4% | 3.2% | 4.1% | 4.8% | 5.4% | 5.8% | 6.2% | 6.6% | 6.9% |  |  |  |  |  |
| 2010/11 | 1.0% | 1.6% | 2.3% | 3.2% | 4.1% | 4.9% | 5.5% | 5.9% | 6.3% | 6.7% |  |  |  |  |  |  |
| 2011/12 | 1.0% | 1.6% | 2.3% | 3.2% | 4.1% | 4.8% | 5.4% | 5.9% | 6.2% |  |  |  |  |  |  |  |
| 2012/13 | 1.1% | 1.6% | 2.3% | 3.2% | 4.1% | 4.8% | 5.4% | 5.8% |  |  |  |  |  |  |  |  |
| 2013/14 | 1.2% | 1.7% | 2.4% | 3.4% | 4.3% | 5.0% | 5.6% |  |  |  |  |  |  |  |  |  |
| 2014/15 | 1.1% | 1.6% | 2.3% | 3.2% | 4.1% | 4.8% |  |  |  |  |  |  |  |  |  |  |
| 2015/16 | 1.2% | 1.7% | 2.4% | 3.3% | 4.2% |  |  |  |  |  |  |  |  |  |  |  |
| 2016/17 | 1.2% | 1.7% | 2.4% | 3.3% |  |  |  |  |  |  |  |  |  |  |  |  |
| 2017/18 | 1.3% | 1.8% | 2.5% |  |  |  |  |  |  |  |  |  |  |  |  |  |
| 2018/19 | 1.3% | 1.9% |  |  |  |  |  |  |  |  |  |  |  |  |  |  |
|  |  |  |  |  |  |  |  |  |  |  |  |  |  |  |  |  |

The Metabolic, &c group includes metabolic, endocrine, digestive, renal and genitourinary conditions. See Supplementary Table S1 for a full list of codes by each group.
